# A Novel “Three‐in‐One” Copper‐Based Metal‐Organic Framework Nanozyme Eradicates Colorectal Cancer and Overcomes Chemoresistance for Tumor Therapy

**DOI:** 10.1002/advs.202413422

**Published:** 2024-12-04

**Authors:** Shuohui Dong, Haolin Cao, Ye Yuan, Shuo Liang, Zhendong Fu, Wei Shi, Qian Xu, Xiang Zhao, Jingnan Shi, Xiaoxiao Guo, Kaili Guo, Sanyuan Hu, Guangyong Zhang, Lizeng Gao, Lei Chen

**Affiliations:** ^1^ Department of General Surgery Qilu Hospital of Shandong University Jinan 250012 China; ^2^ CAS Engineering Laboratory for Nanozyme Key Laboratory of Biomacromolecules Institute of Biophysics Chinese Academy of Sciences Beijing 100101 China; ^3^ Nanozyme Medical Center School of Basic Medical Sciences Zhengzhou University Zhengzhou 450001 China; ^4^ Department of Otolaryngology‐Head and Neck Surgery Shandong Provincial ENT Hospital Shandong University Jinan 250022 China; ^5^ School of Life Sciences Jilin University Changchun 130012 China; ^6^ Department of General Surgery Shandong Provincial Qianfoshan Hospital The First Hospital Affiliated with Shandong First Medical University Jinan 250014 China; ^7^ Institute of Translational Medicine Department of Pharmacology School of Medicine Yangzhou University Yangzhou 225001 China

**Keywords:** cuproptosis, nanocatalytic therapy, nanozyme, reversal of chemoresistance, tumor therapy

## Abstract

Despite considerable advancements in the treatment of colorectal cancer (CRC), the overall survival rate for patients with advanced CRC remains below 50%, primarily due to challenges posed by drug resistance and metastasis. Here, a novel “Three‐in‐One” Cu‐based metal‐organic framework nanozyme with peroxidase‐like (POD‐like) activity has been successfully developed, aiming to promote CRC cell death by dual targeting of oxidative stress and copper ion homeostasis, which could promote CRC cell death via apoptosis and cuproptosis, and facilitate hypoxia‐inducible factor 1α (HIF‐1α) degradation, leading to the reversal of chemoresistance in tumor therapy. These nanozymes, composed of copper and 2‐propylimidazole (Cu‐PrIm), feature a distorted Cu‐N4 catalytic active center that mimics natural enzyme structures consisting of copper and histidine residues, endowing them with enzyme‐like activities. The antitumor efficacy of Cu‐PrIm nanozymes is validated in various in vivo models of CRC. Especially Cu‐PrIm nanozymes exhibit excellent biocompatibility, biodegradability, and a tolerable toxicity profile in mouse models, making them a strong candidate for clinical translation. Taken together, the study introduces a novel therapeutic paradigm in CRC treatment by targeting these vulnerabilities and leveraging the potential using “Three‐in‐One” Cu‐PrIm nanozymes to address multiple pathways simultaneously.

## Graphical abstract

In this study, a novel “Three‐in‐One” Cu‐PrIm nanozymes with peroxidase (POD)‐like activity has been developed, which could promote colorectal cancer (CRC) cell death via apoptosis and cuproptosis, and facilitate hypoxia‐inducible factor 1α (HIF‐1α) degradation leading to reversal of chemoresistance. This represents a promising treatment paradigm that leverages the potential of “Three‐in‐One” Cu‐PrIm nanozymes to simultaneously address multiple tumor‐specific vulnerabilities.

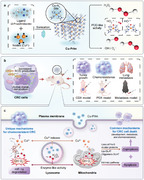

## 1 Introduction

Colorectal cancer (CRC) is a common form of malignancy and the primary contributor to global cancer‐related mortality.^[^ ^1^ ^]^ The modest advances achieved in CRC treatment during the era of precision medicine can be attributed to the ongoing refinement of first‐line chemotherapy regimens, advancements in immunotherapy and targeted therapy, and the systematic use of multiple pharmaceutical agents.^[^ ^1^, ^2^ ^]^ However, despite these advancements in treatment modalities, patients with advanced CRC still have poor outcomes.^[^ ^2^, ^3^ ^]^ Thus, it is imperative to develop new therapeutic paradigms to improve the efficacy in CRC treatment.

The reactive oxygen species (ROS) level in cancer cells acts as a double‐edged sword by serving as both a potent inducer of oncogenesis and facilitator of tumor progression, while also acting as a source of vulnerability that can be exploited in cancer treatment, and excessive ROS can induce oxidative damage to cellular components, such as proteins and nucleic acids, leading to cell death.^[^ ^4^ ^]^ Multiple studies have indicated that the initiation, progression, invasion, metastasis, and drug resistance of CRC are accompanied by the induction of oxidative stress and the accumulation of ROS, especially elevated hydrogen peroxide (H_2_O_2_).^[^ ^5^ ^]^ However, this increase alone is insufficient to induce cell death.^[^ ^6^ ^]^ Consequently, increasing intracellular ROS levels beyond a tolerable threshold to induce cancer cell death is a very effective treatment strategy in cancer therapy.^[^ ^4^, ^6^, ^7^ ^]^ Nanozymes are considered as superior candidates for targeting ROS levels in relation to proteins, natural small‐molecule compounds, or genetic manipulation, due to their high stability, low production costs, adjustable catalytic activity, and ease of modification.^[^ ^8^ ^]^ Despite limited by low catalytic activity and endogenous low concentrations of H_2_O_2_, the peroxidase‐like (POD‐like) nanozymes have demonstrated significant potential in promoting cancer cell death and inhibiting cancer cell proliferation by catalyzing the conversion of H_2_O_2_ into cytotoxic hydroxyl radicals (∙OH).^[^ ^8^, ^9^ ^]^

However, it is crucial to recognize that increased ROS levels may still be insufficient to induce cell death in certain scenarios, such as chemoresistance and metastasis.^[^ ^7c^ ^]^ This resistance is attributed to the enhanced antioxidant defense mechanisms and metabolic reprogramming in cancer cells.^[^ ^7c^ ^]^ Therefore, we utilized the design flexibility of nanozymes to introduce an additional killing mechanism—cuproptosis—synergizing with enzyme‐like activity to enhance therapeutic efficacy.^[^ ^10^ ^]^ Targeting metal homeostasis is a promising strategy for cancer therapy. The excessive metal ion accumulation can directly trigger cancer cell death and significantly impair cellular survival mechanisms.^[^ ^11^ ^]^ These perturbations can circumvent intrinsic resistance mechanisms in cancer cells, enhancing the efficacy of antitumor therapies.^[^ ^11^, ^12^ ^]^ The accumulation of excess Cu ions within the cell leads to the aggregation of lipoylated proteins and the destabilization of iron‐sulfur (Fe‐S) cluster proteins, ultimately resulting in cell death.^[^ ^12c^ ^]^ Various Cu ionophores, such as elesclomol, disulfiram, 8‐hydroxyquinoline, dithiocarbamates, and pyrithione, have been developed for this purpose.^[^ ^13^ ^]^ However, current Cu ionophores are typically small‐molecule compounds, rapidly eliminated from the bloodstream and inefficiently imported into cancer cells, this hinders Cu ions from accumulating at high concentrations needed to induce cuproptosis. In light of this, metal‐organic frameworks (MOFs), synthesized through the coordination of metal ions and organic ligands, emerge as promising candidates for antitumor therapy.^[^ ^14^ ^]^ MOFs offer advantages such as enhanced metal‐loading capacity and sustained ion release, along with commendable biocompatibility and biodegradability.^[^ ^15^ ^]^

**Figure 1:**
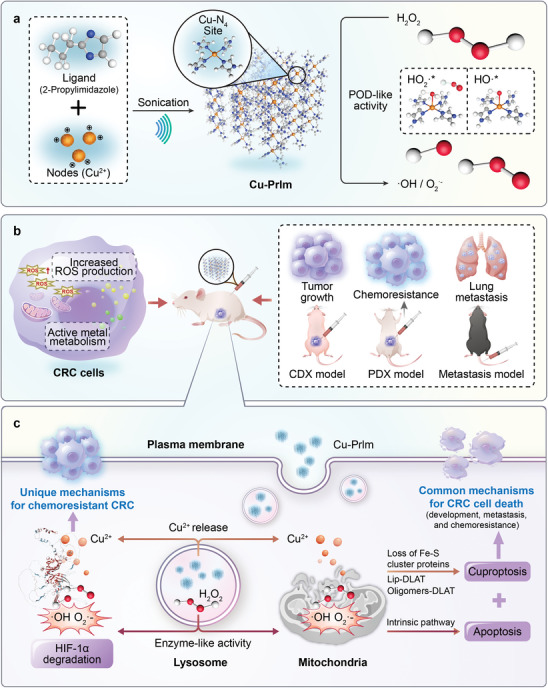
Schematic of Cu‐PrIm nanozymes for CRC treatment. a) Cu‐PrIm nanozymes were synthesized using a facile self‐assembly approach, facilitated by sonication. Distorted Cu‐N4 catalytic active center endows Cu‐PrIm with enzyme‐like activity to induce the production of ∙OH and ∙O_2_^−^ species. b) Cu‐PrIm nanozymes showed antitumor activity in various models of tumor growth, chemoresistance, and lung metastasis in CRC. c) Cu‐PrIm nanozymes induced both apoptosis and cuproptosis through its POD‐like activity and controlled release of copper ions.

Thus, this study aimed to utilize the architecture of MOF nanozymes to efficiently transport metals into cancer cells, facilitating sustained metal ion release during biodegradation in combination with its POD‐like catalytic activity to induce effective programmed cell death synergistically. In this work, we designed and synthesized a range of MOF nanozymes, M‐PrIm (M = Fe^2+^, Co^2+^, Ni^2+^, Cu^2+^, and Zn^2+^, PrIm = 2‐propylimidazole), that all exhibit POD‐like activity and serve as a kind of Trojan horse to transfer metal ions into cancer cells. Among them, Cu‐PrIm nanozymes demonstrated optimal performance in synergistically inducing programmed cell death in CRC cells (**Figure** **1**a). The coordination structure containing Cu and imidazole rings is the catalytic active center of many proteins and widely exists in organisms, referred to as the various structure consisting of copper and histidine residues, such as blue copper proteins, copper‐zinc superoxide dismutase, tyrosinase, laccases, etc.^[^ ^16^ ^]^ Cu‐PrIm nanostructures with a distorted Cu‐N4 catalytic active center and POD‐like activity can efficiently internalize into the lysosomes of CRC cells and induce overloaded ROS production (Figure 1c). The excessive ROS levels caused oxidative stress damage to mitochondria, leading to an imbalance in proapoptotic and antiapoptotic proteins. This imbalance increased the release of proapoptotic factors into the cytoplasm, ultimately initiating a fatal apoptotic cascade. Meanwhile, the combination of excessive ROS and Cu ion release during Cu‐PrIm biodegradation led to the aggregation of lipoylated proteins and destabilization of Fe‐S cluster proteins, ultimately causing cuproptosis. Additionally, Cu‐PrIm nanozymes could directly promote the degradation of hypoxia‐inducible factor 1α (HIF‐1α), a crucial protein involved in chemoresistance regulation,^[^ ^17^ ^]^ thereby enhancing its cytotoxic effect on refractory chemoresistant CRC cells. Thus, we showed that Cu‐PrIm nanozymes could successfully eradicate CRC and overcome chemoresistance through a “Three‐in‐One” strategy (Figure 1c). Finally, we validated the antitumor efficacy of Cu‐PrIm nanozymes in various mouse models of CRC, including the CRC‐cell‐line‐derived xenograft (CDX) model, the patient‐derived xenograft (PDX) model, the chemoresistance model, the drug‐tolerant persister (DTP) model, and the lung metastasis model (Figure 1b). Our research underscores the potential of MOF nanozymes with synergistic anticancer properties for cancer therapy.

## 2 Results and Discussion

### 2.1 Synthesis and Structural Analysis of Cu‐PrIm Nanozymes

**Figure 2:**
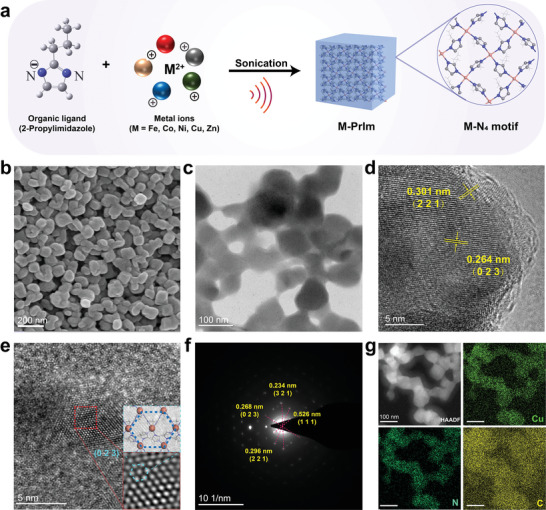
Synthesis and morphology characterization of Cu‐PrIm nanozymes. a) Schematic illustration of synthesis and structure of M‐PrIm nanozymes. b) SEM and c) TEM image of Cu‐PrIm nanozymes. d) HRTEM image of Cu‐PrIm nanozymes. e) FFT‐HRTEM image of Cu‐PrIm nanozymes and corresponding model of (023) crystalline plane. f) SAED patterns of Cu‐PrIm nanozymes. g) The EDX elemental mapping images of Cu‐PrIm nanozymes.

To concurrently achieve potent MOF‐based nanozymes, the M‐PrIm nanozymes (M = Fe^2+^, Co^2+^, Ni^2+^, Cu^2+^, and Zn^2+^) were synthesized using a facile self‐assembly approach, facilitated by sonication.^[^ ^18^ ^]^ The morphological analysis revealed that the M‐PrIm nanomaterials varied in size, ranging from 60 to 300 nm (Figure 2a; Figure S1, Supporting Information). As shown in Fourier Transform Infrared (FT‐IR) spectra, the typical peaks of M─N bond located at ≈400 cm^−1^ and characteristic peaks of C─H, N─H, and ring stretch of the PrIm, indicating that the metal ions successfully bind to the organic linkers (Figure S2, Supporting Information). Powder X‐ray diffraction (PXRD) characterization (Figure S3, Supporting Information) demonstrated that crystal MOF‐like structures have formed in M‐PrIm (M = Co^2+^, Cu^2+^, Zn^2+^). In addition, to facilitate the comparison of the effects of killing CRC cells by nanozymes based on copper and imidazole ligands substituted in the two‐position by alkyl chains with different lengths, we also synthesized a series of Cu‐L (L = MeIm, EtIm, BuIm) nanomaterials (Figure S4, Supporting Information). Then, the efficacy of M‐PrIm and Cu‐imidazole nanozymes in eradicating tumor cells was evaluated by assessing the viability of RKO cells following exposure to these nanomaterials. The results indicated that Cu‐PrIm nanozymes exhibited superior efficacy compared to both M‐PrIm and Cu‐imidazole nanomaterials (Figures S5 and S6, Supporting Information), warranting further investigation.

The Cu‐PrIm nanoparticles possessed an irregular cubic‐like shape with a size ≈60 nm (**Figure** **2**b,c), and the HRTEM images revealed clear lattice patterns with interplanar distances of 0.264 and 0.301 nm (Figure 2d), which were matched well to the (023) and (221) facets of models we have constructed (Figure S7, Supporting Information). Fast Fourier transform (FFT) was utilized to obtain more details from the HRTEM images, indicating the single copper atoms on the surface of (023) (Figure 2e). Moreover, the selected area electron diffraction (SAED) patterns further clarified more details of crystalline structure of Cu‐PrIm nanoparticles, involving (111) and (321) crystal facets (Figure 2f). The energy‐dispersive X‐ray (EDX) elemental mapping analysis indicated that Cu, C, and N elements were homogeneously distributed in Cu‐PrIm nanozymes (Figure 2g).

**Figure 3:**
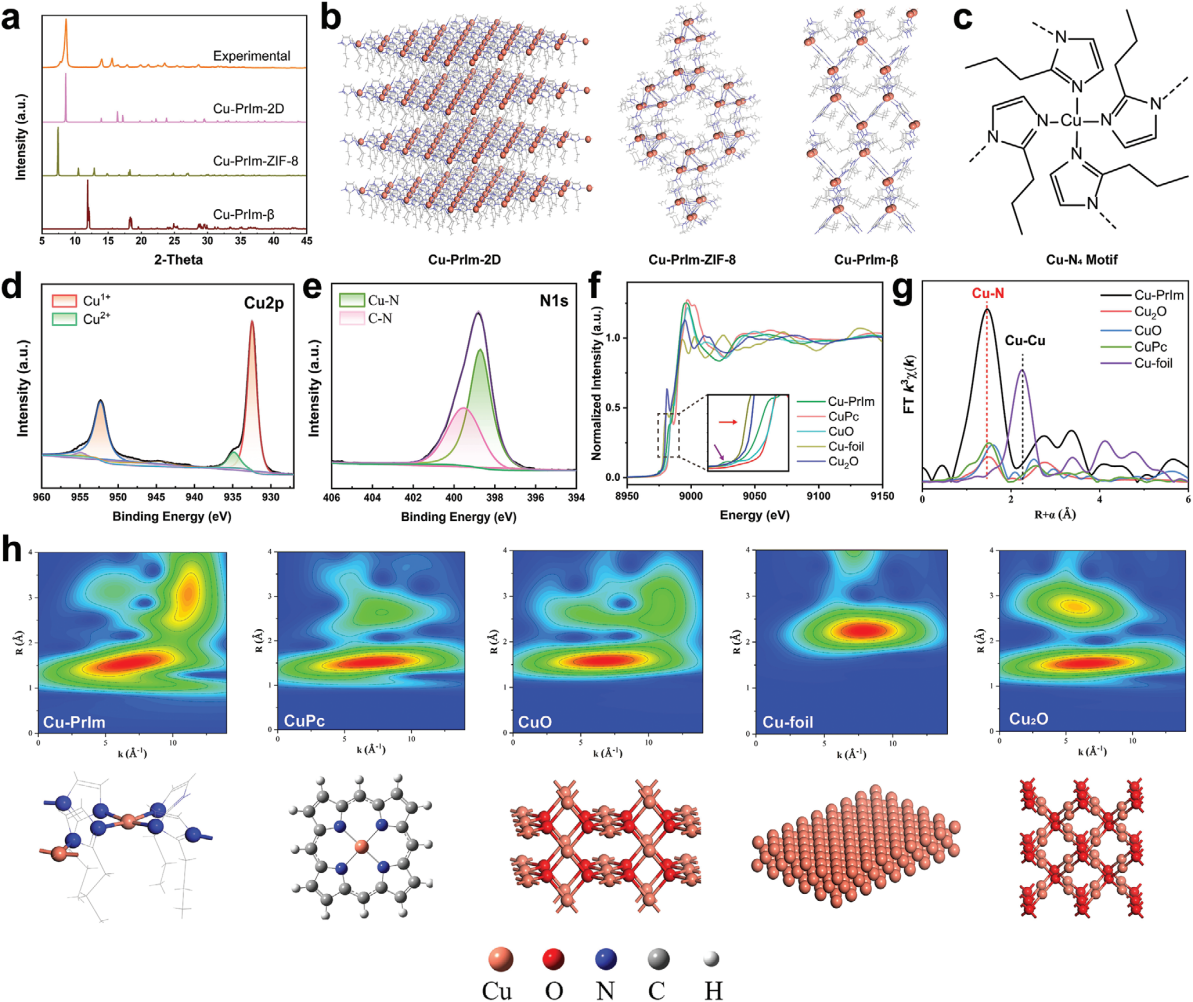
Atomic structure characterization of Cu‐PrIm nanozymes. a) PXRD patterns of Cu‐PrIm nanozymes and simulations of Cu‐PrIm‐2D, Cu‐PrIm‐ZIF‐8, and Cu‐PrIm‐β. b) Structure models of Cu‐PrIm‐2D, Cu‐PrIm‐ZIF‐8 and Cu‐PrIm‐β from left to right. c) Structure diagram of proposed central Cu‐N_4_ motif in Cu‐PrIm nanozymes. d) Cu2p and e) N1s XPS spectra of Cu‐PrIm nanozymes. f) XANES spectra of Cu K‐edge for Cu‐PrIm nanozymes with reference materials Cu‐foil, CuO, Cu_2_O, and CuPc. g) k^3^‐weighted FT‐EXAFS spectra of the experimental Cu K‐edge EXAFS signal of Cu‐PrIm nanozymes along with reference samples. h) WT of Cu‐PrIm, CuPc, CuO, Cu‐foil, and Cu_2_O and corresponding structure models.

To elucidate the precise structural configuration of Cu‐PrIm nanozymes, detailed PXRD analysis was performed in conjunction with periodic density functional theory (DFT) simulations (**Figure** **3**a). Three typical models involving 2D structure of square lattice topology (Cu‐PrIm‐2D), ZIF‐8‐based structure (Cu‐PrIm‐ZIF‐8), and monoclinic β‐phase (Cu‐PrIm‐β) were built according to the spatial expanding directions (Figure 3b), and their corresponding PXRD patterns were subsequently simulated. The Cu‐PrIm‐2D and Cu‐PrIm‐β topologies both have planar Cu‐N4 coordination geometries due to the Jahn–Teller effect resulting from the d^9^ electronic configuration in copper(II). Clear diffraction peaks in the experimental PXRD result were located at 8.67, 13.98, and 15.54°, which matched the simulated pattern of Cu‐PrIm‐2D the best. Moreover, peaks of Cu, C, and N elements could be clearly observed in the full spectrum of X‐ray photoelectron spectroscopy (XPS) survey (Figure S8, Supporting Information), which was consistent with EDX mapping (Figure 2g). In the narrow scan of Cu2p (Figure 3d), the characteristic peaks at 952.3 and 932.3 eV are attributed to Cu^1+^, and peaks of Cu^2+^ were also observed at 935.0 and 954.8 eV. The ratio of Cu^1+^ and Cu^2+^ was ≈15:1, indicating most Cu in Cu‐PrIm was in an electron‐rich environment due to the PrIm ligand as a strong electron doner.^[^ ^19^ ^]^ The 1s spectra of both C and N elements further demonstrated the coordination between Cu and PrIm (Figure 3e; Figure S9, Supporting Information). The corresponding atomic ratio of Cu:N in Cu‐PrIm was close to 1:4 was further analyzed by an Inductively coupled plasma optical emission spectrometer (ICP‐OES) and an organic element analyzer (OEA) (Figure S10, Supporting Information), confirming the Cu‐N_4_ structure.

The precise structure of Cu‐PrIm was further elucidated by X‐ray absorption fine structure (XAFS) spectroscopy and K‐edge extended X‐ray absorption fine structure (EXAFS). The pre‐edge position for Cu‐PrIm resided between the positions of CuPc (CuO) and CuO_2_ (the black line area) demonstrates a lower oxidation state between +1 and +2 (red arrow, Figure 3f), and the peak at 8975 eV which represented the electron transfer from the ligand to central Cu suggested that the Cu‐N_4_ motif was contained in Cu‐PrIm (purple arrow).^[^ ^20^ ^]^ Coordinating state of central metal Cu was then studied by K^3^‐weighted Fourier‐Transformed‐EXAFS (FT‐EXAFS) (Figure 3g). The predominant peaks at 1.48 Å could be attributed to the scattering of Cu─N bond, which was consistent with the results of PXRD and XPS. The fitted EXAFS curves in R and K space indicated that the distance of Cu─N bond was 1.95 Å (Figure S11, Supporting Information), which was close to Cu─N bond length (2.01 Å) in Cu‐PrIm‐2D model. Besides, the wavelet transform (WT) analysis (Figure 3h) showed that the signal of Cu‐PrIm confirmed the formation of Cu‐N_4_ coordination in Cu‐PrIm (Figure 3c).

### 2.2 POD‐Like Activity and GSHOx Ability of Cu‐PrIm Nanozymes

**Figure 4:**
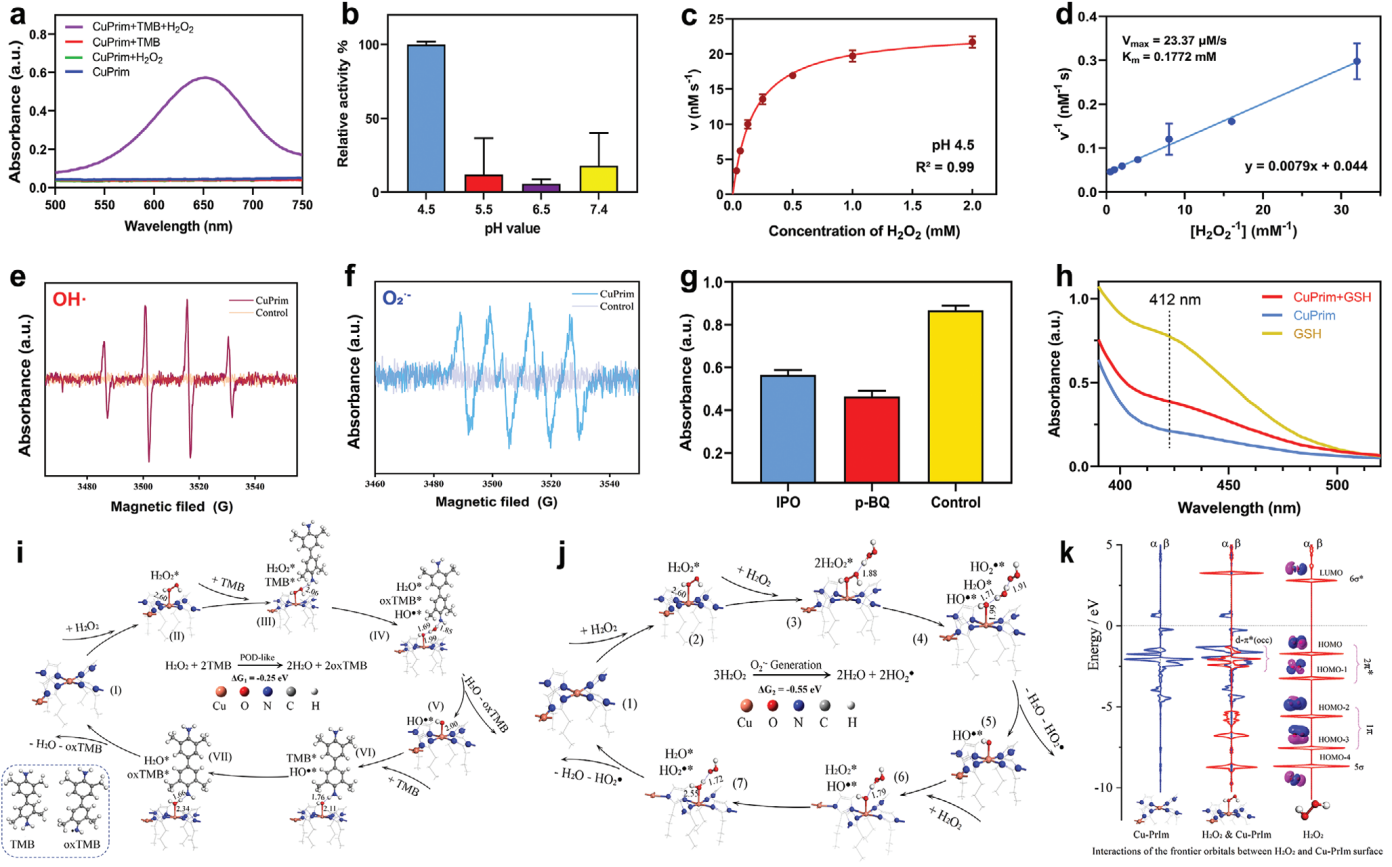
POD‐like activity and GSHOx ability of Cu‐PrIm nanozymes. a) UV–vis absorbance spectra of TMB in a pH 4.5 HAc−NaAc buffer after 10 min of incubation in different reaction systems involving (1) Cu‐PrIm + TMB + H_2_O_2_, (2) Cu‐PrIm + TMB, (3) Cu‐PrIm + H_2_O_2_ and (4) Cu‐PrIm. b) pH‐dependent POD‐like activity of Cu‐PrIm nanozymes. c) Michaelis–Menten kinetic analysis and d) Lineweaver–Burk plot for Cu‐PrIm nanozymes with H_2_O_2_ as a substrate. e,f) ESR singnals of e) DMPO/·OH and f) DMPO/∙O_2_^−^ for the Cu‐PrIm (200 µg mL^−1^) reacting with H_2_O_2_ (20 mm) for 20 min in a pH 4.5 HAc−NaAc buffer. g) ROS quenching assay with IPO (∙OH) and p‐BQ (∙O_2_^−^) as quenching agents. h) GSH consumption after incubated with Cu‐PrIm nanozymes for 30 min. i) Proposed reaction pathway to achieve the POD‐like catalytic cycle on Cu‐PrIm nanozymes. j) Proposed reaction pathway to achieve the catalytic cycle from H_2_O_2_ to HO_2_∙ on Cu‐PrIm nanozymes. k) Interactions of the frontier orbitals between H_2_O_2_ and Cu‐PrIm surface.

POD‐like activity of Cu‐PrIm nanozymes was evaluated by a colorimetric method with 3,3′,5,5′‐tetramethylbenzidine dihydrochloride (TMB) as the probe. Toxic hydroxyl radicals and other ROS would be generated when the Cu‐PrIm was induced by H_2_O_2_, and the TMB was oxidized into oxTMB with a characteristic absorption at 652 nm (**Figure** **4**a). The absorbance degree of oxTMB exhibited an elevated trend with the increasing of reaction time and Cu‐PrIm concentration (Figure S12, Supporting Information). Moreover, the POD‐like activity of M‐PrIm (M = Fe^2+^, Co^2+^, Ni^2+^, Zn^2+^) and Cu‐L (L = MeIm, EtIm, BuIm) were also listed in Figures S13 and S14 (Supporting Information), which indicated that the catalytic activity of all nanozymes were essentially equivalent. The POD‐like activity of Cu‐PrIm nanozymes was then evaluated in NaAc‐HAc buffer with different pH values (Figure 4b). Notably, Cu‐PrIm nanozymes displayed a pH‐dependent POD‐like activity and the optimal catalytic condition was pH 4.5, which was close to that in the microenvironment of lysosomes in tumor cells. In weakly acidic and neutral environments, however, Cu‐PrIm nanozymes hardly generate ROS, demonstrating the excellent biosafety in normal physiological environment. In addition, the Michaelis–Menton kinetics curves of Cu‐PrIm nanozymes were evaluated by fixing the concentration of Cu‐PrIm and TMB while changing the concentration of H_2_O_2_ (Figure 4c,d). The Michaelis–Menten constant (K_m_) and a maximal reaction velocity (V_max_) were calculated to be 0.18 mM and 23.37 × 10^−6^ M s^−1^, indicating the high ROS generating ability under a low concentration of H_2_O_2_. The specific types of ROS generated by Cu‐PrIm nanozymes in the presence of H_2_O_2_ was further measured by electron spin resonance (ESR) spectroscopy, where 5,5‐dimethyl‐1‐pyrroline *N*‐oxide (DMPO) was employed as the radical trap reagent in different solvent. Characteristic signals of ∙O_2_^−^ and ∙OH could be observed in the ESR spectra, indicating the generation of multiple types of ROS (Figure 4e,f). Free radical quenching experiment was further conducted to verify the existence of both radicals (Figure 4g).

Glutathione oxidase (GSHOx) activity of Cu‐PrIm nanozymes was able to deplete reduced glutathione (GSH) in tumor cells, which would facilitate the damage effect of ROS in vivo. The GSHOx activity of Cu‐PrIm nanozymes was evaluated using 5,5'‐Dithiobis‐(2‐nitrobenzoic acid) (DTNB) as a probe, which could react with free thiol group of GSH and form yellow complex TNB with a characteristic absorbance at 412 nm. An obvious decrease of absorbance at 412 nm was observed when Cu‐PrIm was incubated with DTNB in PBS solution, verifying the GSHOx activity of Cu‐PrIm nanozymes (Figure 4h). Similar to the POD‐like activity, the GSHOx activity of Cu‐PrIm nanozymes also followed Michaelis–Menton kinetics, and the corresponding K_m_ and V_max_ were calculated to be 0.39 mm and 0.336 µm s^−1^ (Figure S15, Supporting Information). In this process, by product H_2_O_2_ would be generated after the GSH oxidized by oxygen, which was further detected by the specific probe Amplex red (AR). As shown in Figure S16 (Supporting Information), the generation of H_2_O_2_ was proved by the increase of absorbance at 570 nm. Collectively, the POD and GSHOx activity of Cu‐PrIm nanozymes could form a self‐cascade system (GSHOx‐POD) in the microenvironment of tumor lysosomes, efficiently generate toxic ROS and destroy the intracellular redox homeostasis.

To better elucidate the catalytic mechanisms, we simulated the POD‐like activity and the catalytic process for generating superoxide anions by H_2_O_2_ molecules on Cu‐PrIm nanozymes, and the corresponding proposed reaction pathways were investigated using density functional theory (DFT) calculations as shown in Figure 4i–k. As is known that the O_2_^∙−^ is a Brønsted base with pKb = 9.12, and it can easily capture a proton from water to form HO_2_^∙^ and OH^−^,^[^ ^21^ ^]^ as illustrated by the following equation:

(1) $${O_{2}}^{•-}+H_{2}O\to H{O_{2}}^{•}+OH^{-}$$

Therefore, the following Equations (2) and (3) serve as plausible mechanisms for the POD‐like activity and the superoxide anion generation, respectively:

(2) $$H_{2}O_{2}+2\mathrm{TMB}\to 2H_{2}O+2\mathrm{oxTMB}$$

(3) $$3H_{2}O_{2}\to 2H_{2}O+2H{O_{2}}^{•}$$

The whole POD‐like catalytic cycle (Equation 2) consists of seven steps as described in Figure 4i, and two TMB molecules can be oxidized by a H_2_O_2_ molecule converting to two oxTMB molecules on Cu‐PrIm nanozymes. The corresponding change in Gibbs free energy ΔG_1_ is −0.25 eV (Figure 4i; Figure S18, Supporting Information), which indicated the POD‐like catalytic cycle could proceed spontaneously. Figure 4j showed that three H_2_O_2_ molecules undergone disproportionation reactions generating two H_2_O molecules and two HO_2_^∙^ free radicals on Cu‐PrIm nanozymes, and this catalytic process was easily to occur indicated by the corresponding change in Gibbs free energy (ΔG_2_ = −0.55 eV in Figure 4j; Figure S19, Supporting Information). To elucidate the binding nature of the species involved in the mechanism, we calculated the densities of states (DOS) of the H_2_O_2_ molecule, Cu‐PrIm MOF, and their interaction configuration (Figure 4k). The electronic structure analysis indicates that the energy levels of Cu‐PrIm MOF and H_2_O_2_ orbitals are well matched in the range of −5.0 to −0.0 eV below Fermi level, leading to partial occupation of the formed d‐π^*^ orbitals, which was clearly described by the frontier orbitals. These frontier orbitals show that the interaction between H_2_O_2_ and Cu‐PrIm MOF mainly comes from the chemical bonds between 3d electron of Cu and 2π^*^ electron of H_2_O_2_. These chemical bonds can effectively weaken the bonding effect inside the H_2_O_2_ and are responsible for the activation of the substrate. Free radical capture experiments were performed to investigate the generation of ∙O_2_^−^ and ∙OH free radicals under different conditions (Figure S20, Supporting Information). The results showed that temperature, H_2_O_2_ concentration, and proton concentration all could regulate the production of ∙OH and ∙O_2_^−^ free radicals.

### 2.3 The In Vitro POD‐Like Activity and Cytotoxicity of Cu‐PrIm Nanozymes

The initiation, progression, metastasis, and acquisition of drug resistance in CRC are linked to oxidative stress, which is characterized by an imbalance in free radical and antioxidant levels.^[^ ^4^, ^22^ ^]^ Recent research suggests that exacerbating oxidative stress within cancer cells is a promising strategy for boosting the antitumor efficacy of existing anticancer drugs and combatting drug resistance.^[^ ^17^, ^23^ ^]^ The findings presented here illustrate the ability of metal‐based nanozymes containing the PrIm group to exhibit POD‐like activity, prompting further investigation into their in vitro antitumor effects on CRC cells. The physiochemical properties and biological functions of metal‐based PrIm nanozymes are largely dictated by the metal ion species contained within.^[^ ^24^ ^]^ Thus, we first compared the enzyme‐like activities and antitumor effects of PrIm nanozymes containing different metal ions.

**Figure 5:**
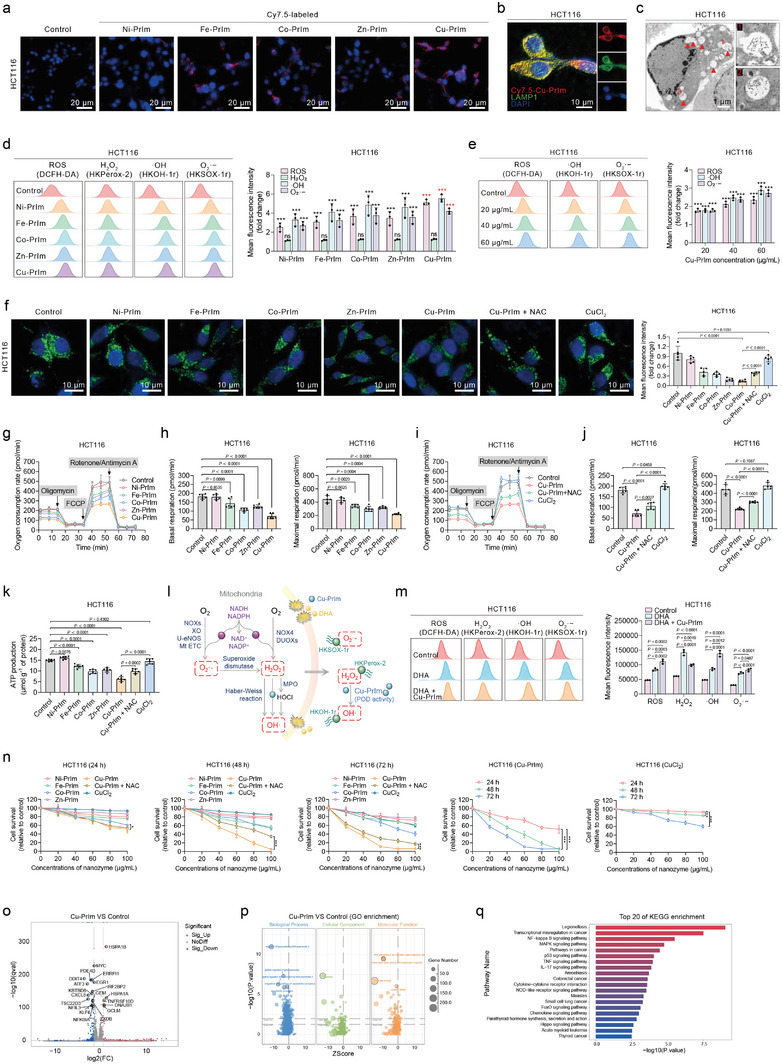
In vitro POD‐like activity and cytotoxicity of Cu‐PrIm nanozymes. a) Intracellular uptake of Cy7.5‐labeled nanozymes (red). Scale bar = 20 µm. b) Co‐localization of Cy7.5‐labeled Cu‐PrIm (red) and the lysosomal marker LAMP1 (green). Scale bar = 10 µm. c) Representative TEM images of HCT116 cells after Cu‐PrIm treatment. Scale bar = 1 µm. d) Levels of total ROS and specific ROS species in HCT116 cells after exposure to nanozymes. e) Levels of total ROS and specific ROS species in HCT116 cells. f) Representative fluorescence images and statistical graph of MitoTracker Green probe (green). Scale bar = 10 µm. g) OCR after pretreatment with indicated nanozymes. h) Basal and maximal respiration. i) OCR after pretreatment with Cu‐PrIm or CuCl_2_. j) Basal and maximal respiration. k) ATP production in HCT116 cells after indicated treatment. l) Schematic diagram of intracellular ROS generation and the interconversion of different kinds of ROS. m) Levels of total ROS and specific ROS species in HCT116 cells after successive exposure to DHA and Cu‐PrIm. n) Viability of HCT116 cells after different treatments with various concentrations and times. o) Volcano plot was drawn to show the differentially expressed genes in Cu‐PrIm treated HCT116 cells versus control. p) GO term enrichment analysis of up‐ and down‐regulated genes. q) The top 20 KEGG pathways by *p‐*value. For all studies, *n* ≥ 3. Data are shown as the mean ± SD. Comparisons were performed using the Student's *t*‐test or ANOVA. *p < 0.05, ***p < 0.001, or ns = not significant.

We first evaluated the ability of the CRC cell lines, HCT116 and RKO, to endocytose the metal‐based PrIm nanozymes. The findings indicated the Cu‐PrIm nanomaterials showed the highest level of intracellular accumulation (**Figure** **5**a; Figure S21, Supporting Information). The fluorescence colocalization and transmission electron microscopy (TEM) analyses indicated that the Cu‐PrIm nanozymes were localized within the lysosomes (Figure 5b,c; Figures S22 and S23, Supporting Information). This finding aligns with the notion that the acidic environment within lysosomes optimizes the function of metal‐based PrIm nanomaterials. The fluorescence probes enabled us to observe that the ROS levels in CRC cells significantly increased after exposure to the M‐PrIm nanozymes, and the results indicated that the Cu‐PrIm nanozymes exhibited the most significant catalytic activity (Figure 5d,e; Figures S24 and S25, Supporting Information).

Given that mitochondria are the primary regulators of oxidative stress and are particularly vulnerable to ROS‐mediated damage,^[^ ^25^ ^]^ we next conducted a thorough examination of any structural and functional abnormalities induced by nanozymes in mitochondria. Visualization of mitochondria with the MitoTracker Green probe indicated that, among all the treated CRC cells, those treated with Cu‐PrIm nanozymes had the lowest MitoTracker Green, suggestive of significant mitochondrial damage (Figure 5f; Figure S26, Supporting Information). To further evaluate the impact of M‐PrIm nanozymes on mitochondrial respiration, the oxygen consumption rate (OCR) of the CRC cells exposed to the different experimental conditions were measured and the results indicated that the Cu‐PrIm nanozymes showed the strongest ability to induce mitochondrial dysfunction (Figure 5g,h; Figure S27, Supporting Information). Impaired mitochondrial respiration may result in diminished ATP production. Subsequent experiments revealed a more significant reduction in intracellular ATP concentrations in CRC cells following exposure to Cu‐PrIm nanozymes than others (Figure 5k; Figure S28, Supporting Information), while CuCl_2_ did not alter mitochondrial architecture or impair mitochondrial function in CRC cells. Additionally, we observed that high concentrations of N‐acetylcysteine (NAC) could reverse Cu‐PrIm‐induced mitochondrial damage (Figure 5f,i–k; Figures S26–S28, Supporting Information). These results suggest that the mitochondrial damage caused by M‐PrIm nanozymes is closely linked to their POD‐like activity.

The above results revealed that Cu‐PrIm nanozymes could induce oxidative stress and mitochondrial dysfunction. To elucidate the impact of Cu‐PrIm nanozymes on the interconversion of different kinds of ROS, we treated CRC cells with high concentrations of docosahexaenoic acid (DHA) to induce mitochondrial damage and ROS generation, before subjecting them to the Cu‐PrIm nanomaterials, and the results indicated that Cu‐PrIm nanozymes could further increase the total level of ROS in the DHA‐treated CRC cells, particularly by increasing the levels of ∙OH and ∙O_2_^−^, while decreasing those of H_2_O_2_ (Figure 5l,m).

Excessive ROS generation and mitochondrial damage have the potential to trigger programmed cell death.^[^ ^26^ ^]^ A series of experiments were carried out to assess the cytotoxic effects of M‐PrIm nanozymes. The results of the CCK‐8 assay suggested that the Cu‐PrIm nanozymes showed the highest levels of cytotoxicity among all nanozymes at equivalent concentrations (Figure 5n; Figure S29, Supporting Information), and the CRC cells treated with Cu‐PrIm nanozymes underwent time‐dependent cell death, whereas no significant time‐dependent inhibition was observed for the other M‐PrIm nanomaterials. Particularly, Cu‐PrIm nanozymes showed cytotoxicity against various types of cancer, including CRC, lung cancer, breast cancer, liver cancer, and melanoma (Figure S30, Supporting Information). To gain deeper insights into the molecular alterations associated with Cu‐PrIm nanozymes treatment, we next performed a transcriptomic analysis. Transcriptional profiling of Cu‐PrIm‐treated CRC cells revealed that the nanozyme induced significant changes in the expression of 326 genes; 119 were upregulated (log_2_FC ≥ 1, q < 0.05) and 207 down regulated (log_2_FC ≤ −1, q < 0.05) (Figure 5o; Figure S31, Supporting Information). Moreover, the transcriptomic data showed that Cu‐PrIm nanozymes induced the activation of signaling pathways associated with cell death and engaged multiple molecular pathways (Figure 5p,q; Figure S31, Supporting Information).

Cu‐PrIm exhibited the strongest ability for oxidative stress disruption and antitumor efficacy compared to other M‐PrIm nanomaterials. We also proceeded to analyze the intracellular accumulation, ROS formation, and cytotoxicity of Cu‐MeIm, Cu‐EtIm, Cu‐PrIm, and Cu‐BuIm to identify the most effective Cu‐based nanomaterials for combating CRC cells (Figure S32, Supporting Information). Results indicated that the Cu‐PrIm nanozymes exhibited the best performance, although these Cu‐based nanomaterials could induce both ROS formation and cytotoxicity in CRC cells.

### 2.4 Mechanisms Underlying the Induction of Apoptosis by Cu‐PrIm Nanozymes

**Figure 6:**
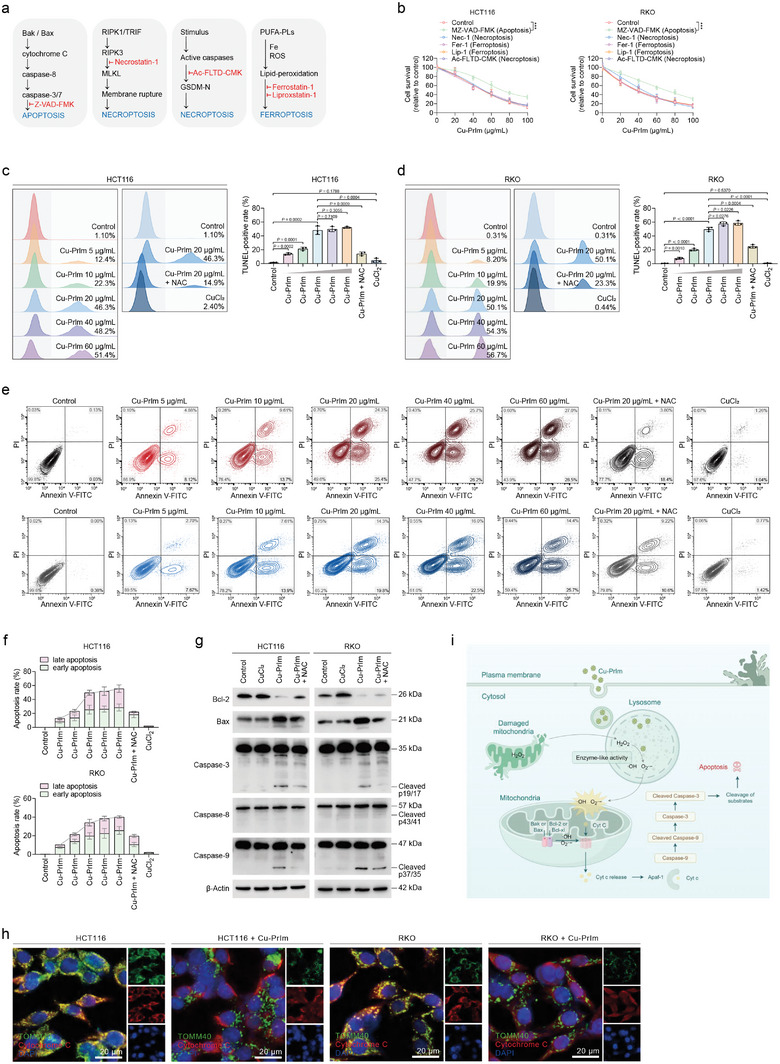
Apoptosis caused by Cu‐PrIm nanozymes. a) Schematic diagram of apoptosis, necroptosis, pyroptosis, and ferroptosis. Inhibited pathways are marked in red. b) Viability of CRC cells treated with gradient concentrations of Cu‐PrIm and 30 µm Z‐VAD‐FMK, 20 µm necrostatin‐1, 10 µm ferrostatin‐1, 10 µm liproxstatin‐1, or 10 µm Ac‐FLTD‐CMK for 24 h. c,d) Rates of TUNEL‐positive (apoptotic) CRC cells after the indicated treatments for 24 h. e,f) Ratios of early apoptotic (Annexin V‐FITC^+^/PI^−^) and late apoptotic (Annexin V‐FITC^+^/PI^+^) CRC cells after the indicated treatments for 24 h. g) Immunoblot of apoptotic proteins in CRC cells. h) Representative fluorescence images of HCT116 cells showing co‐localization of TOMM20 (green) and cytochrome C (red). Scale bar = 10 µm. i) Mechanism diagram of apoptosis induced by Cu‐PrIm nanozymes. For all studies, *n* ≥ 3. Data are shown as the mean ± SD. Comparisons were performed using the Student's *t*‐test or ANOVA. ***p < 0.001. β‐Actin was used as the internal reference for immunoblot.

Although the process of cell death encompasses complex molecular mechanisms and signaling pathways, researchers have concentrated on the four main types of programmed cell death: apoptosis, necroptosis, pyroptosis, and ferroptosis (**Figure** **6**a).^[^ ^27^ ^]^ Using a combination of Cu‐PrIm nanozymes and the necroptosis inhibitor necrostatin‐1 in CRC cells revealed that the mechanism of Cu‐PrIm‐induced cell death differed from that of necroptosis (Figure 6b). The administration of the ferroptosis inhibitors ferrostatin‐1 or liproxstatin‐1 did not reverse Cu‐PrIm‐induced cell death (Figure 6b). Likewise, the use of Ac‐FLTD‐CMK to inhibit pyroptosis via gasdermin D (GSDMD) did not reverse Cu‐PrIm‐induced cell death (Figure 6b). It is important to mention that the inhibition of apoptosis by the pan‐caspase inhibitor Z‐VAD‐FMK was able to partially reverse the cell death induced by Cu‐PrIm nanozymes (Figure 6b). These findings suggest that Cu‐PrIm‐induced CRC cell death is mechanistically dependent on apoptosis but not necroptosis or ferroptosis.

To support these findings, we performed a quantitative analysis of Cu‐PrIm‐induced apoptosis. The results of the TUNEL assay demonstrated a notable increase in the number of TUNEL‐positive apoptotic CRC cells following exposure to Cu‐PrIm nanozymes in a dose‐dependent manner (Figure 6c,d). Moreover, Annexin V‐FITC/PI staining confirmed that Cu‐PrIm nanozymes induced both early and late apoptosis in CRC cells (Figure 6e,f). Of note, CRC cells treated with 20 µg mL^−1^ concentration approached the maximum threshold for apoptosis induction (Figure 6c–f).

The critical regulatory proteins in Cu‐PrIm‐treated CRC cells were characterized to further understand the molecular mechanism of Cu‐PrIm‐induced apoptosis. The expression of apoptosis‐regulating proteins, B cell lymphoma gene 2 (Bcl‐2) and Bcl‐2‐associated X (Bax), in CRC cells was notably altered following exposure to Cu‐PrIm nanozymes; specifically, a significant decrease in the levels of the antiapoptotic protein Bcl‐2 and an increase in those of the proapoptotic protein Bax was observed (Figure 6g). The activation of caspase‐3, a key mediator of apoptosis, was also observed in CRC cells treated with Cu‐PrIm nanozymes. Previous research has shown that the dysregulated expression of Bcl‐2 and Bax can lead to the release of cytochrome C from mitochondria; the release of cytochrome C represents a critical step in apoptosis initiation.^[^ ^28^ ^]^ Immunofluorescence analysis revealed that Cu‐PrIm nanozymes induced cytochrome C release in CRC cells, as evidenced by the colocalization of cytochrome C and mitochondria in the images (Figure 6h; Figure S33, Supporting Information). Apoptosis is categorized into extrinsic and intrinsic pathways. Intrinsic apoptosis is triggered by cellular changes such as DNA and mitochondrial damage. Meanwhile, extrinsic apoptosis is initiated by the activation of death receptors located on the cytoplasmic membrane.^[^ ^29^ ^]^ Western blotting revealed significant activation of caspase‐9, a key player in intrinsic apoptosis, in Cu‐PrIm‐treated CRC cells, while caspase‐8, which is associated with extrinsic apoptosis, remained uncleaved (Figure 6g). We noted that CuCl_2_ did not elicit apoptosis in CRC cells. Furthermore, the antioxidant NAC successfully mitigated Cu‐PrIm‐induced apoptosis in CRC cells, as evidenced by both the phenotypic and molecular features of the treated cells (Figure 6c–g). These experimental results indicated that the POD‐like activity of Cu‐PrIm nanozymes induced oxidative stress damage in mitochondria, leading to the dysregulation of Bcl‐2 and Bax levels. A change in the Bcl‐2 to Bax ratio, in turn, caused mitochondrial outer membrane permeabilization and the release of apoptotic factors into the cytoplasm. The subsequent release of cytochrome C from mitochondria into the cytoplasm promoted the assembly of the apoptosome complex, which facilitated the activation of caspase‐9. Subsequently, caspase‐3, which is proteolytically activated by caspase‐9, carried out further protein cleavage events that eventually lead to apoptotic cell demise (Figure 6i).

### 2.5 Cu‐PrIm Nanozymes Trigger Cuproptosis to Exacerbate Cytotoxicity

**Figure 7:**
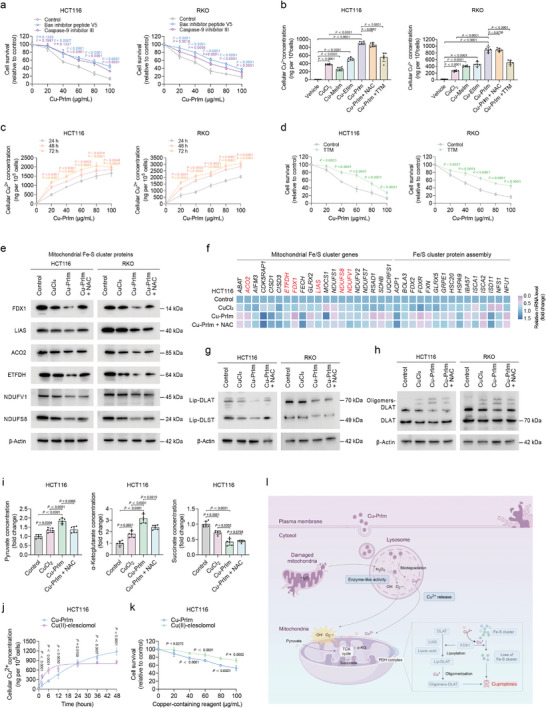
Cuproptosis caused by Cu‐PrIm nanozymes. a) Viability of CRC cells treated with a gradient concentration of Cu‐PrIm and indicated inhibitors. b) Copper ion concentrations of CRC cells after treatment with indicated agents. c) Copper ion concentrations of CRC cells after Cu‐PrIm nanozymes treatment. d) Viability of CRC cells treated with a gradient concentration of Cu‐PrIm and control or 20 µm TTM. e) Immunoblot of mitochondrial Fe‐S cluster proteins in CRC cells. f) PCR analysis of mitochondrial Fe/S cluster genes and Fe/S cluster protein assembly in HCT116 cells. g) Immunoblot analysis of lipoylation of DLAT and DLST in CRC cells after the indicated treatments. h) Immunoblot analysis of oligomerization of DLAT in CRC cells after the indicated treatments. i) Pyruvate, α‐ketoglutarate, and succinate concentrations in HCT116 cells. j) Copper ion concentrations in HCT116 cells at different time points after treatment with 40 µg mL^−1^ Cu‐PrIm or Cu(II)‐elesclomol. k) Viability of HCT116 cells after treatment with Cu‐PrIm or Cu(II)‐elesclomol at various concentrations for 24 h. l) Mechanism diagram of cuproptosis induced by Cu‐PrIm nanozymes. For all studies, *n* ≥ 3. Data are shown as the mean ± SD. Comparisons were performed using the Student's *t*‐test or ANOVA. β‐Actin (*ATCB*) was used as the internal reference for immunoblot and PCR.

Although the Cu‐PrIm nanozymes triggered apoptosis in CRC cells, the pan‐caspase inhibitor did not fully protect them from cell death, suggesting that an additional cell death mechanism was involved (Figure 6b). We therefore conducted further experiments to investigate the ability of other apoptotic inhibitors with distinct mechanisms to reverse Cu‐PrIm‐induced cell death. Although both the Bax inhibitor peptide V5 and caspase‐9 inhibitor III partially reversed Cu‐PrIm‐induced cell death, neither was able to completely prevent cell death (**Figure** **7**a). Our earlier data showed that although the administration of Cu‐PrIm nanozymes at a concentration of 20 µg mL^−1^ induced a near‐maximal rate of apoptosis in CRC cells (Figure 6c–f), escalating the dose induced further CRC cell death (Figure 5n; Figure S29, Supporting Information). The mitochondria of Cu‐PrIm‐treated cells contained large vacuoles, not a typical ultrastructural characteristic associated with apoptosis (Figure S34, Supporting Information). These findings indicate that Cu‐PrIm nanomaterials induce an additional death mechanism in CRC cells, which is district from that of apoptosis.

As shown in Figure S17 (Supporting Information), Cu‐based nanozymes exhibited the capability to effectively release Cu^2^⁺, thereby serving as potential copper ionophores. Among the various Cu‐based nanomaterials, Cu‐PrIm nanozymes demonstrated a markedly higher release of Cu^2^⁺ ions under acidic conditions. Cu‐PrIm nanozymes released the highest levels of Cu ions within CRC cells (Figure 7b), and the Cu ion release from Cu‐PrIm nanozymes into CRC cells was time‐ and dose‐dependent (Figure 7c). Notably, although NAC could not suppress Cu ion release from Cu‐PrIm nanozymes, the administration of the Cu chelator tetrathiomolybdate (TTM) partially inhibited Cu ion release and mitigated Cu‐PrIm‐induced cell death (Figure 7b,d). Meanwhile, treating CRC cells with CuCl_2_ did not increase their levels of ROS production while resulting in higher intracellular Cu ion concentrations (Figure 7b), which was potentially linked with the mechanism of CuCl_2_‐induced cell death (Figure 5n; Figure S29, Supporting Information). These findings prompted us to establish a correlation between the Cu‐PrIm‐induced cell death and cuproptosis.

We next investigated whether cuproptosis was triggered by Cu‐PrIm nanozymes at the molecular level. The hallmarks of cuproptosis, including a decrease in Fe‐S cluster protein levels and mitochondrial enzyme lipoylation, were identified in CRC cells following Cu‐PrIm nanozymes treatment. Specifically, the expression of multiple mitochondrial Fe‐S cluster proteins, including FDX1, LIAS, ACO2, ETFDH, NDUFV1, and NDUFS8, decreased in CRC cells following Cu‐PrIm nanozymes treatment (Figure 7e). RT‐PCR was used to assess the alterations in the mRNA levels of mitochondrial Fe‐S cluster genes in Cu‐PrIm‐treated CRC cells. Consistently, genes implicated in both cuproptosis and mitochondrial respiration were significantly downregulated in CRC cells after Cu‐PrIm nanozymes treatment (Figure 7f; Figure S35, Supporting Information). DLAT and DLST are the structural components of the pyruvate dehydrogenase (PDH) and alpha‐ketoglutarate dehydrogenase (KDH) complexes, respectively.^[^ ^12c^ ^]^ The lipoylation and oligomerization of DLAT and DLST are characteristic features of cuproptosis.^[^ ^30^ ^]^ Our study revealed a reduction in the rate of DLAT and DLST lipoylation following the treatment of CRC cells with Cu‐PrIm nanozymes (Figure 7g), which subsequently facilitated the oligomerization of lipoylated DLAT (Figure 7h). The results of the targeted metabolomics analysis revealed an increase in pyruvate and α‐ketoglutarate levels, along with a reduction in succinate levels, in CRC cells treated with Cu‐PrIm nanozymes (Figure 7i; Figures S36 and S37, Supporting Information). These alterations were characteristic of the metabolic reprogramming processes observed during cuproptosis, and were associated with the modulation of FDX1 function. In light of the association between cuproptosis and heightened oxidative stress, we next explored the potential role of Cu‐PrIm nanozymes with POD‐like activity to induce cuproptosis. Although the administration of the antioxidant NAC did not impede the release of Cu ions (Figure 7b), it was effective in reversing the levels of key regulators of cuproptosis in Cu‐PrIm‐treated CRC cells (Figure 7e–i; Figure S36, Supporting Information). These data provide evidence that the release of Cu^2+^ and the POD‐like activity of Cu‐PrIm nanozymes synergistically facilitate the aggregation of lipoylated proteins and the destabilization of Fe‐S cluster proteins, ultimately inducing cuproptosis. The research on cuproptosis has only emerged relatively recently. In the future, a better understanding of the molecular mechanisms underlying cuproptosis may pave the way for improved treatment strategies.

The anticancer potential of the Cu ionophore elesclomol has been evaluated in clinical trials.^[^ ^31^ ^]^ However, despite demonstrating a favorable safety profile and low toxicity, elesclomol exhibited limited clinical efficacy.^[^ ^31a^ ^]^ Thus, we decided to compare Cu(II)‐elesclomol and Cu‐PrIm nanozymes in terms of their ability to release Cu ions and elicit antitumor effects, and the results showed that Cu‐PrIm nanozymes delivered more Cu ions into cancer cells than Cu(II)‐elesclomol over a longer treatment period, likely due to its greater stability (Figure 7j; Figure S38, Supporting Information). More importantly, Cu‐PrIm nanozymes were more cytotoxic than Cu(II)‐elesclomol to CRC cells when both agents were used at the same concentration (Figure 7k; Figure S39, Supporting Information). The novel Cu‐PrIm nanozymes effectively disrupt oxidative stress and provide sustained release of Cu ions, resulting in improved anticancer efficacy (Figure 7l).

### 2.6 The Chemosensitizing Effect of Cu‐PrIm Nanozymes

**Figure 8:**
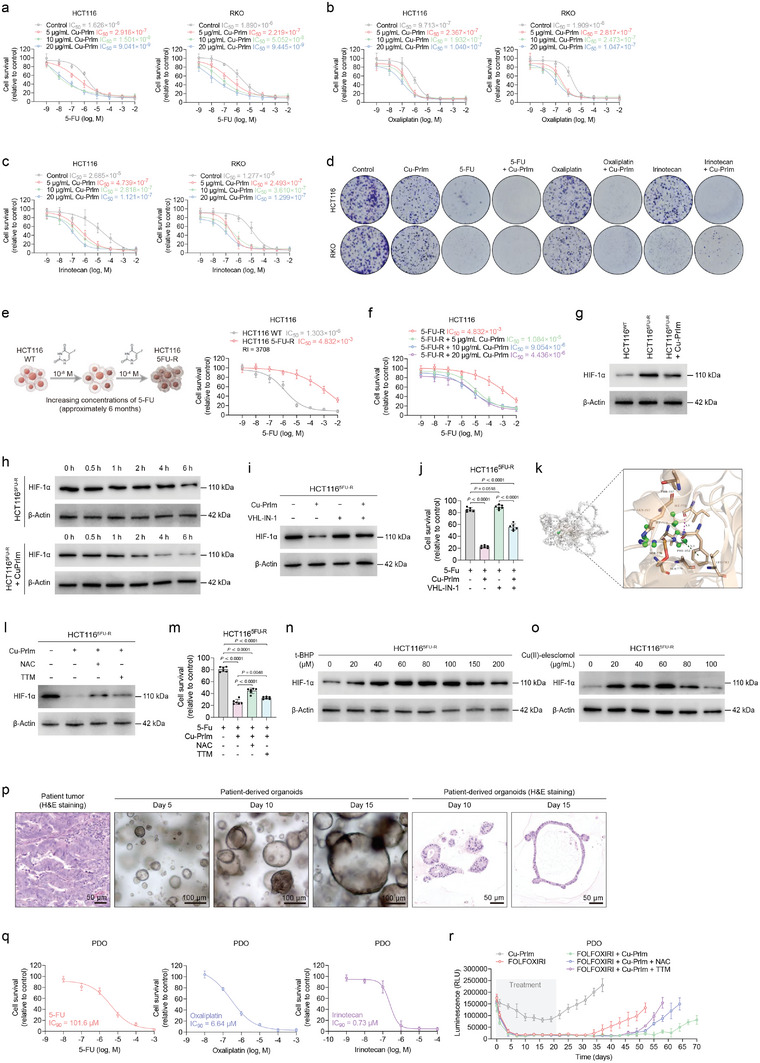
Chemosensitizing effect of Cu‐PrIm nanozymes. a–c) The viability of CRC cells treated with a gradient concentration of a) 5‐Fu, b) oxaliplatin, or c) irinotecan, as well as various concentrations of Cu‐PrIm nanozymes. d) Representative images of colony formation of CRC cells. e) Schematic diagram of the generation of 5FU‐R HCT116 cells. f) Viability of 5FU‐R CRC cells treated with a gradient concentration of 5‐FU and various concentrations of Cu‐PrIm nanozymes. g) Immunoblot of HIF‐1α in WT and 5FU‐R HCT116 cells. h) 5FU‐R HCT116 cells were treated with 5 µg mL^−1^ Cu‐PrIm or vehicle for 24 h and then cultured with 100 µm cycloheximide for 0.5 h, 1 h, 2 h, 4 h, and 6 h to compare the stability of HIF‐1α. i) Immunoblot of HIF‐1α in 5FU‐R HCT116 cells treated with indicated agents. j) Viability of 5FU‐R HCT116 cells treated with indicated agents for 24 h. k) Diagram of HIF‐1α docking with Cu‐PrIm molecule. l) Immunoblot of HIF‐1α in 5FU‐R HCT116 cells. m) Viability of 5FU‐R HCT116 cells treated with indicated agents for 24 h. n) Immunoblot of HIF‐1α in 5FU‐R HCT116 cells treated with a gradient concentration of t‐BHP for 24 h. o) Immunoblot of HIF‐1α in 5FU‐R HCT116 cells treated with a gradient concentration of Cu(II)‐elesclomol for 24 h. p) Representative images of bright field and H&E staining of organoids derived from patients with CRC. Scale bar = 50 or 100 µm. q) Viability of PDO treated with a gradient concentration of chemotherapeutic agents. r) Longitudinal response of PDOs to FOLFOXIRI and Cu‐PrIm in the presence or absence of NAC or TTM. For all studies, *n* ≥ 3. Data are shown as the mean ± SD. Comparisons were performed using the Student's *t*‐test or ANOVA. β‐Actin was used as the internal reference for immunoblot.

5‐FU, oxaliplatin, and irinotecan are chemotherapeutic agents widely used in the treatment of advanced CRC, however, the development of chemoresistance to these drugs represents a significant barrier to achieving favorable treatment outcomes.^[^ ^32^ ^]^ Therefore, it is imperative to overcome chemoresistance, increase chemosensitivity, and optimize the therapeutic outcomes of chemotherapy in the management of CRC. Studies have reported that chemotherapeutic agents induce the production of ROS, which can dampen antioxidant defense mechanisms, leading to cellular damage and death.^[^ ^17^, ^23^ ^]^ Any cells that survive the effects of chemotherapy may also have overloaded antioxidant defense systems, which render them vulnerable to the effects of oxidative stress inducers.^[^ ^23^ ^]^ Therefore, we investigated whether Cu‐PrIm nanozymes could increase the chemosensitivity of CRC cells and overcome chemoresistance through their POD‐like activity and the induction of programmed cell death. We first evaluated the potential value of combining Cu‐PrIm nanozymes with chemotherapeutic agents to enhance the chemosensitivity of CRC cells. The performance of Cu‐PrIm nanozymes either alone or in combination with 5‐FU, oxaliplatin, or irinotecan was evaluated in in vitro dose viability assays. While Cu‐PrIm nanozymes alone (at 5–20 µg mL^−1^) did not induce extensive CRC cell death, its coadministration with either 5‐FU, oxaliplatin, or irinotecan significantly enhanced the cytotoxic effects of the chemotherapeutic agent (**Figure** **8**a–c). Moreover, the combination of Cu‐PrIm nanozymes and a chemotherapeutic agent significantly reduced the proliferation of CRC cells in the colony formation than chemotherapy or Cu‐PrIm nanozymes alone (Figure 8d; Figure S40, Supporting Information).

To further investigate the role of Cu‐PrIm nanozymes in chemoresistant CRC cells, we established a cell model by subjecting wildtype CRC cells to escalating doses of 5‐FU over a period of ≈6 months. Following the completion of the treatment regimen, the resulting 5‐FU‐resistant (5FU‐R) HCT116 cells were ≈3500 times more resistant to 5‐FU than that of their parental counterparts (Figure 8e). After treating the cells with a low (5 µg mL^−1^) concentration of Cu‐PrIm nanozymes, the 5‐FU IC_50_ of 5FU‐R HCT116 cells decreased 4.4 × 10^2^ times (from 4.83 mm to 10.84 µm), and the resistance index (RI) decrease 4.7 × 10^2^ times (from 3708 to 8) (Figure 8f). The synergy between Cu‐PrIm nanozymes and 5‐FU was significant in 5FU‐R HCT116 cells, indicating that this effect was indeed related to the reversal of the chemoresistant phenotype. We have previously identified HIF‐1α as a pivotal regulatory molecule of 5‐FU resistance in CRC cells.^[^ ^17b^ ^]^ Enhanced expression of HIF‐1α was observed in both 5‐FU resistant CRC cell lines and clinical specimens, with elevated HIF‐1α levels correlating with the failure of fluorouracil analog‐based chemotherapy and reduced survival rates in CRC patients. Therefore, we then investigate whether the chemosensitization effect induced by Cu‐PrIm is mediated through HIF‐1α in 5‐FU resistant CRC. Primarily, we observed that the Cu‐PrIm nanozymes treatment led to a reduction in HIF‐1α protein expression and resulted in a significant decrease in HIF‐1α protein stability in 5FU‐R HCT116 cells (Figure 8g,h), and the glucose metabolism shifted from aerobic glycolysis to oxidative phosphorylation in Cu‐PrIm‐treated 5FU‐R CRC cells (Figure S41, Supporting Information). Subsequently, we employed VHL‐IN‐1, a stabilizer of HIF‐1α, to rescue the Cu‐PrIm‐induced decline in HIF‐1α levels, and the results demonstrated that the resistance reversal activity of Cu‐PrIm nanozymes in 5FU‐R HCT116 cells was significantly suppressed (Figure 8i,j). Furthermore, the simulation‐based interaction analysis revealed that Cu‐PrIm nanozymes can bind to the HIF‐1α protein (Figure S42, Supporting Information). The nitrogen‐containing ring in Cu‐PrIm nanozymes is capable of establishing a hydrogen bond with Ile772 at a distance of 3.1 Å, and concurrently engages in a π‐π stacking interaction with Phe352 at a distance of 5.3 Å. Additionally, the copper ions within Cu‐PrIm nanozymes form a metal coordination bond with the hydroxyl group of the Ser776 side chain. These interaction forces contribute to a binding energy of −9.59 kcal mol^−1^ for the HIF‐1α‐Cu‐PrIm complex, indicating a favorable binding affinity (Figure 8k; Figure S43, Supporting Information). HIF‐1α and Cu‐PrIm binding sites are adjacent to the Phosphofructokinase Activation (PAC) domain and the N‐terminal Von Hippel‐Lindau (VHL) recognition site, which are typically implicated in the regulation of protein stability and transcriptional activity. These results suggested that Cu‐PrIm nanozymes hold potential for overcoming chemoresistance in CRC by directly inhibiting HIF‐1α. Notably, both NAC and TTM were able to partially inhibit the Cu‐PrIm‐induced decline of HIF‐1α (Figure 8l) and reduce the Cu‐PrIm‐induced chemosensitizing effect (Figure 8m) in 5FU‐R HCT116 cells. To examine the impact of ROS and Cu ions on HIF‐1α expression in 5‐FU resistance, gradients of tert‐butyl hydroperoxide (t‐BHP, 0–200 µm) and Cu(II)‐elesclomol (0–100 µg mL^−1^) were administered to cultured 5FU‐R HCT116 cells to gradually elevate intracellular ROS and Cu ion concentrations, respectively. The results indicated that both t‐BHP and Cu(II)‐elesclomol increased HIF‐1α protein levels at lower concentrations but led to a decrease in HIF‐1α protein levels at higher concentrations (Figure 8n,o). In summary, at a low concentration that did not induce significant cell death (5 µg mL^−1^), Cu‐PrIm nanozymes effectively reduced HIF‐1α levels, thereby overcoming 5‐FU resistance in CRC. This efficacy can be attributed to two primary mechanisms: first, Cu‐PrIm nanozymes may efficiently bind to HIF‐1α to form a complex that influences protein stability; second, the potent POD‐like activity and substantial release of copper ions by Cu‐PrIm nanozymes contribute to the degradation of HIF‐1α.

The most recent evidence indicates that non‐genetic mechanisms play a significant role in the development of drug tolerance, posing a significant challenge to cancer therapy.^[^ ^33^ ^]^ DTP cells have been recognized as important contributors to the non‐genetic heterogeneity of tumors and have been detected in CRC patients undergoing chemotherapy.^[^ ^34^ ^]^ Thus, targeting DTP cells may offer a promising strategy for preventing CRC chemoresistance and recurrence before the onset of irreversible chemoresistance‐associated genetic mutations. DTP cells demonstrate a reliance on mitochondrial oxidative phosphorylation concomitant with mitochondrial oxidative stress.^[^ ^35^ ^]^ The biological properties of Cu‐PrIm nanozymes imply that they may potentially be effective in treating DTP cells in CRC. To study the induction of DTP cells by chemotherapy, we established a patient‐derived organoid (PDO) model using clinical CRC samples (Figure 8p). Initially, we determined the IC_90_ values of 5‐FU, oxaliplatin, and irinotecan in PDOs in a 3D culture system (Figure 8q). Upon reaching a diameter greater than 100 µm, the PDOs were exposed to the maximum tolerated doses of the FOLFOXIRI regimen (comprising 5‐FU, leucovorin, oxaliplatin, and irinotecan at their respective IC_90_ values). Treatment with FOLFOXIRI did not eradicate all cancer cells within the PDOs, leaving behind residual organoids. Notably, these residual cancer cells demonstrated regrowth 2 weeks after cessation of drug treatment (Figure 8r). This phenomenon bears resemblance to the persistence of residual disease and recurrence following drug withdrawal observed in CRC patients. Crucially, the combination of Cu‐PrIm and FOLFOXIRI notably delayed the recurrence of cancer cell regrowth following drug cessation, which was attributed to the disruption of oxidative balance and the release of Cu ions by Cu‐PrIm nanozymes in CRC cells (Figure 8r). These findings indicate that the combination treatment comprising FOLFOXIRI and Cu‐PrIm nanozymes may extend the duration of chemotherapeutic responses in CRC.

### 2.7 Biosafety and Biodistribution of Cu‐PrIm Nanozymes

**Figure 9:**
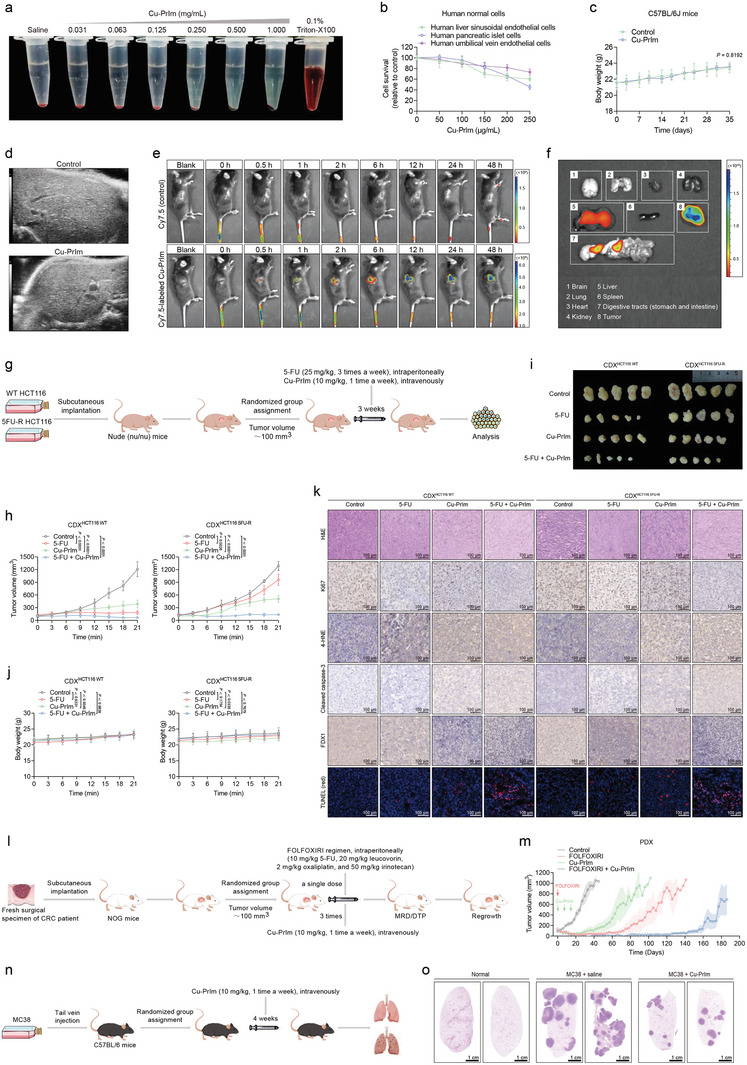
Biosafety and in vivo antitumor activity of Cu‐PrIm nanozymes. a) Representative images showing erythrocyte hemolysis after treatment with Cu‐PrIm nanozymes. b) Viability of human normal cells treated with a gradient concentration of Cu‐PrIm nanozymes. c) Weight change of C57/BL6 mice treated with PBS or Cu‐PrIm. d) Liver ultrasonography of C57/BL6 mice 2 weeks after the end of PBS or Cu‐PrIm treatment. e) Biodistribution after intravenous injection of Cy7.5‐labeled Cu‐PrIm or Cy7.5 (control) at different time points. f) Ex vivo imaging of major organs and tumor 48 h after Cy7.5‐labeled Cu‐PrIm injection. g) Schematic diagram of CDX model establishment and treatment schedule. h) Tumor growth curves for WT and 5FU‐R HCT116 xenografts with 5‐FU or Cu‐PrIm at different time points. i) Photographs of the dissected tumors from CDX mice at the end of treatment. j) Weight change of C57/BL6 mice treated with 5‐FU and Cu‐PrIm. k) Representative images of H&E staining, Ki67, 4‐HNE, cleaved caspase‐3, FDX1, and TUNEL. Scale bar = 100 µm. l) Schematic diagram of PDX model establishment and treatment schedule. m) Tumor growth curves of PDXs treated with a single dose of FOLFOXIRI or three doses of Cu‐PrIm. n) Schematic diagram of pulmonary metastasis model establishment and treatment schedule. o) H&E staining images of metastatic lung nodules after saline or Cu‐PrIm treatments. Scale bar = 1 cm. For all studies, *n* ≥ 3. Number of animals per group was 5. Data are shown as the mean ± SD. Comparisons were performed using the Student's *t*‐test or ANOVA.

Prior to evaluating the in vivo antitumor activity of Cu‐PrIm nanozymes, we conducted an assessment of its biosafety. Initially, the in vitro toxicity of Cu‐PrIm nanozymes was examined in normal human cells and tissues. The hemolysis assay was used to evaluate the potential deleterious impact of Cu‐PrIm nanozymes on red blood cells. We detected minimal levels of hemolysis even on exposure of the red blood cells to a high (1 mg mL^−1^) concentration of Cu‐PrIm nanozymes (**Figure** **9**a). Additionally, Cu‐PrIm nanozymes did not show significant cytotoxicity against the human normal colon epithelial cell line NCM460, primary liver sinusoidal endothelial cells, pancreatic islet cells, or umbilical vein endothelial cells at concentrations up to 100 µg mL^−1^; however, this same concentration of Cu‐PrIm nanozymes killed CRC cells (Figure 9b). This difference in cytotoxicity may be explained by the fact that the high H_2_O_2_ levels observed in CRC cells enhance the activity of POD‐like enzymes, while the low levels of H_2_O_2_ present in normal cells reduce the risk of nanozymes cascade reactions, thereby minimizing damage to normal cells. Moreover, the immunogenicity of Cu‐PrIm nanozymes was evaluated. Results indicated that Cu‐PrIm neither directly activated T cells nor induced a significant T‐cell proliferation response through cancer cells (Figure S44, Supporting Information). Therefore, we conclude that Cu‐PrIm itself lacks immunogenicity and cannot trigger immunogenic cell death.

Next, we performed biosafety analyses of healthy C57BL/6 male mice, which were treated with four injections of 10 mg kg^−1^ Cu‐PrIm nanozymes, administered via the tail vein, once per week. Mouse blood samples and vital organs were collected on day 14 post‐treatment for further analysis. The results showed that Cu‐PrIm nanozymes treatment did not significantly impact the body weights of mice, suggesting that the Cu‐PrIm nanozymes induced tolerable levels of systemic toxicity (Figure 9c). In addition, we examined the routine blood, blood electrolyte, and biochemical parameters of the mice (Figure S45, Supporting Information), and results indicated that there were no statistically significant differences in the neutrophil, lymphocyte, monocyte, and platelet counts or hemoglobin levels; the serum sodium, potassium, calcium, phosphorus, and chlorine levels; or the blood glucose, triglyceride, cholesterol, high‐density lipoprotein cholesterol (HDL‐C), low‐density lipoprotein cholesterol (LDL‐C), albumin, aspartate aminotransferase (AST), alkaline phosphatase (ALP), total bilirubin (T‐BIL), direct bilirubin (D‐BIL), urea nitrogen, or creatinine levels between the Cu‐PrIm‐treated and vehicle control mice. However, it is worth noting that the mice treated with Cu‐PrIm nanozymes exhibited a slight elevation in alanine aminotransferase (ALT) and γ‐glutamyltransferase (GGT) levels relative to the control group. We next determined the level of Cu ions in the major mouse organs. At 2 weeks after Cu‐PrIm nanozymes treatment termination, no significant differences in Cu ion levels in the brain, heart, liver, and kidney were detected between the Cu‐PrIm‐treated and control mice (Figure S46, Supporting Information). Subsequently, histological staining was used to examine the tissues of the major mouse organs. We found that the major organs of mice treated with Cu‐PrIm nanozymes did not exhibit any histological abnormalities (Figure S47, Supporting Information). Of note, although there was evidence of mildly abnormal liver function (i.e., increased serum ALT and GGT levels) in the mice following Cu‐PrIm nanozymes treatment (Figure S45, Supporting Information), no significant liver collagen deposition or fibrosis was observed in histological analysis. Moreover, the livers of Cu‐PrIm‐treated mice appeared to have normal echogenicity and size on the abdominal ultrasound (Figure 9d). Taken together, Cu‐PrIm nanozymes showed tolerable hepatotoxicity and systemic toxicity.

The efficacy of nanozymes as antitumor agents is contingent upon their biodistribution and availability at the tumor site. To investigate the in vivo biodistribution of Cu‐PrIm nanozymes, we used syngeneic C57BL/6 model mice, which were subcutaneously injected with mouse colon carcinoma MC38 cells to induce subcutaneous tumors. When the tumors reached a volume of 100 mm^3^, the mice were injected with Cy7.5‐labeled Cu‐PrIm nanozymes via the tail vein, and the subsequent fluorescent signals were monitored using a small‐animal live imaging system. We observed fluorescence signals in both the liver and tumor sites at 30 min after the injection of Cy7.5‐labeled Cu‐PrIm nanozymes (Figure 9e). The fluorescence intensity at the tumor site increased progressively, peaking at 12 h post‐injection, and far surpassing that of the other tissues (Figure 9e). Notably, strong fluorescence was still detected at the tumor site at 48 h after Cu‐PrIm nanozymes administration (Figure 9e). These data indicate that Cu‐PrIm nanozymes preferentially localized to the tumor. To show the biodistribution of Cu‐PrIm nanozymes in more detail, the mice were sacrificed at 48 h post‐injection, and both their tumors and vital organs were harvested for ex vivo fluorescence imaging. The results indicated that the tumor was the predominant site of Cu‐PrIm nanozymes accumulation, with a lesser extent of accumulation observed in the liver and digestive tracts (Figure 9f). These findings suggest that Cu‐PrIm nanozymes preferentially accumulates in the tumor in vivo, implying that it may exhibit efficacy against CRC with minimal side effects.

### 2.8 In Vivo Antitumor Activity of Cu‐PrIm Nanozymes

To further evaluate the antitumor and chemosensitizing effects of Cu‐PrIm nanozymes in vivo, HCT116 cells or stable 5FU‐R HCT116 cells were subcutaneously implanted into the right flank of immunodeficient nude mice. Tumor‐bearing mice were then treated with Cu‐PrIm nanozymes intravenously (via the tail vein) or 5‐FU intraperitoneally upon reaching a tumor volume of 100 mm^3^ (Figure 9g). A significant reduction in tumor volume and growth was noted in the mice bearing parental and 5FU‐R HCT116 cells after Cu‐PrIm nanozymes treatment (Figure 9h,i; Figure S48, Supporting Information), without any corresponding alterations in body weight (Figure 9j) in relation to the vehicle control. These results supported the antitumor activity of Cu‐PrIm nanozymes on CRC in vivo. We next evaluated the potential synergistic effects of Cu‐PrIm nanozymes in conjunction with chemotherapy in vivo. The findings revealed that the antitumor effect of the combination therapy was markedly greater than that of 5‐FU alone. Additionally, the efficacy of 5‐FU treatment alone or in combination with Cu‐PrIm was assessed in 5FU‐R HCT116‐bearing mice. The results indicated that Cu‐PrIm nanozymes could effectively restore the chemosensitivity of 5‐FU‐resistant tumors. Notably, all the treatment regimens used were well tolerated by the mice (Figure 9j). Collectively, these findings suggested that Cu‐PrIm nanozymes can overcome the chemoresistance of CRC tumors to 5‐FU in vivo; moreover, the combination Cu‐PrIm and 5‐FU was tolerable. Finally, histochemistry was used to evaluate the tumor tissue response to Cu‐PrIm and/or chemotherapy (Figure 9k). Evaluation of Ki67 expression in tumors aligned with the tumor size data. Specifically, the combination of Cu‐PrIm and 5‐FU reduced the level of Ki67 expression in xenograft tumors to a greater extent than either agent alone (Figure S49a, Supporting Information). Moreover, Cu‐PrIm nanozymes, either alone or in conjunction with 5‐FU, notably increased the expression of 4‐HNE, a key marker of intracellular oxidative stress (Figure S49b, Supporting Information). Additionally, an increase in TUNEL staining, caspase‐3 activation, and a decrease in FDX1 expression once again confirmed that the antitumor efficacy of Cu‐PrIm nanozymes derives from its ability to activate both the apoptotic and cuproptotic pathways (Figure S49c–e, Supporting Information).

To further investigate the effect of Cu‐PrIm nanozymes on chemotherapy‐induced DTP cells in vivo, we established PDX models using NOG mice. These mice develop DTP‐like, treatment‐naïve CRC tumors. Upon reaching a tumor volume of 100 mm^3^, the mice were treated with a single dose of the FOLFOXIRI regimen (consisting of 10 mg kg^−1^ 5‐FU, 20 mg kg^−1^ leucovorin, 2 mg kg^−1^ oxaliplatin, and 50 mg kg^−1^ irinotecan) (Figure 9l). Upon treatment, the tumors exhibited initial shrinkage, followed by regrowth (Figure 9m), consistent with the “sensitivity–DTP–regrowth” pattern observed in PDO models (Figure 8r). This suggested that FOLFOXIRI‐induced DTP could be effectively established in PDX models of treatment‐naïve primary CRC. Treating the PDX models with a combination of Cu‐PrIm nanozymes and FOLFOXIRI synergistically impeded tumor regrowth (Figure 9m). Moreover, the addition of Cu‐PrIm to the FOLFOXIRI regimen did not augment its effect on body weight (Figure S50, Supporting Information). Consistent with our in vitro findings, these results suggested that a therapeutic approach combining FOLFOXIRI with Cu‐PrIm nanozymes could potentially prolong the efficacy of chemotherapy in CRC.

Metastasis, particularly affecting the lungs (the most prevalent extra‐abdominal site for metastasis), is a primary driver of mortality in CRC.^[^ ^36^ ^]^ The process of cancer cell dissemination, facilitated by epithelial‐mesenchymal transition (EMT) and subsequent mesenchymal‐endothelial transition (MET), is widely recognized as a key characteristic of metastasis.^[^ ^37^ ^]^ Given the involvement of oxidative stress fluctuations in MET/EMT events, our study aimed to assess the impact of Cu‐PrIm nanozymes with POD‐like activity on CRC metastasis using an experimental pulmonary metastasis model (Figure 9n). The pulmonary metastasis model was established by intravenously injecting mouse CRC MC38 cells through the tail vein, followed by the immediate intravenous administration of Cu‐PrIm nanozymes. Metastases were quantified by analyzing microscopy images of pathological sections from the lungs of the mice (Figure 9o). The results showed a reduction in both the number and size of metastatic lesions in Cu‐PrIm‐treated mice relative to the control group (Figure S51, Supporting Information). Collectively, these results suggest that Cu‐PrIm nanozymes effectively inhibit the colonization and proliferation of CRC cells in the lungs.

## 3 Conclusion 

In summary, we have developed a “Three‐in‐One” Cu‐based MOF nanozyme with POD‐like activity, eliciting potent antitumor effects through a synergistic mechanism involving apoptosis and cuproptosis. Aside from inducing cell death, Cu‐PrIm nanozymes facilitate the degradation of HIF‐1α in chemoresistant CRC cells, effectively reversing chemoresistance. Our findings demonstrate the “Three‐in‐One” action of the Cu‐PrIm nanozymes, which integrates synergistic nanocatalytic therapy, cuproptosis, and chemoresistance reversal for CRC treatment both in vitro and in vivo.

Currently, an ideal agent targeting either aberrant ROS accumulation or metal metabolism in cancer cells remains elusive; however, MOF nanozymes, due to their designability, appear to be promising candidates.^[^ ^4^, ^38^ ^]^ The Cu‐PrIm nanozymes synthesized in this study selectively accumulate in cancer cells, inducing lethal oxidative stress and sustained copper ion release in vivo. Its POD‐like activity disrupts mitochondrial function and alters internal signaling to activate the endogenous mitochondrial apoptotic pathway. Furthermore, the biodegradation of Cu‐PrIm nanozymes induces cuproptosis by mediating sustained copper ion release within cancer cells. Excessive intracellular copper levels enhance the aggregation of lipoylated proteins and destabilize Fe‐S cluster proteins, ultimately inducing cuproptosis. Crucially, Cu‐PrIm nanozymes effectively reduce HIF‐1α protein levels through direct binding and indirect regulation. The combined use of 5‐FU and Cu‐PrIm partially reverses chemoresistance and increases chemosensitivity. Based on its “Three‐in‐One” action, Cu‐PrIm nanozymes effectively inhibit tumor growth, improve chemotherapeutic efficacy, overcome chemoresistance, and delay CRC recurrence.

It is conceivable that the high efficacy and low toxicity of Cu‐PrIm nanozymes may promote the development of nanozyme‐based antitumor formulations, ultimately facilitating the clinical translation of nanomedicines. Moreover, the chemoresistance‐reversing capability of Cu‐PrIm nanozymes might facilitate the therapeutic effect by serving as a combination therapy to re‐sensitize resistant CRC to the first‐line chemotherapy regimen. This study presents an innovative therapeutic paradigm that harnesses the potential of “Three‐in‐One” Cu‐PrIm nanozymes to concurrently target multiple tumor‐specific vulnerabilities.

## Conflict of Interest

The authors declare no conflict of interest.

## Author Contributions

S.D., H.C., and Y.Y. contributed equally to this work. L.C. and S.D. conceived the project. S.D., H.C., Y.Y., and L.C. designed, conducted the experiments, DFT calculations, and wrote the manuscript. Z.F., W.S., J.S., X.G., and K.G. provided relevant experimental technical support and helped with the investigations on synthesis and structural analysis of nanomaterials. S.L., Q.X., and X.Z. performed and analyzed the in vitro experiments. S.H., G.Z., L.G., and L.C. supervised this study and revised the manuscript. All authors critically revised the article and approved the final manuscript.

## Ethics Approval and Consent to Participate

All experiments involving animals were approved by the Institutional Animal Care and Use Committee of Shandong Provincial Qianfoshan Hospital. All procedures complied with the relevant ethical standards for animal testing and research. The human studies were conducted with the informed consent of all the patients involved and were approved by the Shandong Provincial Qianfoshan Hospital for research involving human subjects.

## Supporting information



Supporting Information

## Data Availability

The data that support the findings of this study are available in the supplementary material of this article.
