# Supplementary material for: A Novel “Three‐in‐One” Copper‐Based Metal‐Organic Framework Nanozyme Eradicates Colorectal Cancer and Overcomes Chemoresistance for Tumor Therapy
Source: Adv Sci (Weinh). 2024 Dec 4;12(6):2413422. doi: 10.1002/advs.202413422 (PMC11809406; doi:10.1002/advs.202413422)
Supplement: Supplementary file 1 — Supporting Information [file ADVS-12-2413422-s001.pdf]

ADVANCED SCIENCE
Open Access

Supporting Information

for *Adv. Sci.*, DOI 10.1002/advs.202413422

A Novel “Three-in-One” Copper-Based Metal-Organic Framework Nanozyme Eradicates Colorectal Cancer and Overcomes Chemoresistance for Tumor Therapy

Shuohui Dong, Haolin Cao, Ye Yuan, Shuo Liang, Zhendong Fu, Wei Shi, Qian Xu, Xiang Zhao, Jingnan Shi, Xiaoxiao Guo, Kaili Guo, Sanyuan Hu, Guangyong Zhang*, Lizeng Gao* and Lei Chen**

Supporting Information

A Novel “Three-in-One” Copper-Based Metal-Organic Framework Nanozyme Eradicates Colorectal Cancer and Overcomes Chemoresistance for Tumor Therapy

Table of Contents

Supporting Methods **5**

Chemicals, reagents and kits 5

Synthesis of M-PrIm 5

Characterization 5

POD-like activity assay of Cu-PrIm nanozymes 6

Electron Spin Resonance (ESR) analysis of the produced •OH and •O_2^- 7

GSHOx activity of Cu-PrIm nanozymes 8

Detection of Cu^{2+} release from Cu-PrIm nanozymes 8

First-principles calculations 8

Cell lines and cell culture 9

Organoid generation and culture 9

Cell viability assay 10

5-FU resistant (5FU-R) cell line generation 11

In vitro cellular uptake of nanozymes 11

Reactive oxygen species (ROS) production assay 12

Measurement of the copper ion content 12

Mitochondrial content assay 13

Oxygen-consumption rate (OCR) assay 13

Adenosine triphosphate (ATP) content assay 13

Apoptosis assay 14

Molecular docking 14

Protein extraction and western blot 15

RNA extraction and quantitative real-time PCR (RT-qPCR) 15

Transcriptome analysis 16

Immunofluorescent co-localization 16

Metabolite extraction and UPLC-MS/MS analysis 17

Colony formation assay 17

Mice housing and ethical guidelines 17

Cell line-derived xenograft (CDX) Model establishment 18

Patient-derived xenografts (PDXs) Model establishment 18

Lung metastasis model establishment 18

In vivo biosafety evaluation 19

In vivo biodistribution study 19
Hemolysis assay 19
Mixed lymphocyte reaction 20
Blood indicators detection 20
Histopathologic examination 20
Statistical analysis 21
References 21
Supporting Figures 23
Figure S1 23
Figure S2 24
Figure S3 25
Figure S4 26
Figure S5 27
Figure S6 28
Figure S7 29
Figure S8 30
Figure S9 31
Figure S10 32
Figure S11 33
Figure S12 34
Figure S13 35
Figure S14 36
Figure S15 37
Figure S16 38
Figure S17 39
Figure S18 40
Figure S19 41
Figure S20 42
Figure S21 43
Figure S22 44
Figure S23 45
Figure S24 46
Figure S25 47

Figure S26 48
Figure S27 49
Figure S28 50
Figure S29 51
Figure S30 52
Figure S31 53
Figure S32 54
Figure S33 55
Figure S34 56
Figure S35 57
Figure S36 58
Figure S37 59
Figure S38 60
Figure S39 61
Figure S40 62
Figure S41 63
Figure S42 64
Figure S43 65
Figure S44 66
Figure S45 67
Figure S46 68
Figure S47 69
Figure S48 70
Figure S49 71
Figure S50 72
Figure S51 73
Supporting Tables 74
Table S1. Key resources table 74
Table S2. Primer information 79
Table S3. UPLC-MS/MS conditions 81

Supporting Methods

Chemicals, reagents and kits

2-Methylimidazole (2-MeIm), 2-ethylimidazole (2-EtIm), 2-propylimidazole (2-PrIm), 2-butylimidazole (2-BuIm), cupric chloride ($CuCl_2$), cobalt chloride hydrate ($CoCl_2 \cdot 6H_2O$), Nickel(II) chloride hexahydrate ($NiCl_2 \cdot 6H_2O$), p-Benzoquinone (p-BQ) were purchased from Macklin. Horseradish Peroxidase (HRP), 5,5-dimethyl-1-pyrroline N-oxide (DMPO), 3,3',5,5'-Tetramethylbenzidine (TMB), Iron (II) sulfate heptahydrate ($FeSO_4 \cdot 7H_2O$), and Sodium acetate (NaAc) were purchased from Sigma-Aldric. Zinc chloride ($ZnCl_2$), acetate (HAc), Isopropyl alcohol (IPO), Hydrogen peroxide (H_2O_2) and ethanol (EtOH) were purchased from Sinopharm. Phosphate buffered saline (PBS), glutathione (GSH) was purchased from Solarbio. 5,5′-dithiobis (2-nitrobenzoic acid) (DTNB) was purchased from Bidepharm. Cuprizone was purchased from Leyan. Dimethyl sulfoxide (DMSO) was purchased from Beyotime. Amplex Red (AR) was purchased from Biosharp. Ultrapure water was used in all experiments. The chemicals, reagents, and kits employed in the biological experiments are detailed in **Table S1 (Supporting Information)**.

Synthesis of M-PrIm

The M-Prim nanoparticles were synthesized via an ultrasonic self-assembly method. Take Cu-PrIm nanozymes as an example, first, $CuCl_2$ ($CuCl_2$, 4 mmol) was dissolved in ethanol/H_2O (40 mL, 1:3 v/v) solution (Solution A). 2-Propylimidazole (40 mmol) was also dissolved in methanol/H_2O (40 mL, 1:3 v/v) solution (Solution B). Subsequently, Solution A was added dropwise to Solution B under sonication for 1 h. The Cu-PrIm nanozymes in the mixture were collected by centrifugation at 10,000 rpm for 10 min, following which it was subjected to three washes with ethanol and water. The obtained Cu-PrIm nanozymes were then freeze-dried in a vacuum lyophiliser overnight after centrifugation. Other M-PrIm was synthesized by the same way with different metal salt involving $FeSO_4 \cdot 7H_2O$, $CoCl_2 \cdot 6H_2O$, $NiCl_2 \cdot 6H_2O$ and $ZnCl_2$.

Characterization

X-ray diffraction (XRD) of all materials were conducted on a D8 ADVANCE X (Bruker AXS, Germany), where copper target was chosen. Scanning electron microscopy (SEM) images were observed on an S-4800II (Hitachi, Japan). Fourier transform infrared (FT-IR) analysis were performed on a Nicolet IS10 (Thermo Scientific, USA) IR spectrometer TEM and SAED images were obtained with a FEI Tecnai G2 F30 (FEI, USA) operated at 300kV. X-ray photoelectron spectroscopy (XPS) measurements were conducted on an ESCALAB 250Xi (Thermo Scientific, USA). The elemental ratio was detected on an Inductively coupled plasma optical emission spectrometer (ICP-OES, Agilent ICPOES 730, USA) and an organic element analyzer (UNICUBE, Elementar, German) The X-ray Absorption Fine Structure (XAFS) spectra (Fe K-edge) were collected at BL16U1 station in Shanghai Synchrotron Radiation Facility (SSRF, 3.5 GeV, 250 mA). The XAFS data of the samples were collected at room temperature. The samples were pelletized as disks of 13 mm diameter with 1 mm thickness using graphite powder as binder. UV–vis absorption spectra were recorded on a spectrophotometer (INESA-757) and a microplate reader (VICTOR Nivo).

POD-like activity assay of Cu-PrIm nanozymes

The POD-like activity of M-PrIm nanozymes was assessed using TMB as chromogenic agent in the presence of H_2O_2. To evaluate the POD-like activity of M-PrIm, 10 μL of Cu-PrIm (200 μg/mL) was added into 90 μL of 0.2 M HAc−NaAc buffer solution (pH 4.5) containing 2 μL of TMB (10 mg/mL in DMSO) and 5 μL of H_2O_2 (1 M). The absorption at 652 nm of the mixture was measured by a miniature tablet reader every 30s. The test of OXD-like activity of M-PrIm was carried out as the same way without the addition of H_2O_2.

The pH-dependent POD-like activity assay of Cu-PrIm was conducted by employing 0.2 mg/mL nanozymes the same as previously described in buffers with different pH values (4.5, 5.5, 6.5, 7.4). The absorbance ranging from 500-750 nm was detected 10 min after the start of the reaction.

The steady-state kinetic assays of Cu-PrIm nanozymes with H_2O_2 as the substrate were performed by adding 10 μL of 0.2 mg/mL nanozymes into 90 μL of 0.2 M HAc−NaAc buffer solution (pH 4.5) containing 2 μL of TMB (10 mg/mL in DMSO) and 5 μL of different concentrations of H_2O_2 (0.625, 1.25, 2.5, 5, 10, 20, 40 mM).

The velocity values (v) of catalytic reaction was calculated by following equation:

$$v = \frac{\Delta A}{\varepsilon \times l \times \Delta t}$$

where ΔA represents the change of absorbance after catalytic reaction; V represents the total volume of reaction solution; Δt represents the time of catalytic reaction (1 min); ε represents the molar absorption coefficient of TMB (39,000 $mol^{-1} \cdot L \cdot cm^{-1}$); l represents the optical path of the cuvette (cm).

The Michaelis constant (K_m) in enzymology is the substrate concentration required for half of the maximum enzyme activity, reflecting the affinity of the nanozyme for its substrate. The maximum rate of the reaction (v_{max}) is the highest rate of the reaction observed at saturating substrate concentrations. K_m and v_{max} of iron sulfide nanozymes were calculated using GraphPad Prism 7.0 software by fitting initial reaction rates (v) and substrate concentrations to Michaelis-Menten equations as follows:

$$v = \frac{v_{max} \times [S]}{K_m + [S]}$$

where $[S]$ represents the concentration of the substrate.

The free radical quenching experiment was carried out using IPO and p-BQ as quenching agents for •OH and $•O_2^-$ respectively. Typically, 100 μL of Cu-PrIm (200 μg/mL) was added into 900 μL of 0.2 M HAc–NaAc buffer solution (pH 4.5) containing 20 μL of TMB (10 mg/mL in DMSO), 5 μL of H_2O_2 (1 M) and 200 μL IPO (or 1 mM p-BQ). The absorbance ranging from 500-750 nm was detected 10 min after the start of the reaction.

Electron Spin Resonance (ESR) analysis of the produced •OH and $•O_2^-$

ESR measurements were carried out using a Bruker electron spin resonance (ESR) spectrometer (A300-10/12, Germany) at ambient temperature. Typically, 200 μL of DMPO (100 mM) and 100 μL of Cu-PrIm (200 μg/mL) were mixed in a 0.2 M HAc–NaAc buffer solution (pH 4.5) in a capillary tube, and the reaction was then induced with H_2O_2 (20 mM) for DMPO/• OH detection. The ESR signals were collected at 20 min after the reaction. For DMPO/$•O_2^-$ assay, the same procedure described above was conducted with the addition of 1 mL IPO as the quenching agent of •OH.

GSHOx activity of Cu-PrIm nanozymes

The GSHOx activity of Cu-PrIm was detected by DTNB as a chromogenic probe which could react with the free sulfhydryl group (-SH) of GSH and form a product with characteristic absorption at 412 nm. Since GSH is readily oxidized upon exposure to light, all tests were conducted in the dark. First, 0.2 mg/mL of OISF-TPA nanozyme was incubated with GSH (0.4 mM) in 1X PBS for 30 min. The mixture was then centrifugated (10000 rpm for 5 min) and the concentration of remaining GSH in supernatant was measured by DTNB solution (1 mM). To detect whether the H_2O_2 was generated in GSHOx reation, Amplex red was utilized as the probe. In brief, Cu-PrIm (0.1 mg/mL), GSH (0.4 mM), Amplex red (10 μg/mL) and HRP (10 μg/mL) were in pH 4.5 HAc-NaAc buffer solution for 30 min. The absorbance at 400-700 nm of the supernatant was measured on a microplate reader (VICTOR Nivo). For steady-state kinetic analysis, Cu-PrIm (0.1 mg/mL) was reacted with different concentrations of GSH (0, 0.1, 0.2, 0.4, 0.6, 0.8, 1 mM) for 20 min, and the supernatant was obtained by centrifugation. Then, DTNB solution (1 mM) was utilized to detect the concentration of remaining GSH in the supernatant. The Michaelis-Menten saturation curve generated by GraphPad Prism 8.0 (GraphPad Software) was used to determine the Michaelis-Menten constant.

Detection of Cu^{2+} release from Cu-PrIm nanozymes

The release of Cu^{2+} from Cu-PrIm was detected using cuprizone as the probe. Typically, the Cu-PrIm (200 μg/mL) was firstly incubated in water and pH 4.5 HAc-NaAc Buffer for 1h, subsequently, the supernatant was obtained by centrifugation at 10,000 rpm for 5 min. In the Cu^{2+} assay, 130 μL pH 4.5 HAc-NaAc Buffer, 20 μL of Cu-PrIm supernatant (200 μg/mL) and 50 μL of Cuprizone solution (20 mM, dissolved in DMSO) were added in microplate in order. Following a 20-minute incubation period, the characteristic absorbance of Cu-cuprizone complex at 620 nm was measured. Concentration of Cu^{2+} released from Cu-MeIm, Cu-EtIm and Cu-BuIm was determined by the same methodology previously described above.

First-principles calculations

First principle density functional theory (DFT) calculations had been done using Vienna ab

initio Simulation Package (VASP)[1]. The electron-ion interactions were described by using projector augmented wave (PAW) method[2]. The generalized gradient approximation (GGA) was used with the exchange-correlation functional of Perdew-Burke−Ernzerhof (PBE)[3]. All geometry optimizations and energy calculations were performed in a plane-wave basis set up to an energy cutoff of 520 eV with a first-order Methfessel−Paxton[4] smearing of 0.1 eV. We employed a one-layered slab in (001) direction with a (1×1) unit cell in the lateral direction to model the Cu-PrIm surface. To separate the slab from its periodic images to avoid spurious interaction, a vacuum height of 15 Å along the vertical direction was selected. The calculations were performed using the (2×2×1) Monkhorst-Pack mesh kpoints[5] for (1×1) unit cell. Conjugated-gradient algorithm was used to optimize the structures. In all calculations, the convergence criterion of electronic structures was set to 10^{-6} eV, and the atomic positions were allowed to relax until the forces were less than 0.02 eV/Å. The adsorption energies were calculated in the following expressions:

$$E_{ads} = E_{slab+mol} - (E_{slab} + E_{mol})$$

where $E_{slab+mol}$ represents the total energies of the chosen surface with adsorbate on it, and the E_{slab} and E_{mol} denote the bare chosen surface and the adsorbate, respectively.

Cell lines and cell culture

The human CRC cell lines HCT116 and RKO, the murine colon cancer cell line MC38, and the human normal colon epithelial cell line NCM460 were procured from Oricell (Cyagen Biosciences). Short tandem repeat profiling was utilized to confirm the authenticity of these cell lines, and a mycoplasma PCR detection kit (Sigma-Aldrich) was employed to ensure the absence of mycoplasma contamination in all tested cell lines. The cells were cultured in Roswell Park Memorial Institute 1640 medium (RPMI-1640, Gibco) containing 10% fetal bovine serum (Gibco), 100 U/mL penicillin, and 100 μg/mL streptomycin (Gibco) at 37 °C with 5% CO_2. Passage of cells was conducted using 0.25% trypsin-EDTA digestion (Biosharp). The cells were cultured in 10 cm plates and later transferred to 6-, 24-, or 96-well plates as per the experimental requirements.

Organoid generation and culture

The CRC tumor specimens were obtained from resections conducted at Shandong Provincial Qianfoshan Hospital, with the explicit written consent of all patients and approval from the hospital's research ethics committee. The establishment of CRC patient-derived organoids (PDOs) was adapted from techniques developed by the Clevers Lab[6]. In summary, freshly obtained CRC tumor specimens were finely minced and incubated at 37°C with a digestion solution (AimingMed) for 45–60 min; the progress monitored through visual examination using a stereomicroscope. The digestion process was stopped by diluting the CRC tumor specimens with PBS, followed by filtration through a 100 μm nylon cell strainer and centrifugation at 300 × g for 5 min. The precipitated cells were washed and mixed with Matrigel (Corning). Subsequently, 10,000 cells were seeded in a 24-well plate. Once the Matrigel solidified, the samples were cultured in MasterAim CRC organoid medium (AimingMed), with half of the medium replaced every 3 d. When the majority of organoids exceeded 100 μm in diameter, the PDOs were dissociated using TrypLE (Gibco) and seeded in Matrigel in a 96-well plate for subsequent analysis.

Cell viability assay

The viability of HCT116 and RKO cell lines was assessed using the cell counting kit-8 (CCK-8, MedChemExpress) following the manufacturer's instructions. The cells were seeded at a density of 1,000–5,000 cells in a 96-well plate and treated with nanozymes or chemicals for the specified durations. Subsequently, 10% (v/v) CCK-8 reagent was added and incubated at 37 °C for 0.5–1 h. The absorbance was measured at 450 nm using a microplate reader (Bio-Rad) to determine the relative cell viability, which was calculated as follows: cell viability (%) = (Abs treated–Abs blank) / (Abs control–Abs blank) × 100.

The viability of the PDOs was evaluated using CellTiter-Glo 3D (Promega). Upon reaching a diameter of 100 μm, the culture media was replaced with fresh media containing different concentrations of the drug. The PDOs were exposed to the drug for 72 h, with additional doses administered every 24 h. Following the treatment, 100 μL of CellTiter-Glo 3D (Promega) was added to each well, and the Matrigel was disrupted through mechanical means. The contents were thoroughly mixed on a plate shaker for 5 min, incubated at room temperature for 25 min, transferred to opaque-walled plates, and the luminescence was

measured using a multifunctional microplate reader (PerkinElmer). For DTP formation and regrowth, organoids were harvested once every three-day interval, and detected using CellTiter-Glo 3D (Promega).

5-FU resistant (5FU-R) cell line generation

The cells were subjected to sustained stimulation through an incremental increase in 5-FU concentrations (ranging from 10^{-8} to 10^{-4} M). The assessment of 5-FU resistance was conducted by evaluating the resistance index (RI) following a two-week cessation of 5-FU treatment, with RI values exceeding 100 indicating successful development of resistance[7]. Stably acquired 5FU-R HCT116 cells with an RI of approximately 3,500, were used as an *in vitro* model in this study.

***In vitro* cellular uptake of nanozymes**

The HCT116 and RKO cell lines were seeded at a density of 1×10^5 cells/well in 24-well plates containing cell-climbing slices to achieve 60% confluence. The cell lines were then treated with various 40 μg/mL Cy7.5-labeled nanozymes and incubated for an additional 24 h. Following this incubation period, the cell-climbing slices were removed and the cell lines were washed thrice with PBS. Subsequently, the cell lines were fixed with 4% paraformaldehyde (Servicebio) for 15 min, washed thrice with PBS, and counterstained with DAPI (Abcam) for 5 min. The red fluorescence of Cy7.5, was visualized and evaluated using an IX73 fluorescence microscope (Olympus).

The localization of nanozymes within CRC cell lines was investigated using fluorescence colocalization and transmission electron microscopy (TEM). For the analysis of lysosomal co-localization, cells treated with Cy7.5-labeled nanozymes were rinsed with PBS, fixed with 4% paraformaldehyde (Servicebio), and permeabilized with 0.2% Triton X-100 (Servicebio). The cell-climbing slices were then incubated with a primary antibody targeting lysosomal-associated membrane protein 1 (LAMP1, Abcam, 1:200) and a CoraLite488-conjugated goat anti-rabbit IgG(H+L) secondary antibody (Proteintech, 1:250). The cell-climbing slices were subsequently affixed to slides using a mounting medium containing DAPI (Abcam), and the fluorescence signal was captured using a TCS SP8

confocal laser scanning microscope (Leica). The co-localization analysis was performed using Pearson's correlation coefficient with ImageJ software (version 1.8.0).

For TEM analysis, the cells were seeded in 10 cm plates and allowed to grow to 60% confluence. A concentration of 40 μg/mL Cu-PrIm was introduced into the culture dish for a 24-h incubation period. Subsequently, the cells were washed, digested, centrifuged, collected, and fixed in 2.5% glutaraldehyde (Servicebio) for 24 h at 4 °C. The cells were pre-embedded in 1% agarose and fixed in 1% osmium acid (Ted Pella, Inc.) for 2 h at room temperature. Subsequently, they were dehydrated using an alcohol-acetone gradient. Dehydrated cells were processed for infiltration and embedding, followed by cutting into 70 nm sections using an ultramicrotome (Leica). These sections were evenly spread onto 150 mesh cuprum grids (Zhongxing Bairui) using a Formvar film. Subsequently, the ultrathin sections were double-stained with 2% uranium acetate and 2.6% lead citrate (Sigma-Aldrich). Finally, the dried cuprum grids were examined using a HT7800 transmission electron microscope (Hitachi).

Reactive oxygen species (ROS) production assay

The HCT116 and RKO cell lines were cultured in a 6-well plate and treated with specific chemicals or nanozymes. Upon reaching 80% confluence, intracellular levels of ROS were measured using various ROS-sensitive fluorescent probes, including DCFH-DA (Ex/Em = 488/525 nm), HKPerox-2 (Ex/Em = 480/527 nm)[8], HKOH-1r (Ex/Em = 490/520 nm)[9], and HKSOX-1r (Ex/Em = 509/534 nm)[10], were deployed to detect total ROS, H_2O_2, OH·, and $O_2^{\cdot -}$, respectively. Each assay included both positive and negative controls. The probe-loaded CRC cell lines were analyzed using an ID7000™ spectral cell analyzer flow cytometer (Sony), and the resulting data were processed using FlowJo software (version 10).

Measurement of the copper ion content

The cells were cultured in a 6-well plate and subjected to various stimuli. Prior to quantification of copper ions, trichloroacetic acid was used to eliminate potential protein interference in the cell lysates. Copper ion levels were determined using a copper assay kit (Abcam), following the manufacturer's instructions. Optical density readings of both the

samples and standards were obtained at 359 nm using a microplate reader (Bio-Rad). The copper ion concentration was adjusted based on the cell count.

Mitochondrial content assay

The HCT116 and RKO cell lines were cultured in a 24-well plate until reaching 60% confluence, followed by treatment with various concentrations of 40 μg/mL nanozymes for 24 h. Subsequently, the mitochondria in viable cells were labeled with MitoTracker Green (Beyotime) for 30 min, independent of the mitochondrial membrane potential. The Hoechst 33342 staining solution for live cells (Beyotime) was used for nuclear staining. After thoroughly washing with PBS, 24-well plate were examined using an Axio Vert.A1 inverted fluorescence microscope (Zeiss).

Oxygen-consumption rate (OCR) assay

Initially, 20, 000 cells were plated onto XFe96 cell culture plates and allowed to adhere overnight. Subsequently, the cells were treated with specific chemicals or nanozymes for 24 h. Following this incubation period, the cells were rinsed with RPMI-1640 medium and incubated at 37 °C for 1 h in a CO_2-free environment to ensure temperature and pH equilibration prior to assessment. A Seahorse XFe96 extracellular flux analyzer (Agilent) was used to detect the OCR. Mitochondrial respiratory activity was assessed by treating cells with oligomycin (1.5 μM), FCCP (1 μM), and rotenone/antimycin A (0.5 μM) in succession using a Seahorse XF Cell Mito stress test kit (Agilent). The OCR values were automatically captured using the Seahorse Wave Controller (version 2.6), and basal and maximal respiration were computed to assess mitochondrial respiration.

Adenosine triphosphate (ATP) content assay

The HCT116 and RKO cell lines were cultured in a 6-well plate and exposed to specific chemicals or nanozymes. After thorough washing, the cells were lysed to assess ATP and protein levels. The ATP levels were quantified using an ATP assay kit (Beyotime), according to the manufacturer's instructions. The lysates were centrifuged at 12,000 g for 5 min at 4 °C, and the resulting supernatant was utilized for further analysis. The ATP levels were

determined using a microplate luminometer (Promega) based on a standard curve. Protein concentrations were measured using a BCA protein assay kit (Beyotime) to normalize the ATP levels.

Apoptosis assay

For flow cytometric analysis, the cells treated with nanozymes or chemicals were seeded in a 6-well plate. Subsequently, cells were collected, washed with PBS, fixed with 4% paraformaldehyde (Servicebio), and permeabilized with 0.1% Triton X-100 (Servicebio). The TUNEL assay was conducted using the one-step TUNEL cell apoptosis detection kit (Beyotime). Following the incubation period, the apoptosis ratio was determined by analyzing the green fluorescence-positive cells using an ID7000™ spectral cell analyzer flow cytometer (Sony).

To distinguish between early and late apoptosis, an Annexin V-FITC/Propidium iodide (PI) assay (Bestbio) was conducted. The cells were collected and stained with Annexin V-FITC for 15 min, followed by PI for 5 min. The green or red fluorescence intensity was detected using an ID7000™ spectral cell analyzer flow cytometer (Sony). Subsequently, flow cytometry data were analyzed using FlowJo software (version 10).

Molecular docking

First, hydrogen atoms were added using UCSF Chimera[11]. The AMBER14SB force field was used to calculate atomic charges of the HIF-1α protein, and H++3 online tool was employed to calculate and assign amino acid pKa values under neutral conditions[12]. The three-dimensional structure of Cu-PrIm was generated using the open-source cheminformatics software RDKit, followed by conformational sampling. The lowest energy conformations were optimized using the MMFF94 force field, and the AM1-BCC partial charges were assigned using UCSF Chimera[13]. Molecular docking experiments were conducted using the AutoDock4.2 software[14]. A global search mode was used to predict binding sites, with the docking box set to a cube of 22.5 Å edge length and a spacing step of 0.375. The maximum number of conformations to be searched was set to 10,000, and a genetic algorithm was used for conformation sampling and scoring. The conformations were ranked based on docking

scores, and the optimal conformation was selected.

Protein extraction and western blot

The CRC cell lines were lysed using RIPA buffer (Beyotime) supplemented with protease (Epizyme) and phosphatase inhibitors (epizyme). Following sonication, the total lysate was centrifuged and the resulting supernatant was quantified using a BCA protein assay kit (Beyotime). Subsequently, the protein solution was boiled for 10 min and loaded onto a 10% sodium dodecyl sulfate-polyacrylamide gel electrophoresis gel (Epizyme). After electrophoresis, the proteins were transferred to PVDF membranes (Millipore). The membranes were then incubated with blocking solution for 1 h, followed by overnight incubation at 4 °C with primary antibodies, including anti-Bcl-2 (Abcam, 1:2000), anti-Bax (Abcam, 1:5000), anti-Caspase-3 (D3R6Y) (CST, 1:500), anti-Caspase-8 (D35G2) (CST, 1:1000), anti-Caspase-9 (CST, 1:1000), anti-FDX1 (Proteintech, 1:2000), anti-LIAS (Proteintech, 1:2500), anti-ACO2 (Proteintech, 1:5000), anti-ETFDH (Abcam, 1:1000), anti-NDUFV1 (Abcam, 1:1000), anti-NDUFS8 (Abcam, 1:4000), anti-Lipoic acid (Abcam, 1:1000), anti-DLAT (CST, 1:1000), and β-Actin (Proteintech, 1:5000). The signal was generated following a 2-hour incubation at room temperature with the appropriate secondary antibodies: horseradish peroxidase (HRP)-labeled goat anti-rabbit IgG (H+L) antibody (ZSGB-Bio, 1:5000) and HRP-labeled goat anti-mouse IgG (H+L) antibody (ZSGB-Bio, 1:5000). After thoroughly washing with TBST, the membranes were exposed to a chemiluminescent HRP substrate (Millipore), and the resulting chemiluminescent signals were quantified using an Odyssey Fc imager (Li-cor).

RNA extraction and quantitative real-time PCR (RT-qPCR)

TRIzol reagent (Thermo Fisher Scientific) (1 mL) was added to each well of a 6-well plate to extract RNA, which was quantified using a NanoDrop spectrophotometer (NanoDrop). Subsequently, 1,000 ng of total RNA was reverse-transcribed into complementary DNA (cDNA) using the ReverTra Ace qPCR RT kit (Toyobo). RT-qPCR was performed using SYBR Green PCR master mix (Toyobo) on a LightCycler 480 II instrument (Roche). Relative expression levels were determined using the $2^{-\Delta\Delta Ct}$ method with *ACTB* as the reference gene.

The primer sequences are provided in **Table S2 (Supporting Information)**.

Transcriptome analysis

Total RNA was extracted using the TRIzol reagent (Thermo Fisher Scientific) according to the manufacturer's protocol. Subsequently, mRNA was isolated from the total RNA by purification using Dynabeads oligo (dT) (Thermo Fisher). Following purification, mRNA was fragmented into shorter fragments using divalent cations at elevated temperatures. The cleaved RNA fragments were reverse transcribed into cDNA using SuperScript II Reverse Transcriptase (Invitrogen). cDNA was further used to synthesize U-labeled second-stranded DNAs using *Escherichia coli* DNA polymerase I (NEB), RNase H (NEB), and dUTP solution (Thermo Fisher). Subsequently, an A-base was incorporated at the blunt end of each strand to facilitate ligation to the indexed adapters. The adapters featured a T-base overhang for the ligation of the adapter to the A-tailed fragmented DNA. Following treatment with heat-labile UDG enzyme (NEB) to remove U-labeled second-stranded DNAs, the ligated products were subjected to PCR amplification. Finally, 2 × 150 bp paired-end sequencing (PE150) was performed on an Illumina NovaSeq 6000 (LC-Bio) platform. Bioinformatics analysis encompasses the sequential steps of sequencing and filtering of clean reads, alignment with a reference genome, quantification of gene abundance, and analysis of differentially expressed genes.

Immunofluorescent co-localization

The cells were cultured on cell-climbing slices in a 24-well plate, fixed with 4% paraformaldehyde (Servicebio), permeabilized with 0.2% Triton X-100 (Servicebio), blocked with goat serum (ZSGB-Bio), and incubated with anti-TOMM40 (Abcam, 1:100) and anti-cytochrome C (Abcam, 1:200) primary antibodies overnight at 4 °C. After incubation with the corresponding fluorescently labeled secondary antibodies, CoraLite488-conjugated goat anti-rabbit IgG(H+L) (Proteintech, 1:250) and CoraLite594-conjugated goat anti-mouse IgG(H+L) (Proteintech, 1:250) were used. The cell-climbing slices were mounted onto slides with a mounting medium containing DAPI (Abcam) and imaged using a TCS SP8 confocal laser-scanning microscope (Leica). Subsequently, co-localization analysis was conducted

using Pearson's correlation coefficient with the ImageJ software (version 1.8.0).

Metabolite extraction and UPLC-MS/MS analysis

The cell samples were collected, lysed using 80% aqueous methanol, sonicated to break down the cells, and centrifuged at 18,000 × g for 15 min. Subsequently, 40 μL of the resulting supernatant was transferred to a 96-well plate. 20 μL of 200 mM 3-NPH (Sigma) and 20 μL of 120 mM EDC (Sigma) were added to each well, and the reaction was conducted at 1,450 rpm and 30 °C for 60 min. The cell samples were then diluted with 350 μL of ice-cold methanol solution and centrifuged at 4,000 × g and 4°C for 20 min. Finally, 150 μL of the supernatant was transferred to a new 96-well plate for detection. Analysis was conducted using a UPLC-MS/MS instrument comprising an Acquity I-class UPLC system (Waters) and a Xevo TQ-S triple quadrupole mass spectrometer (Waters). Details of the UPLC-MS/MS conditions are provided in **Table S3 (Supporting Information)**. The data obtained from the UPLC-MS/MS analysis were processed using MassLynx software (version 4.1) to perform peak integration, calibration, and quantitation of each metabolite.

Colony formation assay

A total of 500 cells were seeded in a 6-well plate containing complete RPMI-1640 medium. The medium was refreshed every 3 d during the 10-d culture period. Subsequently, the cell clones were rinsed twice with PBS, fixed with 4% paraformaldehyde (Servicebio) for 20 min, and stained with 0.1% crystal violet solution (Solarbio) for an additional 20 min. The cells were subjected to image capture. For quantification, crystal violet was eluted with 33% acetic acid, and the resulting solution was quantified by measuring absorbance at 490 nm.

Mice housing and ethical guidelines

The C57BL/6 mice (C57BL/6NCrl), BALB/c nude mice (CAnN.Cg-*Foxn1*^{nu}/Crl), and NOG mice (NOD.Cg-*Prkdc*^{scid}*Il2rg*^{tm1Sug}/JicCrl) were purchased from Weitonglihua Biotechnology. The animal experimentation protocols were approved by the Institutional Animal Care and Use Committee of Shandong Provincial Qianfoshan Hospital and adhered to the ethical standards for animal research. Five mice were housed together per cage in specific-pathogen-free (SPF)

conditions, with environmental parameters maintained at 25 ± 1 °C, 50%–70% relative humidity, and a 12-h light/12-h dark cycle. Mice were provided with filtered tap water and a standard rodent diet. In the current study, no infections, wounds, or significant loss of body weight were observed in any mouse.

Cell line-derived xenograft (CDX) model establishment

The cell suspensions containing either 1×10^6 WT or 5FU-R HCT116 cells in 100 μL of diluted Matrigel (Corning) were subcutaneously injected into the right forelimb underarm of four-week-old male athymic BALB/c nude mice. The mice were randomly assigned to eight groups, with five mice in each group, and monitored until their tumors reached a volume of approximately 100 mm^3. Different groups of mice were intraperitoneally injected with 5-FU (25 mg/kg, three times per week), Cu-PrIm (10 mg/kg, once weekly, via the tail vein), or saline (control). Tumor growth and changes in body weight were monitored every 3 d. After a three-week period, the mice were euthanized and the tumor mass was quantified. The tumors were harvested and preserved for subsequent histological examination.

Patient-derived xenograft (PDX) model establishment

Fresh CRC tissues were finely minced using a scalpel and subsequently combined with Matrigel (Corning). Small pieces of human CRC tissue were subcutaneously implanted into the right armpit of highly immunodeficient NOG mice. The xenografts were maintained and passaged once the tumors reached a volume of 1000 mm^3.

In NOG mice bearing subcutaneous xenografts of previously established PDX tumors, drug administration commenced once the tumors reached a volume of approximately 100 mm^3. Mice was randomly assigned to receive a single dose of the FOLFOXIRI regimen (10 mg/kg 5-FU, 20 mg/kg leucovorin, 2 mg/kg oxaliplatin, and 50 mg/kg irinotecan), Cu-PrIm (10 mg/kg), or saline (negative control). Tumor growth and changes in the body weight of the animals were closely monitored throughout the study.

Lung metastasis model establishment

In the pulmonary metastasis model, MC38 cells were introduced via tail vein injection,

followed by intravenous administration of Cu-PrIm (10 mg/kg) or saline (as a negative control). Following a four-week period, the mice were euthanized and their lungs were removed. Metastases were assessed through quantification of nodules present on the lung surface and analysis of microscopic images from pathological sections.

In vivo biosafety evaluation

The healthy male C57BL/6 mice were randomly assigned to groups and administered PBS or Cu-PrIm. The Cu-PrIm treatment, at a dosage of 10 mg/kg, was delivered via the tail vein once weekly for a total of four administrations. Throughout the study, the mice were monitored regularly and weighed twice weekly. At the conclusion of the 14-d period following treatment initiation, the mice were euthanized, and blood samples and vital organs were collected for subsequent analysis.

In vivo biodistribution study

C57BL/6 mice were subcutaneously inoculated with MC38 cells to induce the growth of subcutaneous tumors, which were subsequently used to assess the Cu-PrIm biodistribution. Once the tumors reached a volume of 1000 mm^3, Cy7.5-labeled Cu-PrIm was administered intravenously via the tail vein, and the resulting fluorescent signals were monitored using a small animal live imaging system. Biodistribution images of Cy7.5-labeled Cu-PrIm were obtained at various time points post injection using an IVIS Spectrum *in vivo* imaging system (PerkinElmer) in tumor-bearing mice. In the *ex vivo* biodistribution study, mice were euthanized 48 h after injection. Organs such as the brain, lungs, heart, kidneys, liver, spleen, digestive tract (stomach and intestine), and tumors were imaged *ex vivo*.

Hemolysis assay

The hemolysis experiment was approved by the Shandong Provincial Qianfoshan Hospital. Peripheral venous blood was collected from the healthy adult participants. The blood was then centrifuged at 5,000 rpm for 10 min, resulting in the removal of the top plasma layer and the mid-white layer of leukocytes. The remaining red blood cell (RBC) pellet was washed with normal saline and diluted to 2% by volume. Different gradient concentrations of

Cu-PrIm were then added to the RBC suspension, with normal saline and 1% Triton X-100 serving as negative and positive controls, respectively. The mixture underwent incubation for a duration of 2 h at 37 °C, followed by centrifugation for 10 min at a speed of 5,000 rpm. The optical density of the resulting supernatant was measured at 540 nm by using a microplate reader (Bio-Rad). Control samples included 0% hemolysis using normal saline and 100% hemolysis using Triton X-100.

Mixed lymphocyte reaction

Purified T cells derived from the PBMCs of healthy donors were used as responder cells. $CD4^+$ and $CD8^+$ T cells were selectively enriched by negative immunomagnetic selection using human $CD4^+$ T cell enrichment kits (StemCell) and human $CD8^+$ T cell enrichment kits (StemCell). Irradiated immature DCs served as inducers and interacted with allogeneic T cells in a 96-well plate. Additionally, HCT116 cells were cultured in HTS 96-well Transwell inserts (Corning), with or without treatment with Cu-PrIm, functioning as “third-party cells.” After a period of 5 d, the proliferation of $CD4^+$ and $CD8^+$ T cells was quantitatively assessed using the CCK-8 assay, with measurements conducted using a microplate reader (Bio-Rad).

Blood indicators detection

Blood was obtained via orbital vein bleeding during euthanasia. Whole blood was collected in anticoagulation tubes and routinely analyzed using a hematology analyzer (Mindray). Additionally, a separate sample was centrifuged at 3,000 rpm for 10 min to obtain plasma, which was then analyzed for various biochemical parameters using an automatic biochemical analyzer (Rayto). These parameters included blood electrolytes, blood glucose, triglyceride, cholesterol, high density lipoprotein cholesterol (HDL-C), low density lipoprotein cholesterol (LDL-C), albumin, and aminotransferase (ALT), aspartate aminotransferase (AST), alkaline phosphatase (ALP), y-glutamyltransferase (GGT), total bilirubin (T-BIL), direct bilirubin (D-BIL), urea nitrogen, and creatinine.

Histopathologic examination

The organ and tumor samples were fixed in 4% paraformaldehyde (Servicebio), embedded in

paraffin, and sectioned into 5 μm slices. Following deparaffinization in xylene and rehydration with graded ethanol, the sections were stained with H&E and specialized histological staining, such as Masson staining for cardiac fibrosis evaluation; resorcin-fuchsin staining for lung elastic fiber deposition; periodic acid-silver methamine for glomerular basement membrane assessment; Luxol fast blue staining for brain demyelination; Sirius red staining for hepatic collagen analysis; toluidine blue staining for splenic mast cells; and Phloxine B staining for Paneth cells in the intestine.

For the immunohistochemical analysis, sections were subjected to antigen retrieval and endogenous peroxidase blocking before being blocked for 30 min in normal goat serum (ZSGB-Bio). The sections were then incubated with primary antibodies against Ki67 (Proteintech, 1:1000), 4-HNE (Abcam, 1:200), cleaved caspase-3 (Asp175) (CST, 1:400), and FDX1 (Proteintech, 1:200). Antibody binding was detected using (HRP)-conjugated anti-rabbit/mouse IgG (ZSGB-Bio) and antigen signals were visualized by DAB staining (ZSGB-Bio). The nuclei were counterstained with hematoxylin solution (Beyotime) and the slides were imaged using an Axio Scope A1 microscope (Zeiss).

Statistical analysis

Statistical analyses were performed using GraphPad Prism (version 8) and included two-tailed Student's t-test, and one-way analysis of variance (ANOVA) or two-way ANOVA. Data are presented as mean ± standard deviation (SD), with statistical significance indicated by * $P < 0.05$, ** $P < 0.01$, and *** $P < 0.001$, and ns denotes no significance. Cell culture experiments were independently replicated at least thrice, with a minimum of five animals per experimental group. Further statistical information is provided in the corresponding figure legends.

References

[1] a)G. Kresse, J. Furthmüller, *Computational Materials Science* **1996**, 6, 15; b)G. Kresse, J. Furthmuller, *Phys Rev B Condens Matter* **1996**, 54, 11169; c)G. Kresse, D. Joubert, *Physical Review B* **1999**, 59, 1758.

[2] P. E. Blöchl, *Physical Review B* **1994**, 50, 17953.

[3] J. P. Perdew, K. Burke, M. Ernzerhof, *Phys Rev Lett* **1996**, 77, 3865.

[4] M. Methfessel, A. T. Paxton, *Phys Rev B Condens Matter* **1989**, 40, 3616.

[5] H. J. Monkhorst, J. D. Pack, *Physical Review B* **1976**, 13, 5188.

[6] a)E. Driehuis, K. Kretzschmar, H. Clevers, *Nat Protoc* **2020**, 15, 3380; b)J. F. Dekkers, M. Alieva, L. M. Wellens, H. C. R. Ariese, P. R. Jamieson, A. M. Vonk, G. D. Amatngalim, H. Hu, K. C. Oost, H. J. G. Snippert, J. M. Beekman, E. J. Wehrens, J. E. Visvader, H. Clevers, A. C. Rios, *Nat Protoc* **2019**, 14, 1756; c)T. Sato, D. E. Stange, M. Ferrante, R. G. Vries, J. H. Van Es, S. Van den Brink, W. J. Van Houdt, A. Pronk, J. Van Gorp, P. D. Siersema, H. Clevers, *Gastroenterology* **2011**, 141, 1762; d)J. Puschhof, C. Pleguezuelos-Manzano, A. Martinez-Silgado, N. Akkerman, A. Saftien, C. Boot, A. de Waal, J. Beumer, D. Dutta, I. Heo, H. Clevers, *Nat Protoc* **2021**, 16, 4633.

[7] a)S. K. Shukla, V. Purohit, K. Mehla, V. Gunda, N. V. Chaika, E. Vernucci, R. J. King, J. Abrego, G. D. Goode, A. Dasgupta, A. L. Illies, T. Gebregiworgis, B. Dai, J. J. Augustine, D. Murthy, K. S. Attri, O. Mashadova, P. M. Grandgenett, R. Powers, Q. P. Ly, A. J. Lazenby, J. L. Grem, F. Yu, J. M. Mates, J. M. Asara, J. W. Kim, J. H. Hankins, C. Weekes, M. A. Hollingsworth, N. J. Serkova, A. R. Sasson, J. B. Fleming, J. M. Oliveto, C. A. Lyssiotis, L. C. Cantley, L. Berim, P. K. Singh, *Cancer Cell* **2017**, 32, 71; b)S. Dong, S. Liang, Z. Cheng, X. Zhang, L. Luo, L. Li, W. Zhang, S. Li, Q. Xu, M. Zhong, J. Zhu, G. Zhang, S. Hu, *J Exp Clin Cancer Res* **2022**, 41, 15.

[8] R. Scherz-Shouval, Z. Elazar, *Trends Cell Biol* **2007**, 17, 422.

[9] X. Bai, Y. Huang, M. Lu, D. Yang, *Angew Chem Int Ed Engl* **2017**, 56, 12873.

[10] J. J. Hu, N. K. Wong, S. Ye, X. Chen, M. Y. Lu, A. Q. Zhao, Y. Guo, A. C. Ma, A. Y. Leung, J. Shen, D. Yang, *J Am Chem Soc* **2015**, 137, 6837.

[11] E. F. Pettersen, T. D. Goddard, C. C. Huang, G. S. Couch, D. M. Greenblatt, E. C. Meng, T. E. Ferrin, *J Comput Chem* **2004**, 25, 1605.

[12] R. Anandakrishnan, B. Aguilar, A. V. Onufriev, *Nucleic Acids Res* **2012**, 40, W537.

[13] A. Jakalian, D. B. Jack, C. I. Bayly, *J Comput Chem* **2002**, 23, 1623.

[14] D. S. Goodsell, G. M. Morris, A. J. J. J. o. M. R. Olson, **1996**, 9.

Supporting Figures

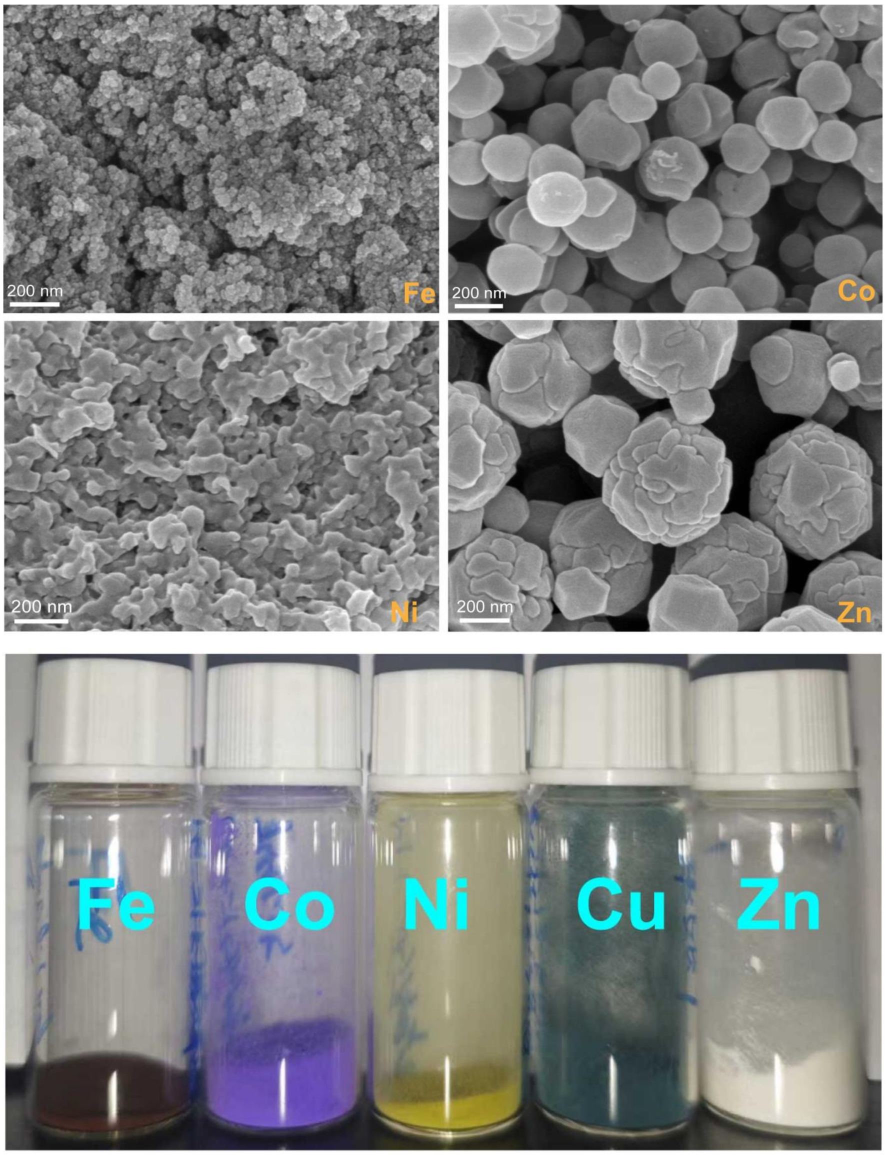

Figure S1. SEM images and digital photograph of M-PrIm (Fe-PrIm, Co-PrIm, Ni-PrIm and Zn-PrIm).

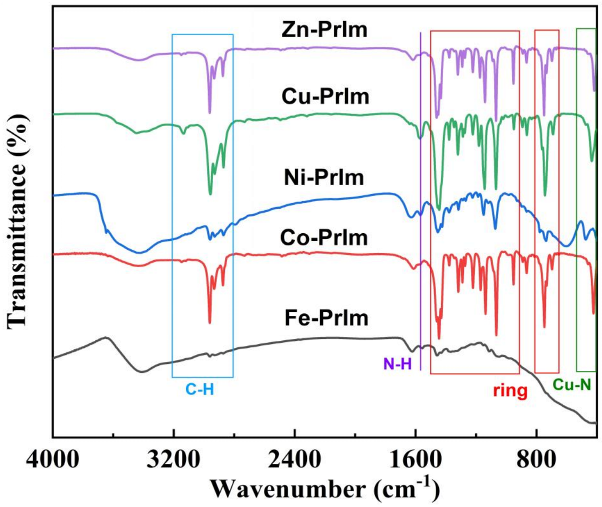

Figure S2. FT-IR spectra of M-PrIm.

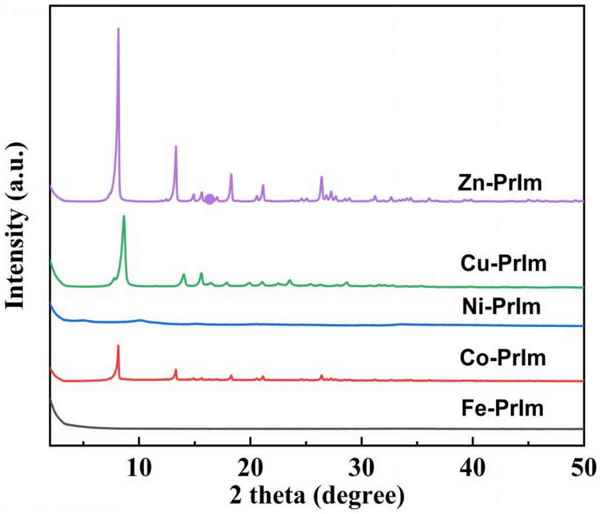

Figure S3. XRD patterns of M-PrIm.

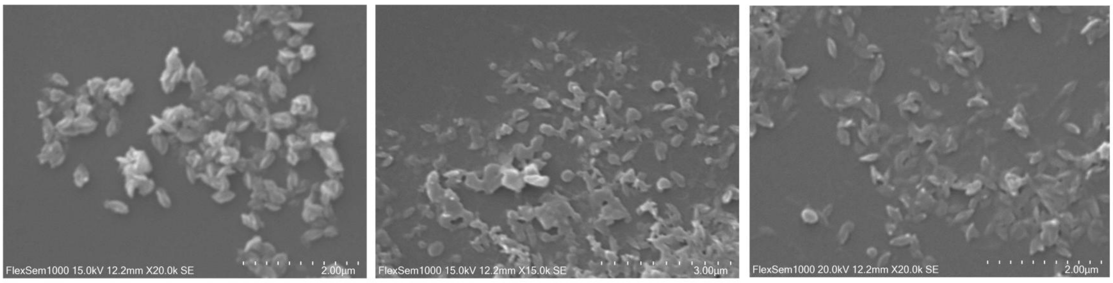

Figure S4. SEM images of Cu-MeIm, Cu-EtIm and Cu-BuIm.

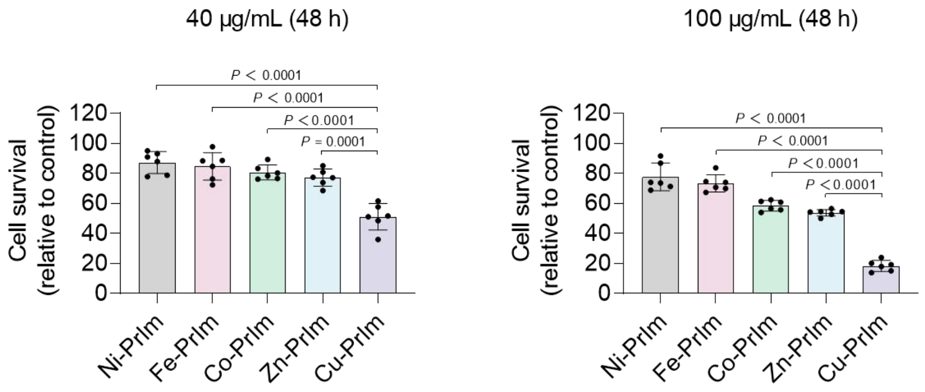

Figure S5. Viability of RKO cells after exposure to 40 µg/mL or 100 µg/mL M-PrIm (Ni-PrIm, Fe-PrIm, Co-PrIm, Zn-PrIm and Cu-PrIm) for 48 hours.

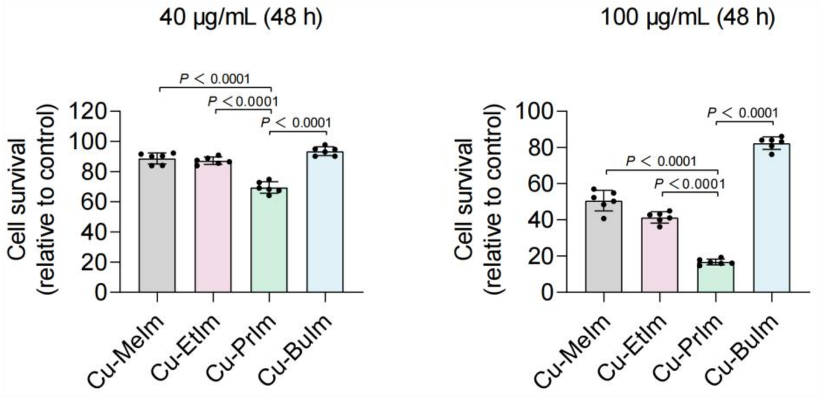

Figure S6. Viability of RKO cells after exposure to 40 μg/mL or 100 μg/mL Cu-based nanozymes (Cu-MeIm, Cu-EtIm, Cu-PrIm, and Cu-BuIm) for 48 hours.

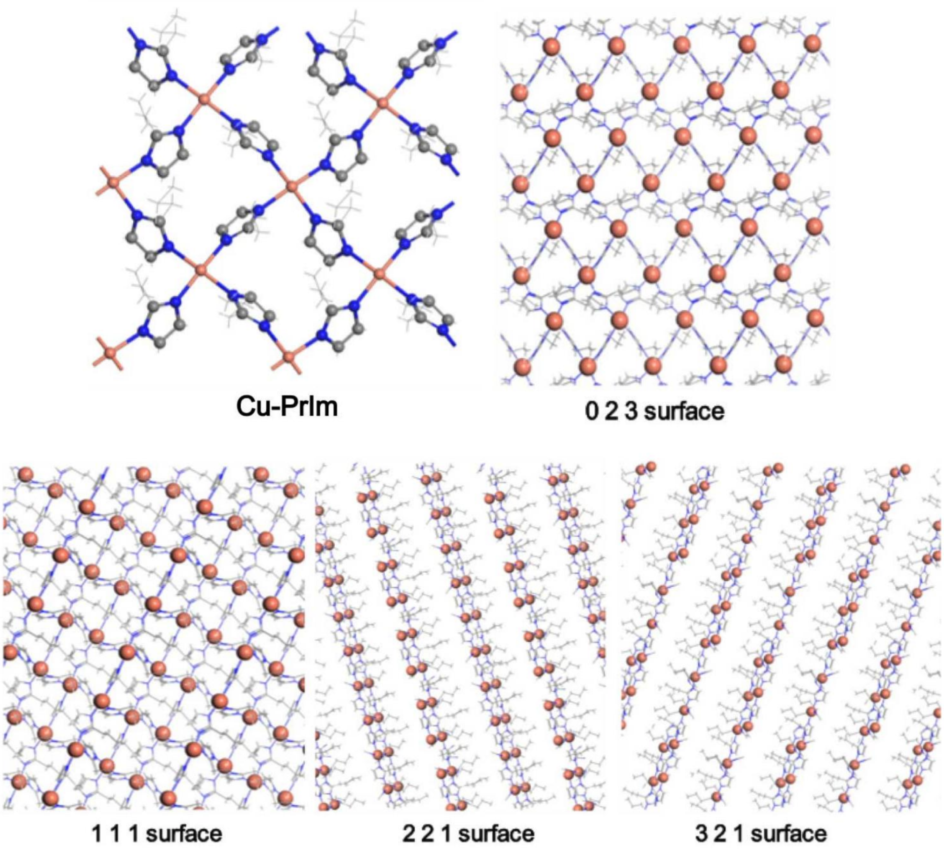

Figure S7. Simulated structure models of Cu-PrIm nanozymes and its corresponding (023), (111), (221) and (321) surface.

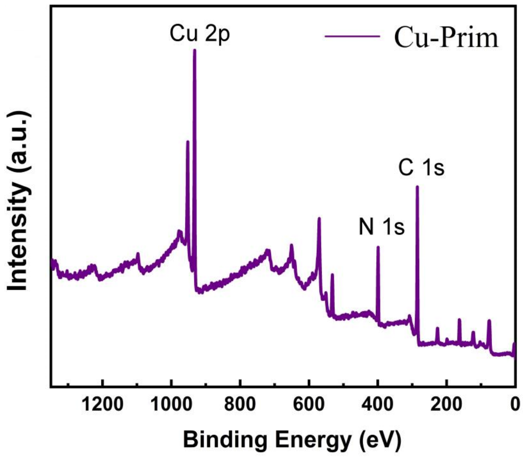

Figure S8. XPS survey spectra of Cu-PrIm nanozymes.

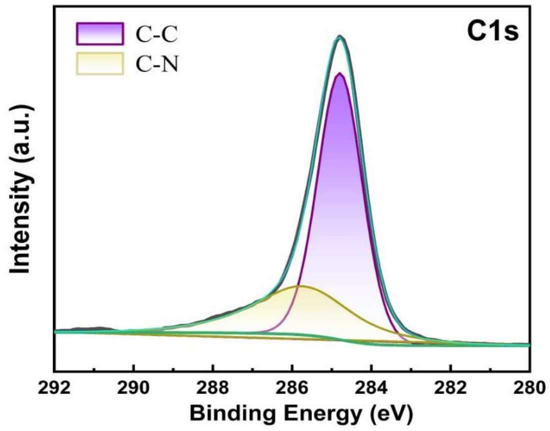

Figure S9. C1s XPS high-resolution scan of Cu-PrIm nanozymes.

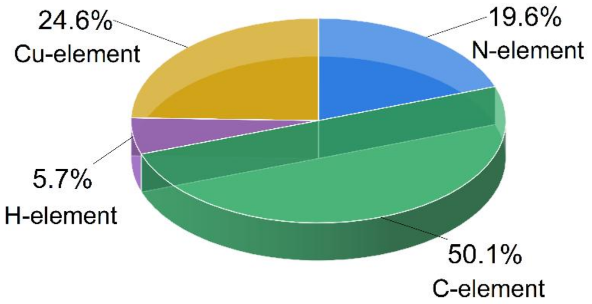

Figure S10. Elemental mass ration in Cu-PrIm nanozymes obtained from ICP-OES and OEA analysis.

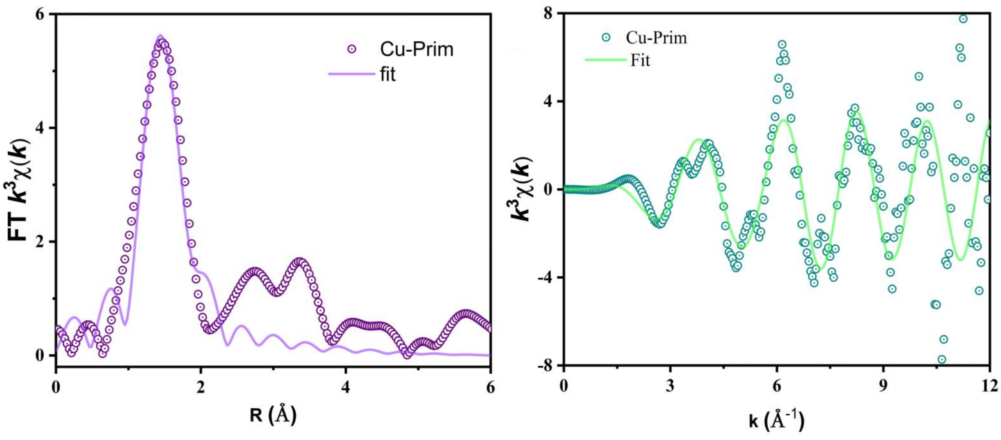

Figure S11. EXAFS spectrum and fitting result in R space (left) and k space (right) at Cu K-edge for Cu-PrIm nanozymes.

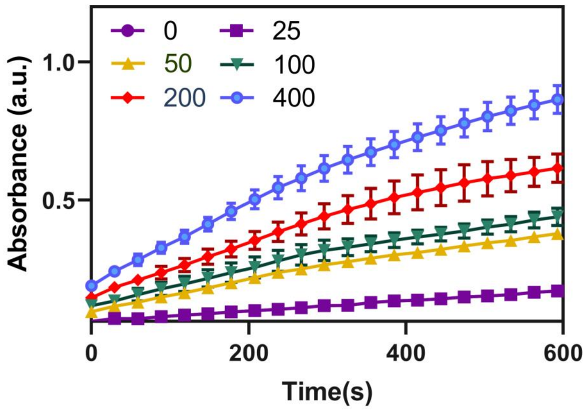

Figure S12. Absorbance changes at 652 nm as a result of TMB oxidation with the change of Cu-PrIm concentration (0, 25, 50, 100, 200, 400 μg/mL).

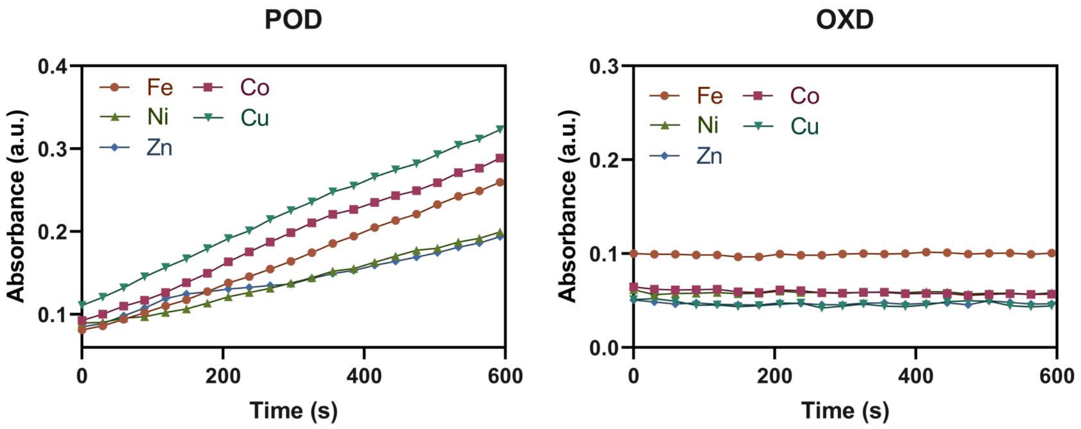

Figure S13. POD and OXD-like activity of M-PrIm.

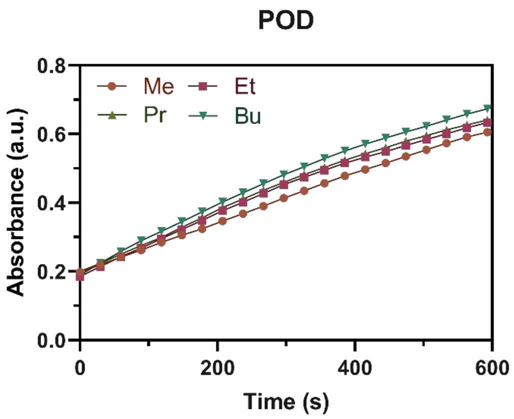

Figure S14. POD-like activity of Cu-MeIm, Cu-EtIm, Cu-PrIm and Cu-BuIm.

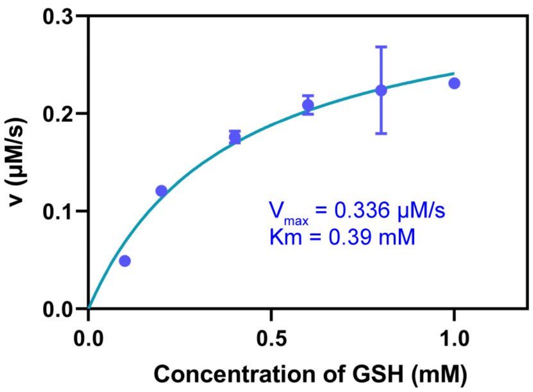

Figure S15. Kinetic curve for the GSHOx activity of Cu-PrIm nanozymes with GSH as substrate.

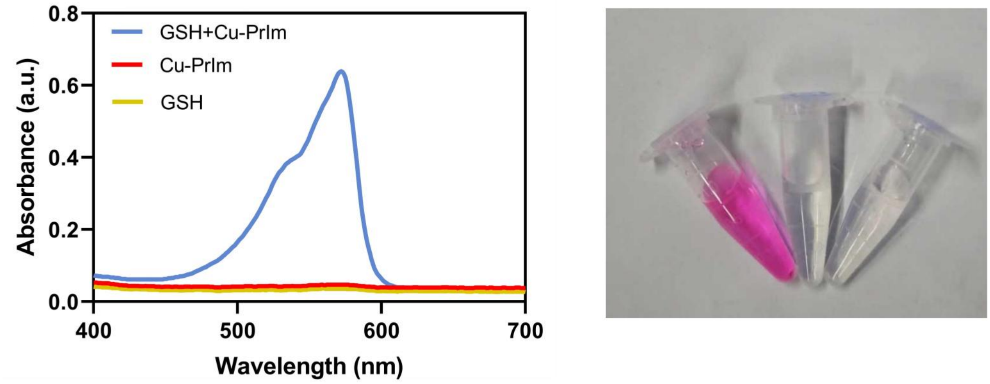

Figure S16. Absorbance and color change of Amplex Red probe incubated with Cu-PrIm nanozymes and GSH for H_2O_2 detection in GSHOx reaction.

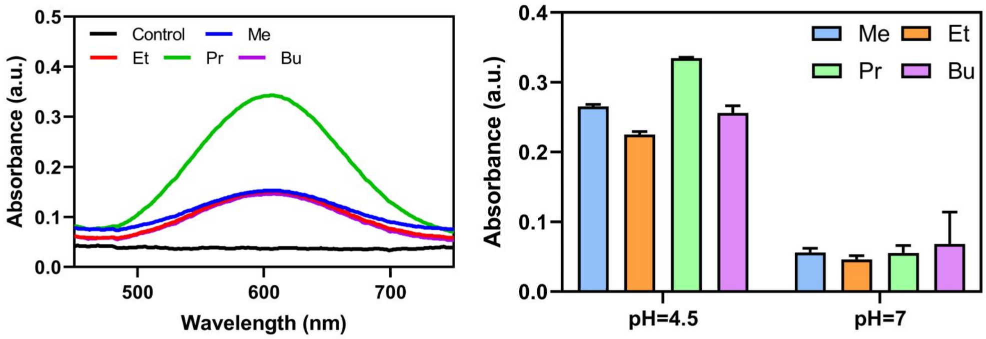

Figure S17. Absorbance of Cu^{2+} release of Cu-MeIm, Cu-EtIm, Cu-PrIm and Cu-BuIm detected by cuprizone as the probe in different pH values.

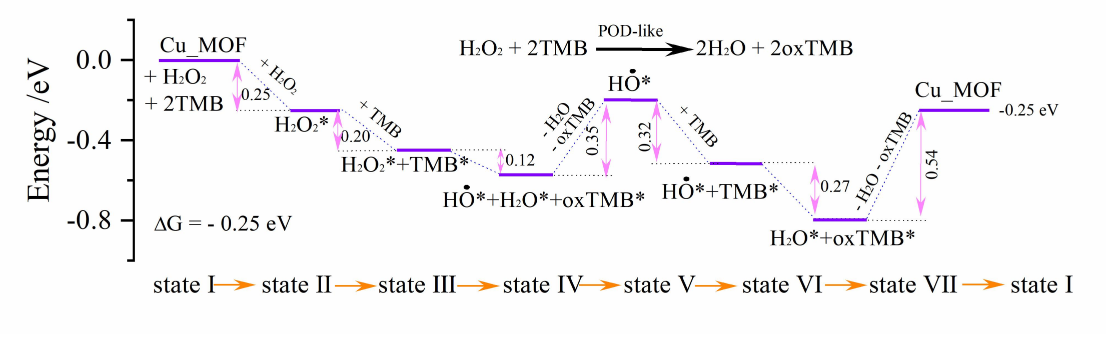

Figure S18. Gibbs free energy profile for a POD-like catalytic cycle of Cu-PrIm nanozymes.

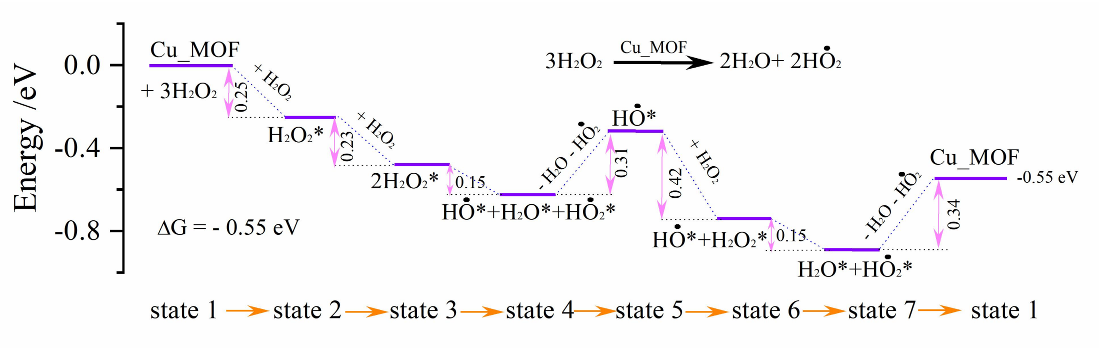

Figure S19. Gibbs free energy profile for a catalytic cycle from H2O2 to HO2• on Cu-PrIm nanozymes.

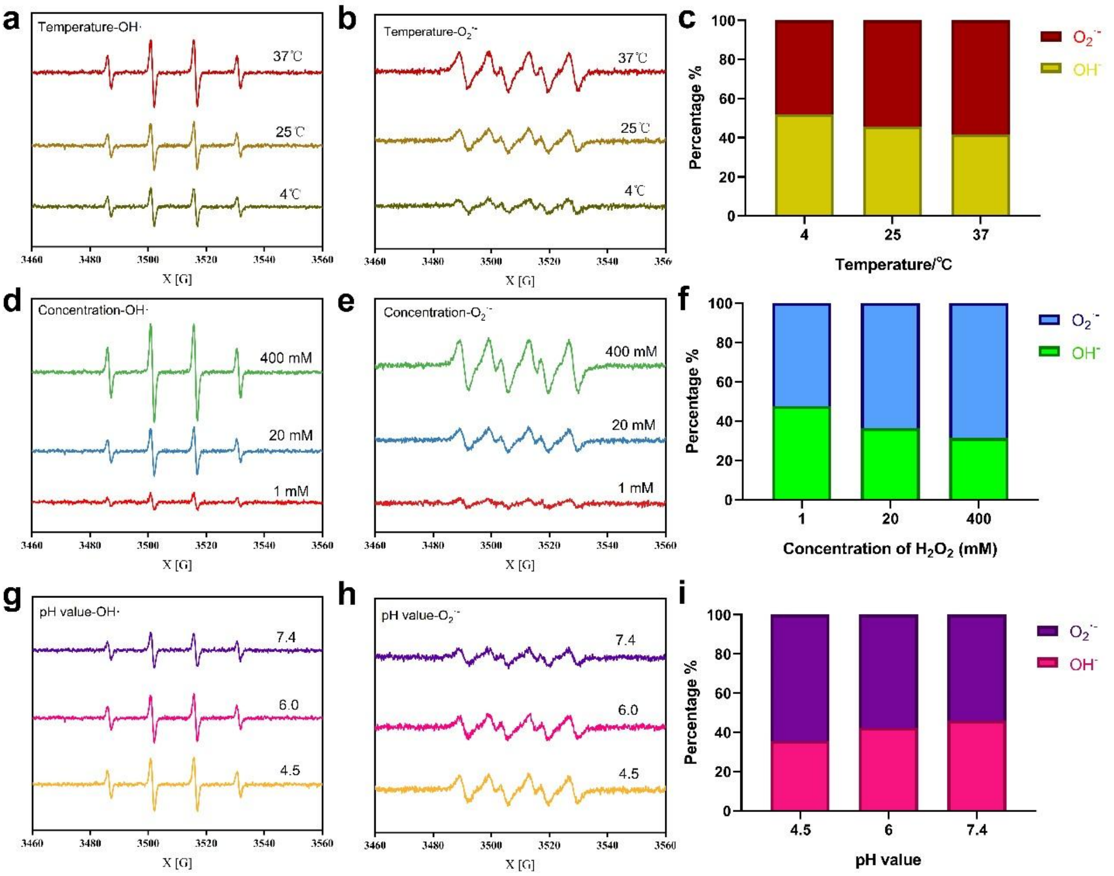

Figure S20. The ESR spectra of a) •OH and b) $•O_2^-$ radicals at 4, 25 and 37°C. c) Proportion of •OH and $•O_2^-$ radicals at different temperature calculated from the relative area of ESR signals. The ESR spectra of d) •OH and e) $•O_2^-$ radicals at H_2O_2 concentrations of 1, 20 and 400 mM. f) Proportion of •OH and $•O_2^-$ radicals at different H2O2 concentrations. The ESR spectra of g) •OH and h) $•O_2^-$ radicals at pH values of 4.5, 6 and 7.4. i) Proportion of •OH and $•O_2^-$ radicals at different pH values.

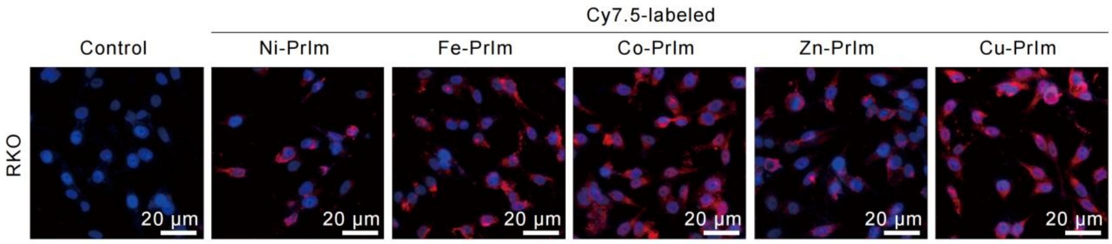

Figure S21. Intracellular uptake of Cy7.5-labeled nanozymes (red) in RKO cells after treatment with 40 μg/mL nanozymes for 24 hours. Scale bar = 20 μm.

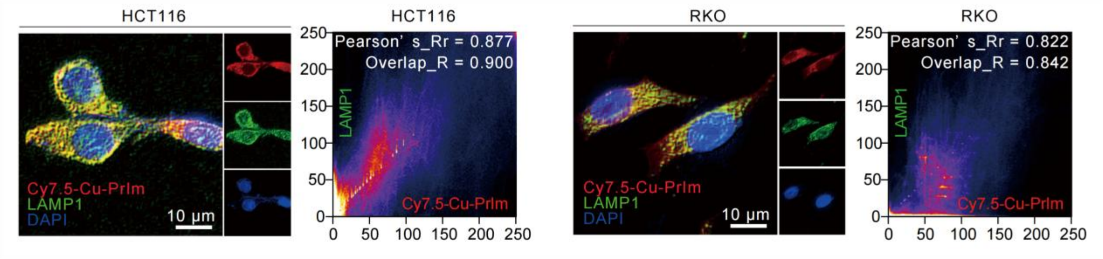

Figure S22. Representative fluorescence images of HCT116 and RKO cells showing co-localization of Cy7.5-labeled Cu-PrIm (red) and the lysosomal marker LAMP1 (green). Scale bar = 10 μm. The extent of co-localization of Cy7.5 and LAMP1 was measured based on Pearson correlation coefficients.

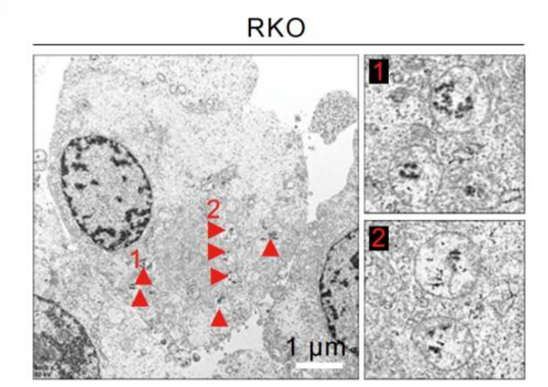

Figure S23. Representative TEM images of RKO cells after 40 µg/mL Cu-PrIm nanozymes treatment for 24 hours. Scale bar = 1 µm.

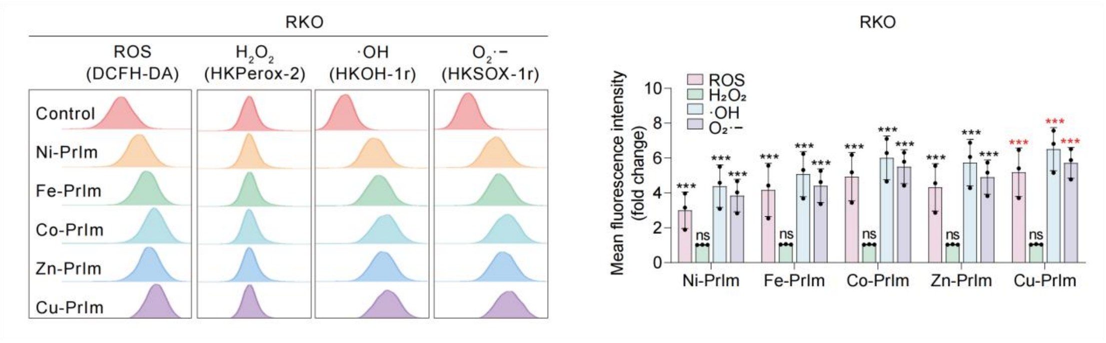

Figure S24. Levels of total ROS and specific ROS species in RKO cells after exposure to 20 μg/mL nanozymes for 24 hours. ****P* < 0.001, or ns = not significant.

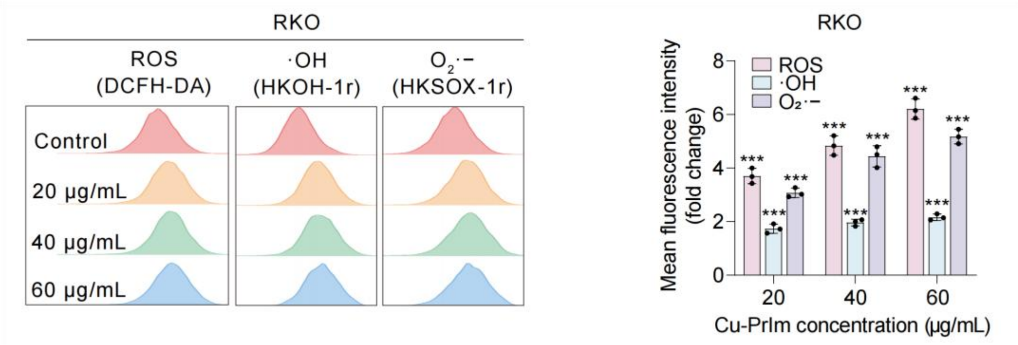

Figure S25. Levels of total ROS and specific ROS species in RKO cells after exposure to different concentrations of Cu-PrIm nanozymes for 24 hours.

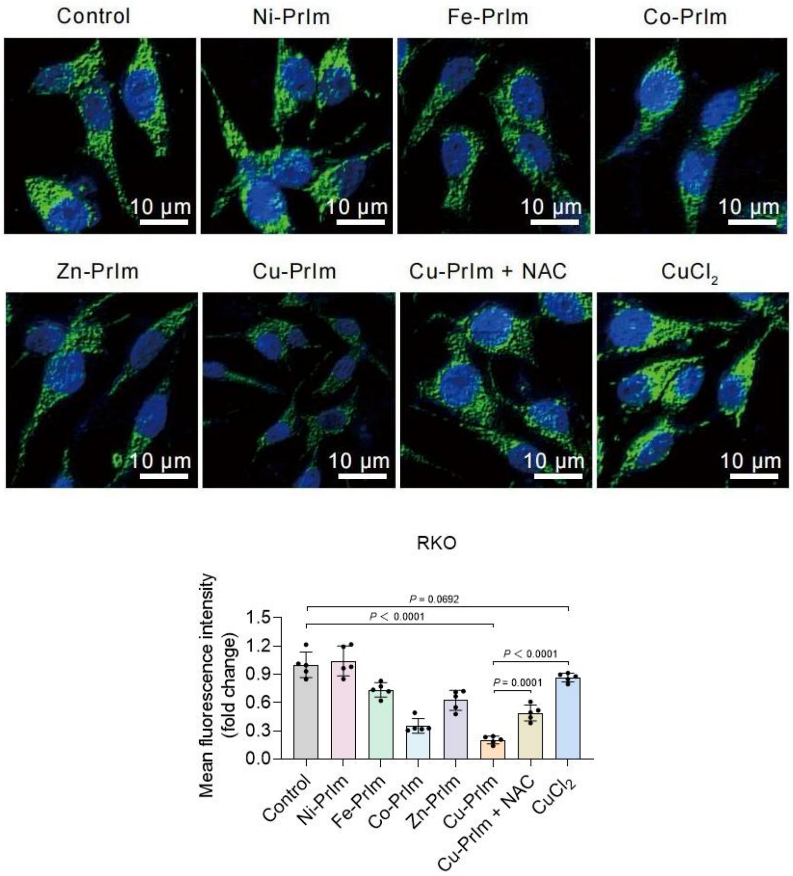

Figure S26. Representative fluorescence images and statistical graph of MitoTracker Green probe (green) in RKO cells after different treatments for 24 hours. Scale bar = 10 μm.

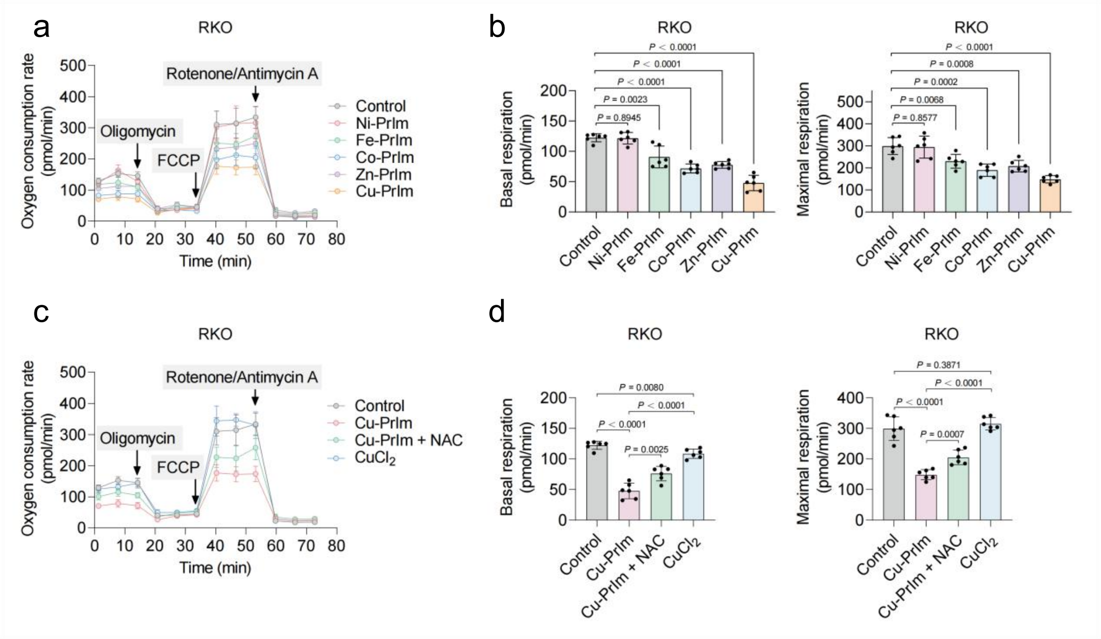

Figure S27. (a) OCR after pretreatment with 40 μg/mL nanozymes for 24 hours, followed by sequential exposure to oligomycin (10 μM), FCCP (15 μM), and rotenone/antimycin A (1 μM and 10 μM, respectively). (b) Basal and maximal respiration of RKO cells after treatment with nanozymes. (c) OCR after pretreatment with 40 μg/mL Cu-PrIm or $CuCl_2$ for 24 hours, followed by sequential exposure to oligomycin (10 μM), FCCP (15 μM), and rotenone/antimycin A (1 μM and 10 μM, respectively). (d) Basal and maximal respiration of RKO cells after treatment with Cu-PrIm or $CuCl_2$.

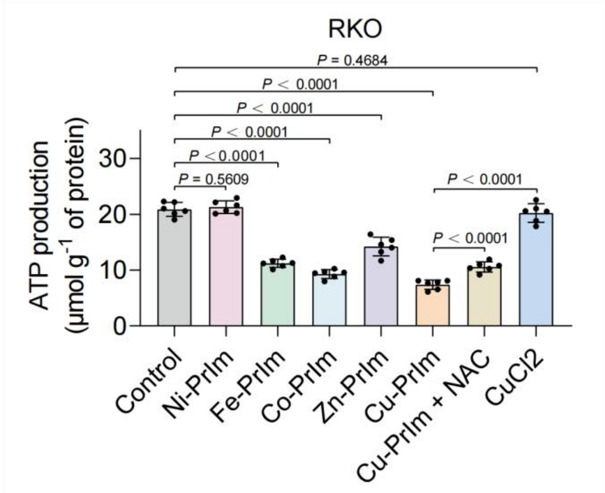

Figure S28. ATP production in RKO cells after treatment with 40 μg/mL nanozymes or $CuCl_2$ for 24 hours.

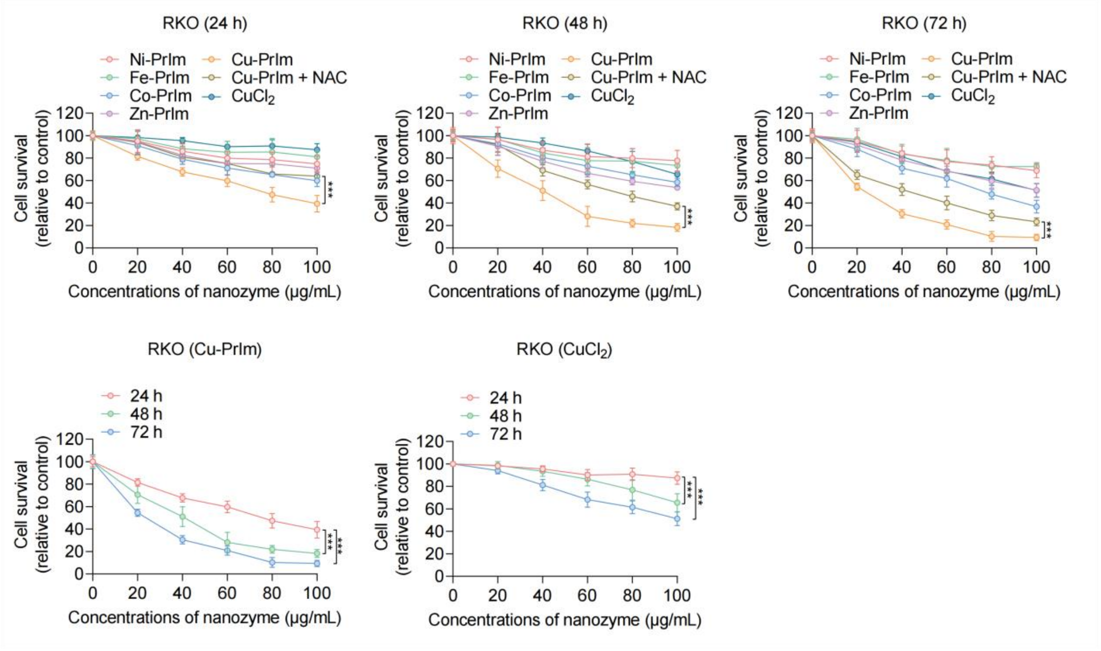

Figure S29. Viability of RKO cells after treatments with various concentrations and times.

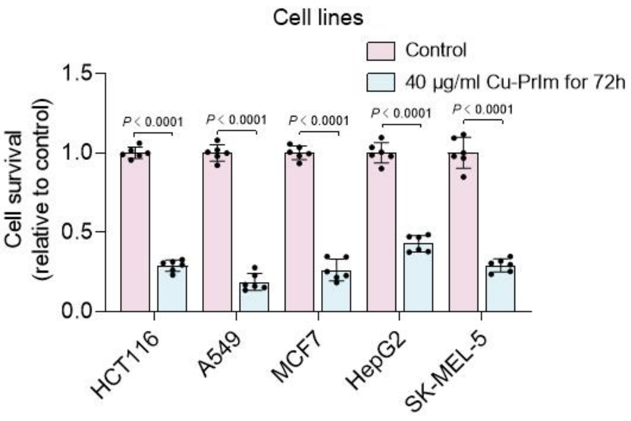

Figure S30. Viability of various cancer cells after Cu-PrIm treatment with 40 μg/ml for 72 h.

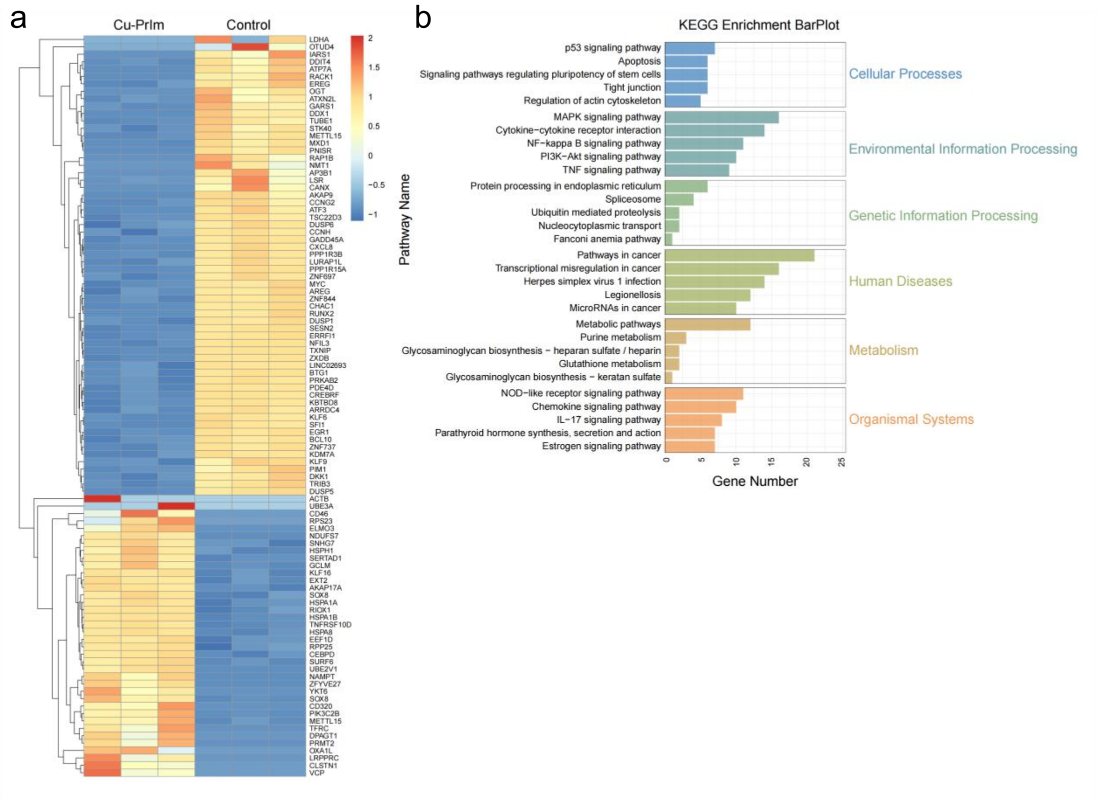

Figure S31. (a) Cluster heat maps of differential gene expression in Cu-PrIm nanozymes treated HCT116 cells versus control. (b) Bar graph showing significantly enriched KEGG pathways.

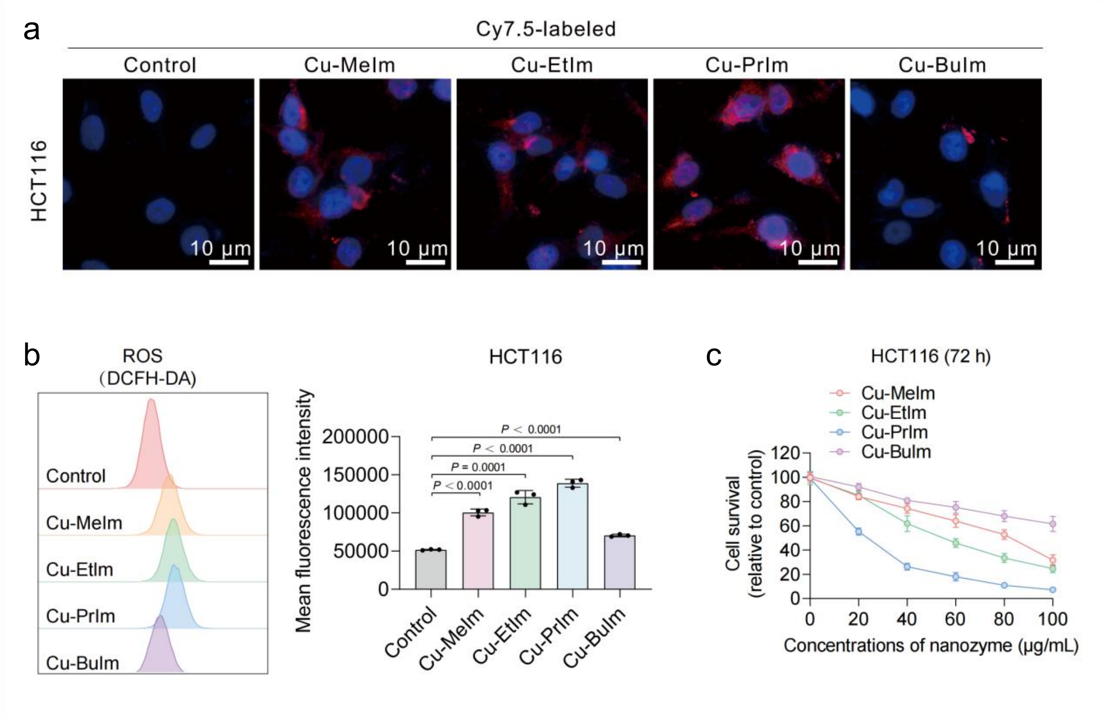

Figure S32. (a) Intracellular uptake of Cy7.5-labeled nanozymes (red) in HCT116 cells after treatment with 40 μg/mL nanozymes for 24 hours. Scale bar = 10 μm. (b) Levels of total ROS in HCT116 cells after exposure to 20 μg/mL nanozymes for 24 hours. (c) Viability of HCT116 cells treated with gradient concentrations of nanozymes for 72 h.

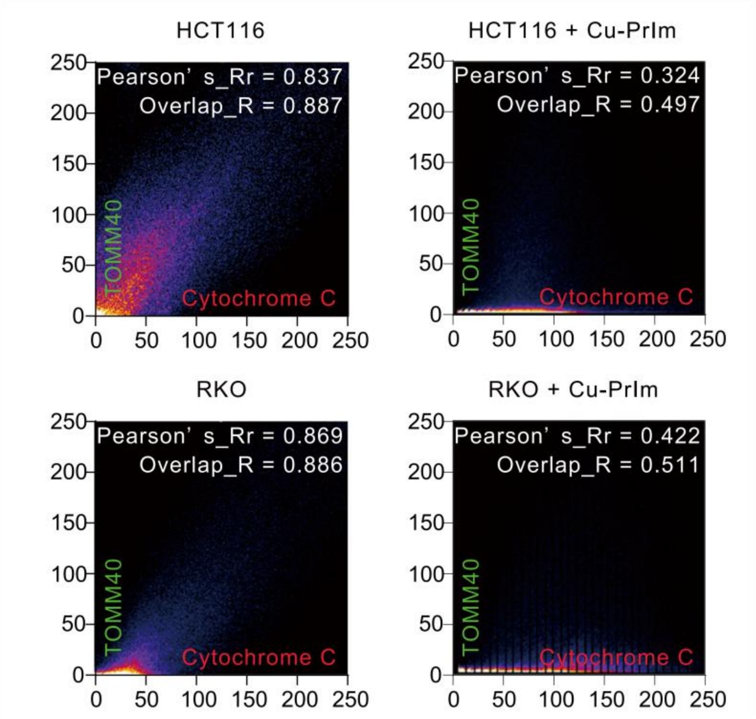

Figure S33. The extent of co-localization of TOMM20 and cytochrome C was measured based on Pearson correlation coefficients.

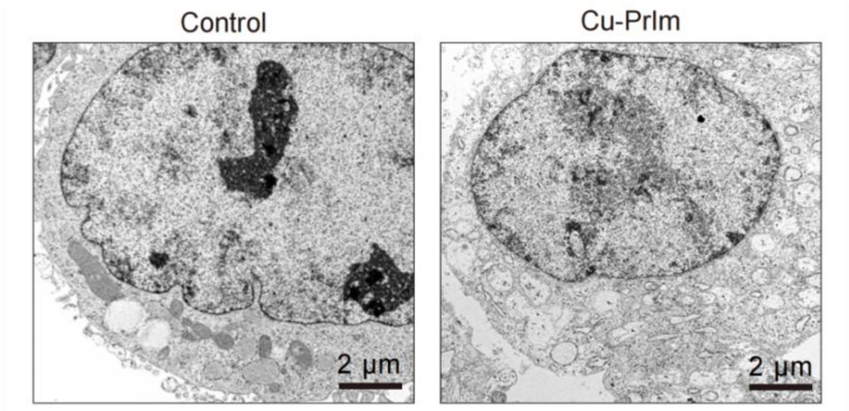

Figure S34. Representative TEM images of HCT116 cells after 40 μg/mL Cu-PrIm treatment for 24 hours. Scale bar = 2 μm.

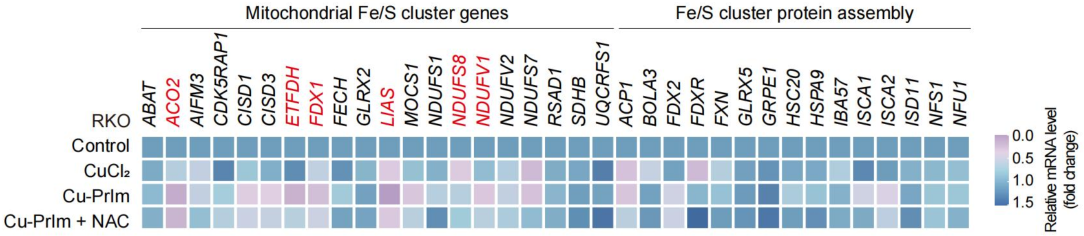

Figure S35. PCR analysis of mitochondrial Fe/S cluster genes and Fe/S cluster protein assembly in RKO cells treated with 20 μg/mL $CuCl_2$, 20 μg/mL Cu-PrIm, or 1 mM NAC for 24 hours. *ACTB* was used as the internal reference.

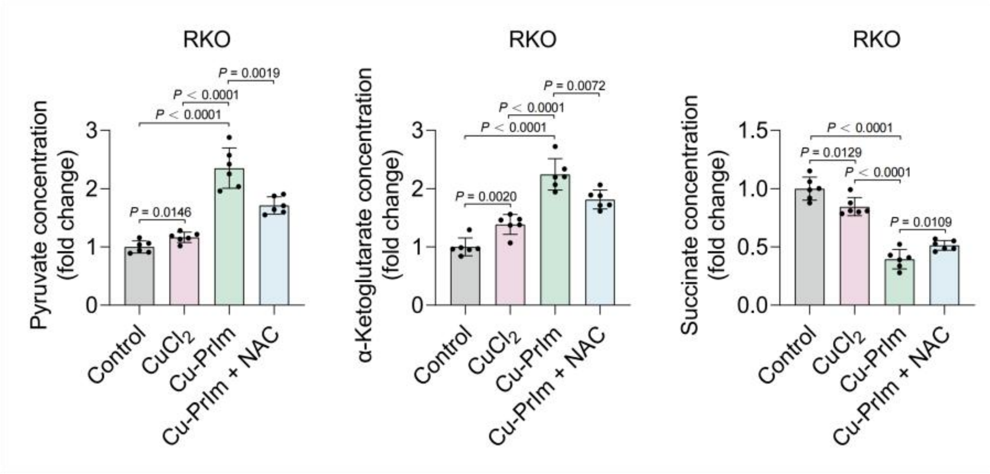

Figure S36. Pyruvate, α-ketoglutarate, and succinate concentrations in RKO cells treated with 20 μg/mL $CuCl_2$, 20 μg/mL Cu-PrIm, or 1 mM NAC for 24 hours.

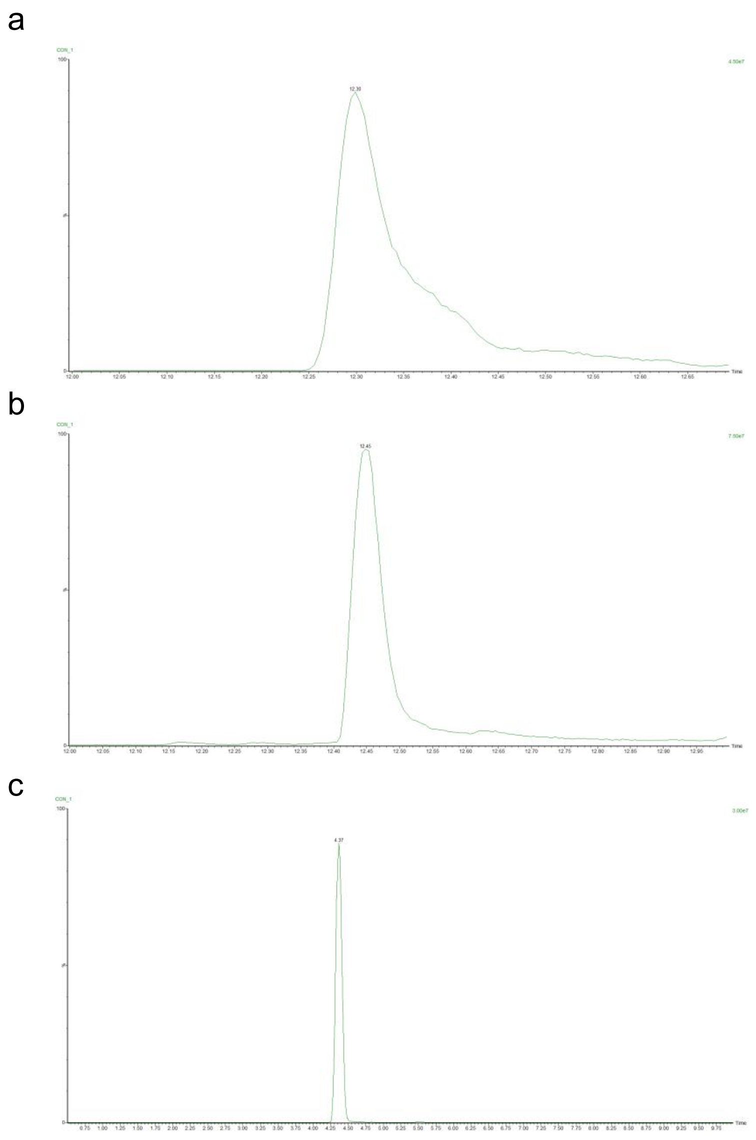

Figure S37. HPLC chromatograms of (a) pyruvate, (b) α-ketoglutarate, and (c) succinate standards.

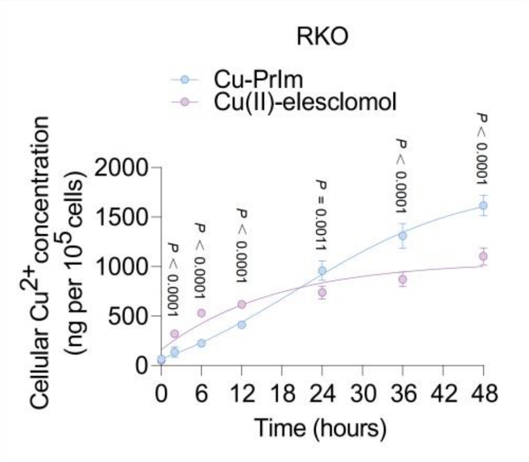

Figure S38. Copper ion concentrations in RKO cells at different time points after treatment with 40 μg/mL Cu-PrIm or Cu(II)-elesclomol.

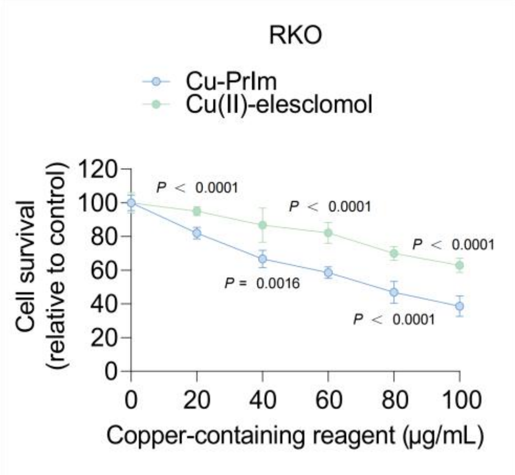

Figure S39. Viability of RKO cells after treatment with Cu-PrIm or Cu(II)-elesclomol at various concentrations for 24 hours.

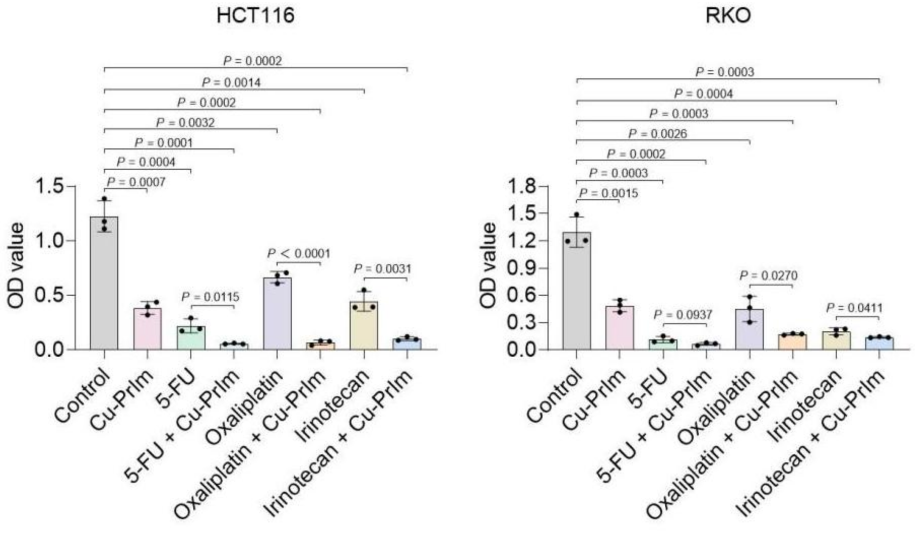

Figure S40. Statistics results of colony formation of HCT116 and RKO cells treated with chemotherapeutic agents (5-Fu, oxaliplatin, and irinotecan) and 20 μg/mL Cu-PrIm.

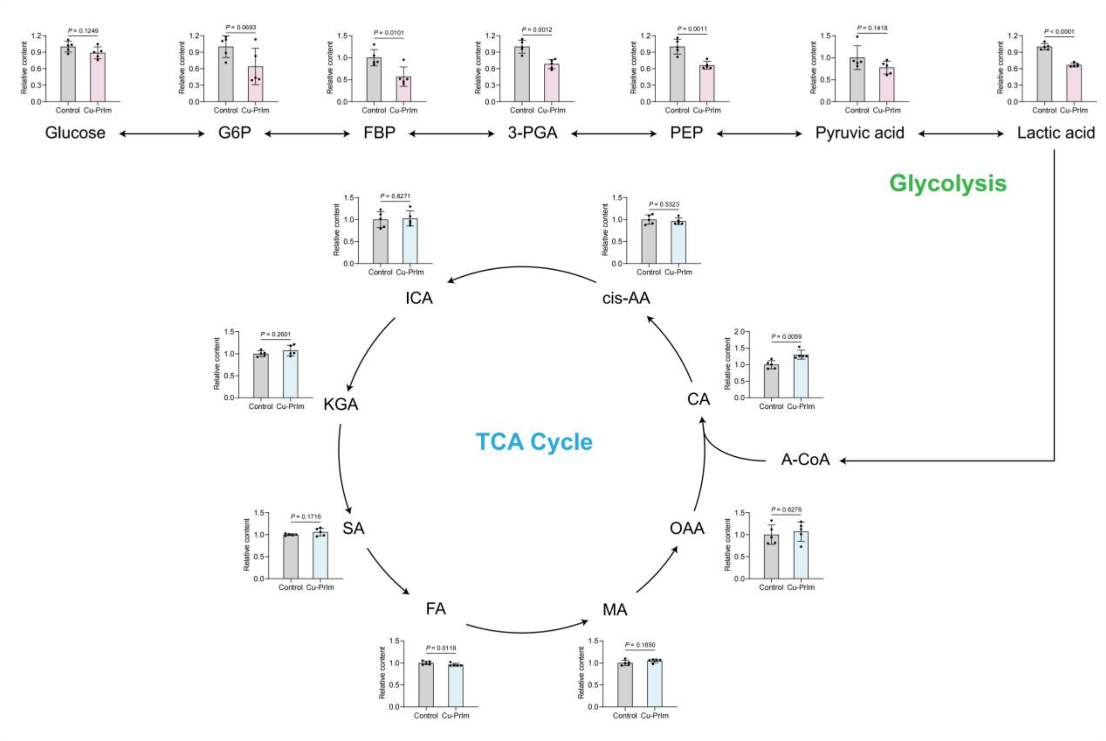

Figure S41. Metabolites altered in the glycolysis and the TCA cycle in Cu-PrIm-treated 5-FU-R HCT116 cells versus control.

a

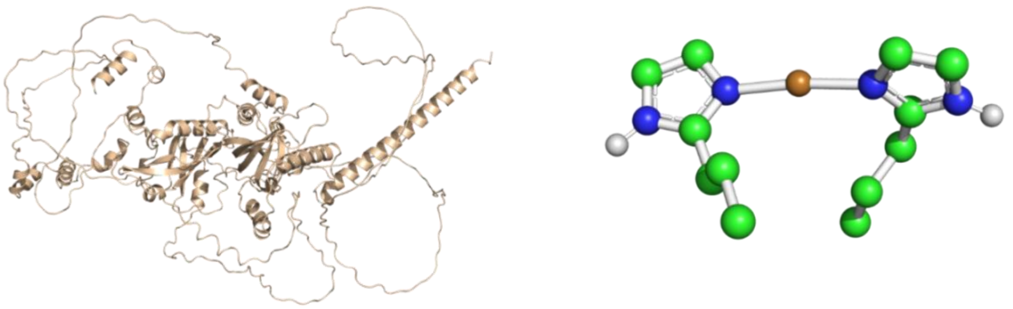

b

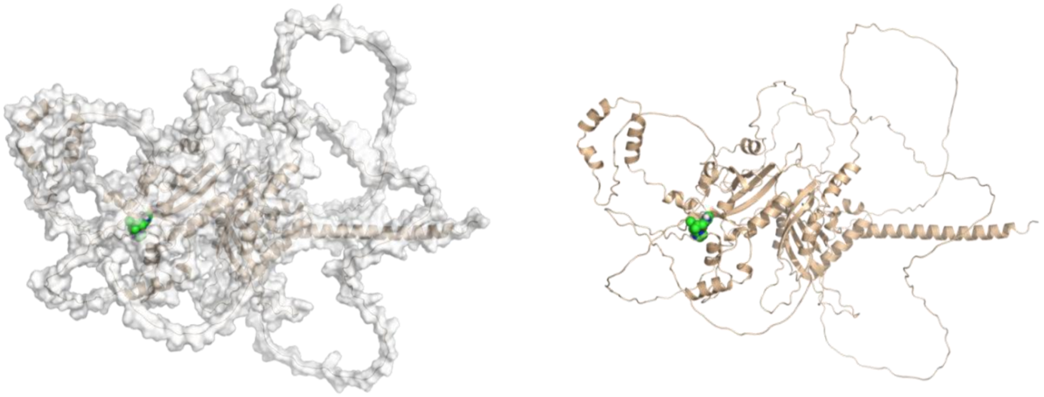

Figure S42. The simulation-based interaction analysis of Cu-PrIm nanozymes and HIF-1α proteins. (a) Structure analysis of this study. (b) Overview of binding mode.

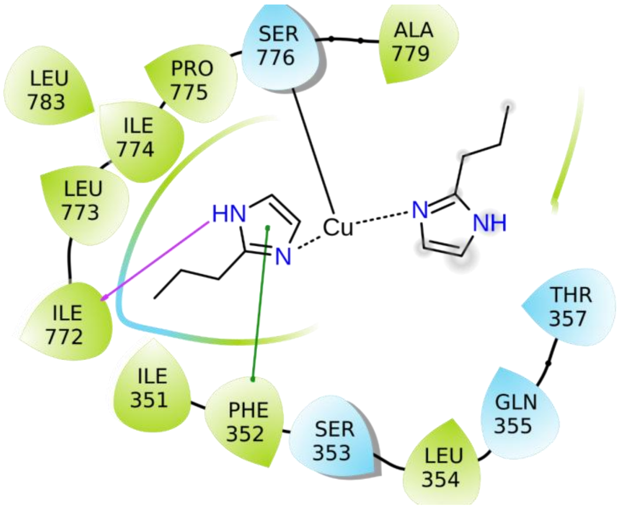

Figure S43. 2D interaction detail display. Acidic amino acids are red, basic are blue, polar are cyan, and hydrophobic are green. Hydrogen bonds are shown with purple arrows, and π-π stacking interactions are shown in green.

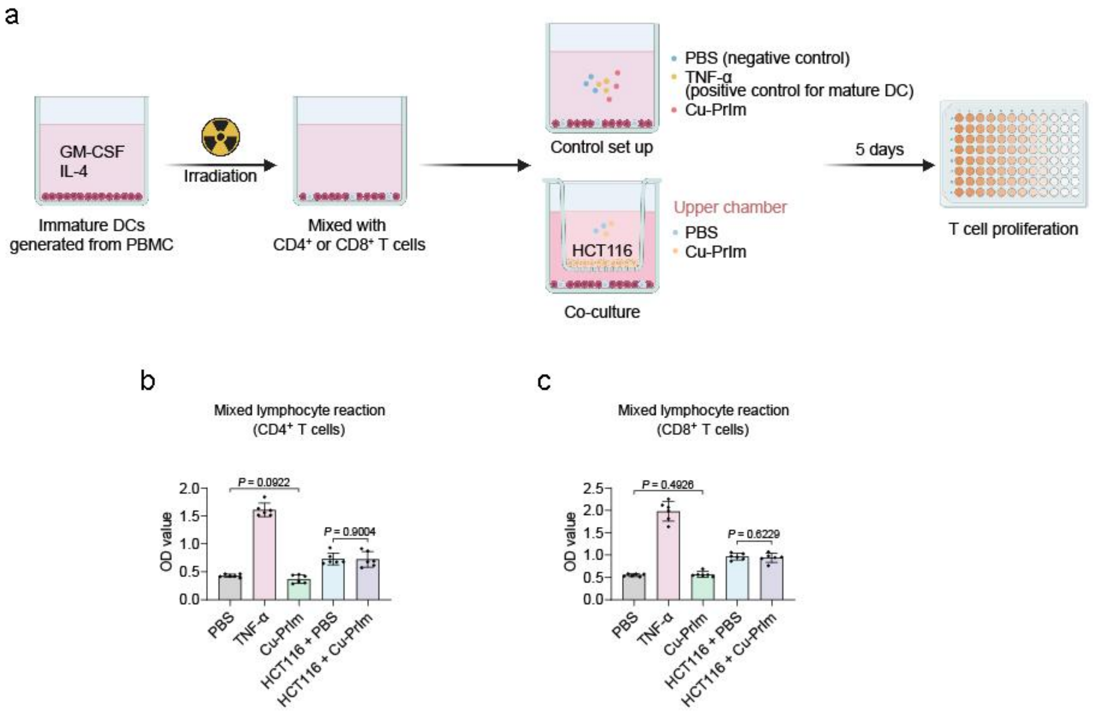

Figure S44. The immunogenicity of Cu-PrIm nanozymes. (a) Schematic diagram of mixed lymphocyte reaction. (b) The proliferation of CD4+ and CD8+ T cells was quantitatively assessed using the CCK-8 assay.

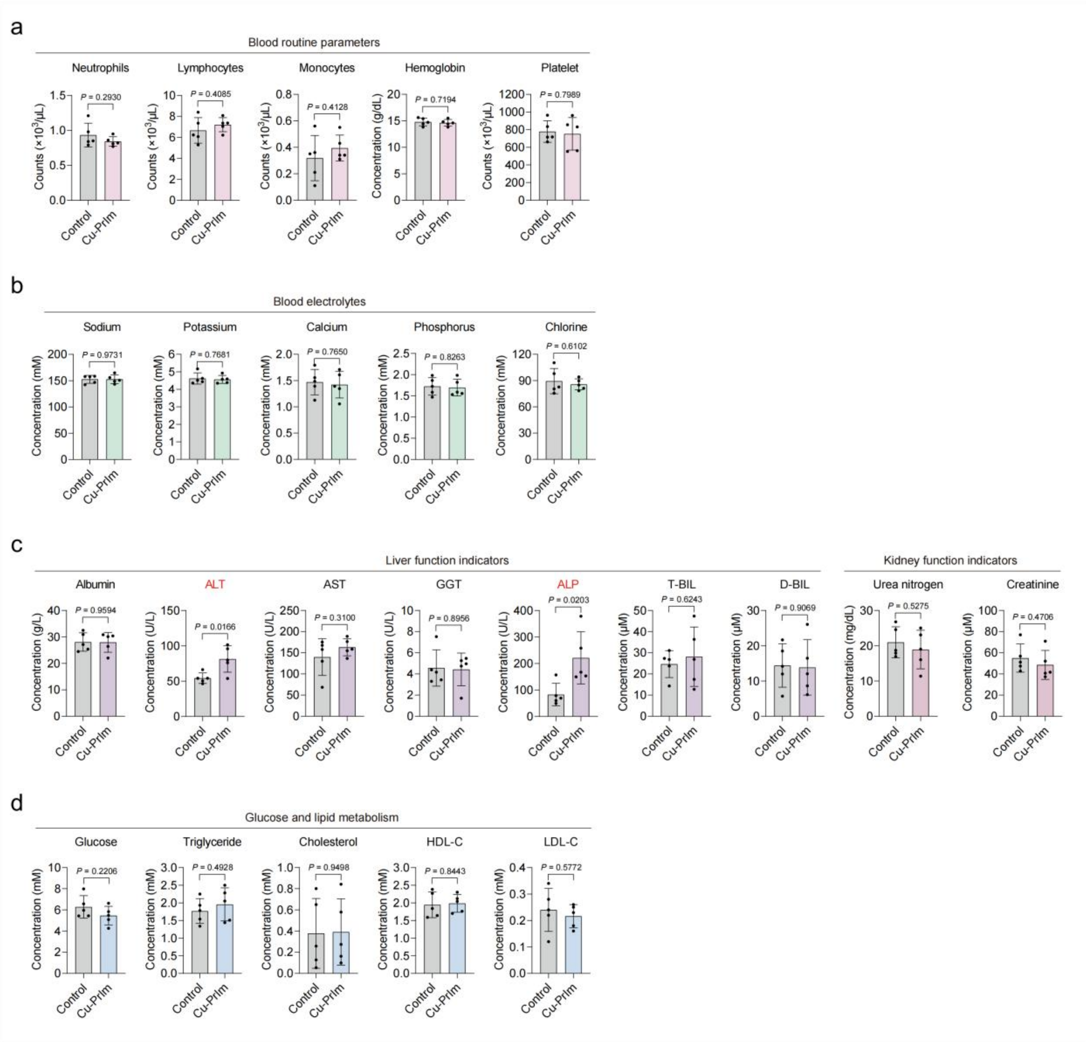

Figure S45. Blood indexes of mice with various treatments, including (a) blood parameters, (b) blood electrolytes, (c) liver and kidney function parameters, and (d) glucose and lipid metabolism indicators.

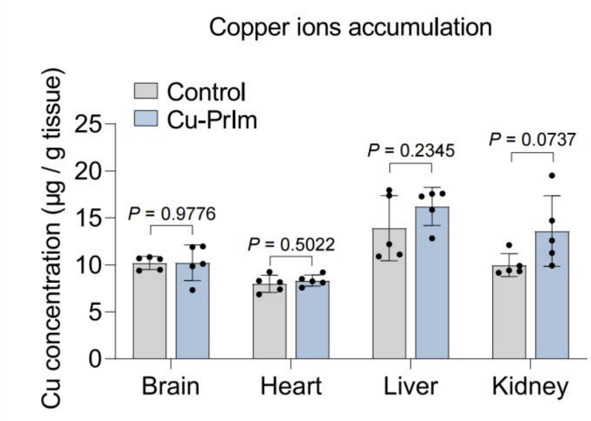

Figure S46. Copper ion concentrations in vital organs 2 weeks after the end of PBS or Cu-PrIm treatment.

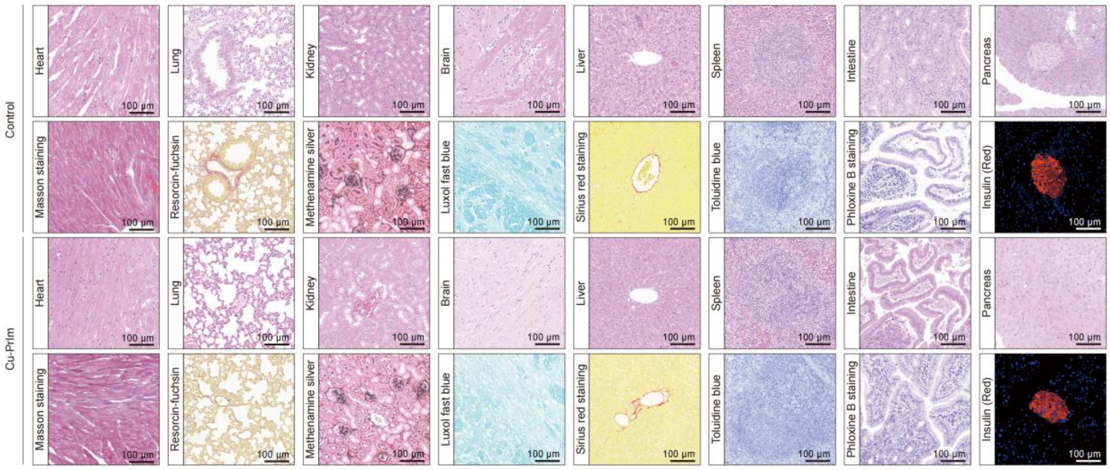

Figure S47. H&E and specialized histological staining of vital organs 2 weeks after the end of PBS or Cu-PrIm treatment. Histological staining including: Masson staining for cardiac fibrosis assessment, resorcin-fuchsin staining for elastic fiber deposition in the lungs, periodic acid-silver metheramine staining for the glomerular basement membrane, Luxol fast blue staining for demyelination in the brain, Sirius red staining for hepatic collagen, Toluidine blue staining for splenic mast cells, Phloxine B staining for Paneth cells in the intestine, and insulin immunofluorescence staining for pancreatic islets. Scale bar = 100 μm.

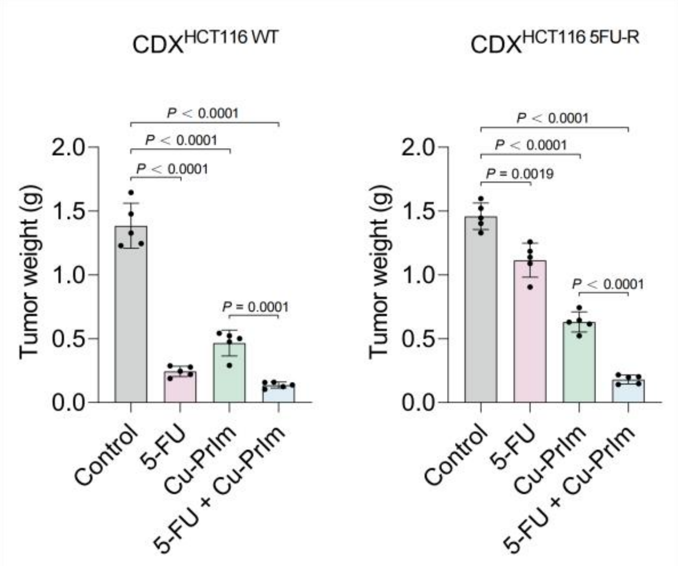

Figure S48. Weight of the dissected tumors from CDX mice at the end of treatment.

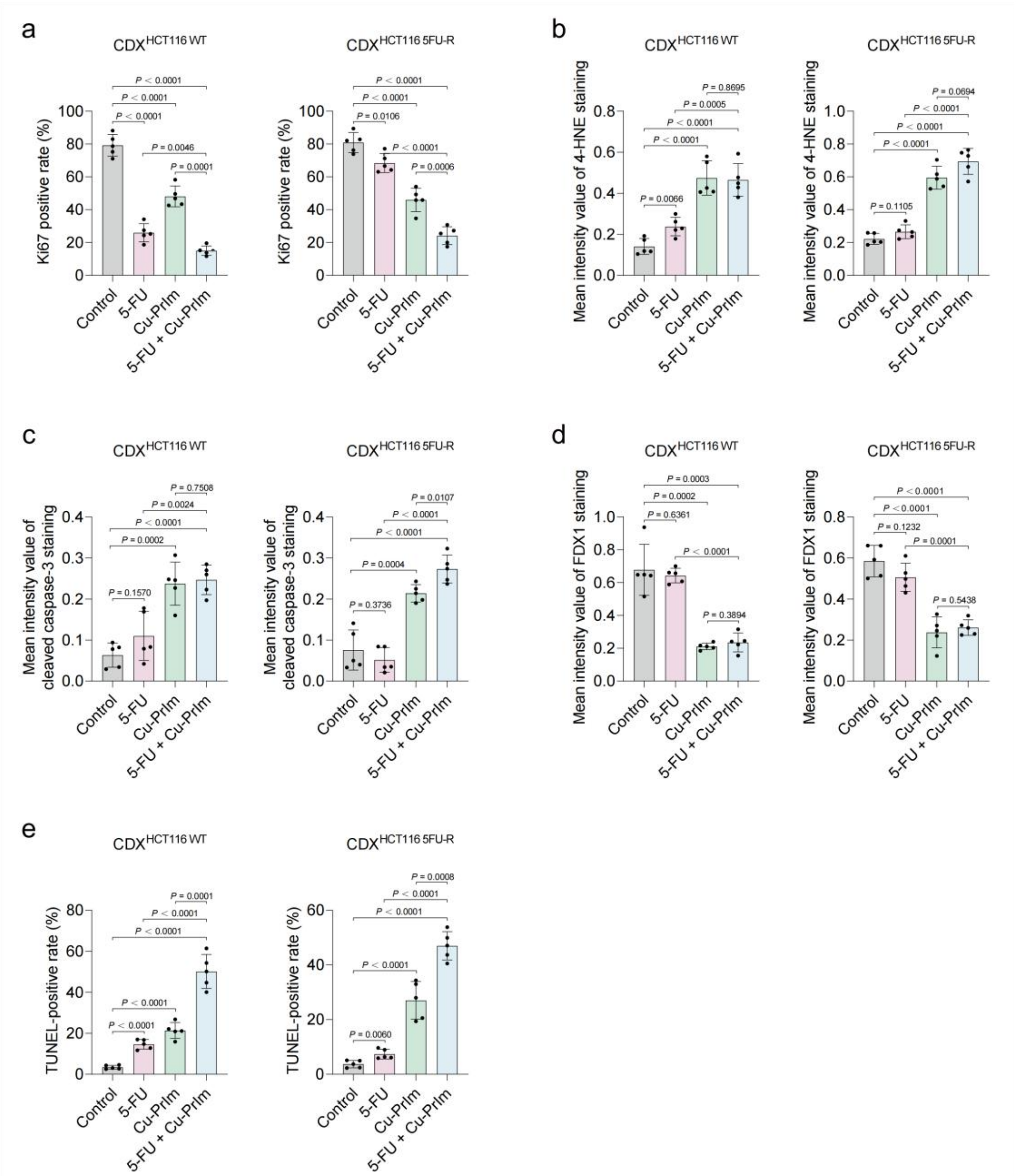

Figure S49. (a) Ki67-positive rate (%) in the indicated xenograft tumors. (b-d) Mean intensity values of (b) 4-HNE, (c) cleaved caspase-3, and (d) FDX1 in the indicated xenograft tumors. (e) TUNEL-positive rate (%) in the indicated xenograft tumors.

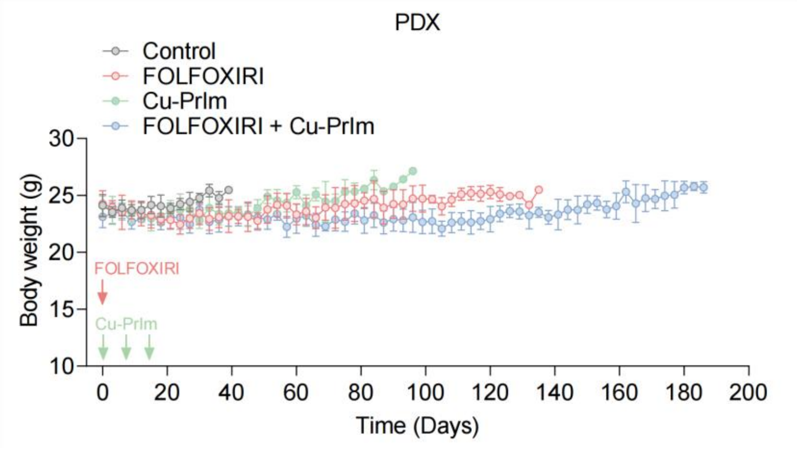

Figure S50. Weight changes of PDX mice treated with a single dose of FOLFOXIRI (10 mg/kg 5-Fu, 20 mg/kg leucovorin, 2 mg/kg oxaliplatin, and 50 mg/kg irinotecan) or 3 doses of Cu-PrIm (10 mg/kg, 1 time per week).

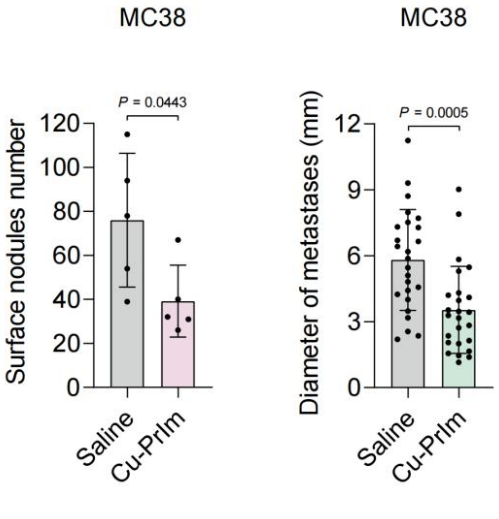

Figure S51. Quantification of metastatic lung nodules after saline or Cu-PrIm treatment.

Supporting Tables

Table S1. Key resources table

Chemicals	Source	Identifier
Cy7.5 NHS ester	MedChemExpress	Cat#HY-D1567
N-Acetylcysteine (NAC)	MedChemExpress	Cat#HY-B0215
Copper(II) chloride ($CuCl_2$)	Sigma-Aldrich	Cat#751944
Docosahexaenoic acid (DHA)	MedChemExpress	Cat#HY-B2167
Necrostatin-1	Selleck	Cat#S8037
Ferrostatin-1	Selleck	Cat#S7243
Liproxstatin-1	Selleck	Cat#S7699
Z-VAD-FMK	Selleck	Cat#S7023
Ac-FLTD-CMK	Selleck	Cat#S9817
Cu(II)-elesclomol	MedChemExpress	Cat#HY-156376
Bax inhibitor peptide V5	MedChemExpress	Cat#HY-P0081
Caspase-9 inhibitor III	MedChemExpress	Cat#HY-136744
Tetrathiomolybdate	Selleck	Cat#E1166
5-Fluorouracil (5-FU)	Abcam	Cat#ab142387
Irinotecan	MedChemExpress	Cat#HY-16562
Oxaliplatin	MedChemExpress	Cat#HY-17371
VHL-IN-1	MedChemExpress	Cat#HY-156106
Cycloheximide	MedChemExpres	Cat#HY-12320
**Reagents**	**Source**	**Identifier**
RPMI-1640 medium	Gibco	Cat#11875119
Fetal bovine serum	Gibco	Cat#A5670701
Penicillin-streptomycin	Gibco	Cat#15140122
0.25% Trypsin-EDTA digestion	Biosharp	Cat#BL512A
Complete medium for liver sinusoidal endothelial cells	Cas9X	Cat#PAHX-G124
Complete medium for pancreatic islet cells	Cas9X	Cat#PXHX-G214
Complete medium for umbilical vein endothelial cells	Cyagen	Cat#HUVEC-90011

MasterAim digestion solution	AimingMed	Cat#100-051; 100-051
Matrigel (Matrix for organoid)	Corning	Cat#356255
Matrigel (Matrix HC)	Corning	Cat#354248
MasterAim CRC organoid medium	AimingMed	Cat#100-067; 100-068
TrypLE express	Gibco	Cat#12604013
Paraformaldehyde	Servicebio	Cat#G1101
DAPI	Abcam	Cat#ab228549
Triton X-100	Servicebio	Cat#GC204003
Mounting medium with DAPI	Abcam	Cat#ab104139
2.5% Glutaraldehyde	Servicebio	Cat#G1102
Osmium acid	Ted Pella	Cat#18456
Uranium acetate	SPI	Cat#02624-AB
Lead citrate	Sigma-Aldrich	Cat#203580
DCFH-DA	MedChemExpress	Cat#HY-D0940
HKPerox-2	MedChemExpress	Cat#HY-D1157
HKOH-1r	MedChemExpress	Cat#HY-D1159
HKSOX-1r	MedChemExpress	Cat#HY-130017
Mito-Tracker Green	Beyotime	Cat#C1048
Hoechst 33342 staining solution	Beyotime	Cat#C1028
RIPA buffer	Beyotime	Cat#P0013B
Protease inhibitors	Epizyme	Cat#GRF101
Phosphatase inhibitors	Epizyme	Cat#GRF102
TRIzol reagent	Thermo Fisher	Cat#15596018CN
Dynabeads oligo $(dT)_{25}$	Thermo Fisher	Cat#61002
SuperScript II reverse transcriptase	Invitrogen	Cat#18064022
DNA polymerase I (E. coli)	NEB	Cat#M0209VVIAL
RNase H	NEB	Cat#M0297S
dUTP solution	Thermo Fisher	Cat#R0133
Goat serum	ZSGB-Bio	Cat#ZLI-9021

0.1% Crystal violet solution	Solarbio	Cat#G1063
DAB staining	ZSGB-Bio	Cat#ZLI-9017
Hematoxylin solution	Beyotime	Cat#C0107
1% Acid alcohol	Beyotime	Cat#C0165
Human GM-CSF (*E.coli*)	Novoprotein	Cat#C003
Human IL-4 (*E.coli*)	PeproTech	Cat#200-04
Human TNF-α (*E.coli*)	Novoprotein	Cat#DC008
**Kits**	**Source**	**Identifier**
Mycoplasma PCR detection kit	Sigma-Aldrich	Cat#MP0050
Cell counting kit-8	MedChemExpress	Cat#HY-K0301
CellTiter-Glo 3D cell viability assay	Promega	Cat#G9681
Seahorse XF Cell Mito stress test kit	Agilent	Cat#103015-100
ATP assay kit	Beyotime	Cat#S0026
BCA protein assay kit	Beyotime	Cat#P0010
TUNEL cell apoptosis detection kit	Beyotime	Cat#C1088
Annexin V-FITC/PI assay	Bestbio	Cat#BB4101
Copper assay kit	Abcam	Cat#ab272528
Human monocyte isolation kit	StemCell	Cat#19359
Human $CD4^+$ T cell enrichment kit	StemCell	Cat#17952
Human $CD8^+$ T cell enrichment kit	StemCell	Cat#17953
10% SDS-PAGE gel	Epizyme	Cat#PG212
ReverTra Ace qPCR RT kit	Toyobo	Cat#FSQ-201
SYBR Green PCR master mix	Toyobo	Cat#QPK-101
**Antibodies**	**Source**	**Identifier**
Rabbit monoclonal anti-Bcl-2	Abcam	Cat#ab182858
Rabbit monoclonal anti-Bax	Abcam	Cat#ab32503
Rabbit monoclonal anti-Caspase-3 (D3R6Y)	CST	Cat#14220
Rabbit monoclonal anti-Caspase-8 (D35G2)	CST	Cat#4790
Rabbit monoclonal anti-Caspase-9	CST	Cat#9502

Rabbit polyclonal anti-FDX1	Proteintech	Cat#12592-1-AP
Rabbit polyclonal anti-LIAS	Proteintech	Cat#11577-1-AP
Rabbit monoclonal anti-LAMP1	Abcam	Cat#ab278043
Mouse monoclonal anti-ACO2	Proteintech	Cat#67509-1-Ig
Mouse monoclonal anti-ETFDH	Abcam	Cat#ab131376
Rabbit monoclonal anti-NDUFV1	Abcam	Cat#ab308175
Rabbit monoclonal anti-NDUFS8	Abcam	Cat#ab170936
Rabbit polyclonal anti-Lipoic acid	Abcam	Cat#ab58724
Mouse monoclonal anti-DLAT	CST	Cat#12362
Mouse monoclonal anti-β-Actin	Proteintech	Cat#66009-1-Ig
Rabbit monoclonal anti-TOMM40	Abcam	Cat#ab185543
Mouse monoclonal anti-cytochrome C	Abcam	Cat#ab13575
Rabbit polyclonal anti-Ki67	Proteintech	Cat#27309-1-AP
Mouse monoclonal anti-4-HNE	Abcam	Cat#ab48506
Rabbit monoclonal anti-cleaved caspase-3 (Asp175)	CST	Cat#9661
Rabbit polyclonal anti-FDX1	Proteintech	Cat#12592-1-AP
Mouse monoclonal anti-HIF-1α	Abcam	Cat#ab279654
CoraLite488-conjugated goat anti-rabbit IgG(H+L) secondary antibody	Proteintech	Cat#SA00013-2
CoraLite594-conjugated goat anti-mouse IgG(H+L) secondary antibody	Proteintech	Cat#RGAM004
HRP-labeled goat anti-rabbit IgG (H+L) antibody	ZSGB-Bio	Cat#ZB-2306
HRP-labeled goat anti-mouse IgG (H+L) antibody	ZSGB-Bio	Cat#ZB-2305
**Cell lines and primary cells**	**Source**	**Identifier**
Human CRC cell line HCT116	Cyagen	Cat#H1-0401
Human CRC cell line RKO	Cyagen	Cat#H1-0601
Murine colon cancer line MC38	Cyagen	Cat#M1-0401
Human normal colon epithelial cell line NCM460	Cyagen	Cat#H1-3701
Human lung cancer cell line A549	Cyagen	Cat#H0-0701

Human breast cancer cell line MCF7	Cyagen	Cat#H2-0201
Human liver cancer cell line HepG2	Procell	Cat#CL-0103
Human melanoma cell line SK-MEL-5	BioVector	Cat#3576432
Human liver sinusoidal endothelial cells	This paper	N/A
Human pancreatic islet cells	This paper	N/A
Human umbilical vein endothelial cells	This paper	N/A
**Mice**	**Source**	**Identifier**
C57BL/6 mice	Weitonglihua	219
BALB/c nude mice	Weitonglihua	401
NOG (NOD/Shi-scid/IL-2Rγnull) mice	Weitonglihua	408

Table S2. Primer information

Genes	Forward Sequence	Reverse Sequence
ABAT	GCCTCTGATGAAGACGGAAGTC	CATTCGGTTGCCGTCCACATCA
ACO2	CAATCGTCACCTCCTACAACAGG	GTCTCTGGGTTGAACTTGAGGG
AIFM3	AGGAGGTTGTGCTGAAGAGCAG	AAACCGATGCCGCTTTGCCTCA
CDK5RAP1	AGAGTGGAAGCAGCCGTGTGTT	GATCTTCCTCCGTCTCACCACA
CISD1	CCTTCACATCCAGAAAGACAACC	CTCTTCGTTATGTTTTGTGTGAGC
CISD3	TGGCAGGGAAAACCTACAGGTG	GAGATAGGCCAGTGCGTTGGAA
ETFDH	GGAAACACCATCCTAGCATTCGG	CCACCAGGAAAGGTGAGTTTTGG
FDX1	GGGACAATGTTCTTCACCTGGC	AGGAGATCCAGGTGGTCTTCAC
FECH	TCTTCTTGGACCGAGACCTCATG	TCCAATCCTGCGGTACTGCTCT
GLRX2	TGGAAACCAGTTCCAAGATGCTC	GTGAAGCCTATGAGTGTCAGTTG
LIAS	GCCAAGAAGGTTCAGCCTGATG	GTCTACATCTGCCTCACGAAGTG
MOCS1	TCACAGGTGGAGAGCCGCTTAT	CACTGAGACCAGCCTTCTGAAG
NDUFS1	GAGTGGACTCTGACACCTTATGC	ACAACATCTGCCTCTTCCACACC
NDUFS8	CCACCATCAACTACCCGTTCGA	TTGGCTCAGCCTCGATGGTGAT
NDUFV1	TGTGTGAGACGGTGCTGATGGA	CGATGGCTTTCACGATGTCCGT
NDUFV2	ACTCTGACAGCATACTGGAGGC	ACCATTGGTGCGTTCACACAGG
NDUFS7	AGGCACGAGGTGTCCATCAGAG	CAGTTGACGAGGTCATCCAGCT
RSAD1	GGACTTACTGGCAGTGTGGTCA	CCTCCTTCATCCAGTTGTCAGG
SDHB	GCAGTCCATAGAAGAGCGTGAG	TGTCTCCGTTCCACCAGTAGCT
UQCRFS1	CCTGTGTTGGACCTGAAGCGG	CAGAGAAGTCAGGCACCTTGATG
ACP1	CCAGTATAGCGACATGCCTCCT	GGTCCAAACTGTCTAAGCCCAG
BOLA3	GAAAAGTTTCCACGAGCTACAGC	CATCTGGTGCTGCTGGACAGTT
FDX2	GGGACAATGTTCTTCACCTGGC	AGGAGATCCAGGTGGTCTTCAC
FDXR	TACAACGGGCTTCCTGAGAACC	GTCCGTTCTCTGGCACAAAAGG
FXN	GCCTCAACCAGATTTGGAATGTC	AGTCCAGCGTTTCCTCTGCTAG
GLRX5	TCAGCAACGCCGTGGTGCAGA	TTGAGGTACACTTGCGGGATGG
GRPE1	GCAGACACTGAGAACTTACGGC	CTGTGTTGCCTTCTCCAGAACG

HSC20	CGAGACTACTTCAGCCTTATGGA	AATCTGGGTGGACAAGACGCTG
HSPA9	GCCTTGCTACGGCACATTGTGA	CTGCACAGATGAGGAGAGTTCAC
IBA57	GCTCTATGACGTCATCTTGTACG	CTGTATAGCGCGAGGTGCTTCT
ISCA1	TGTCCGAACCAGGGGCTGTAAT	AGTGTTAGCTGTGCTTTCTTTTCG
ISCA2	ATTCCTCAGGCTGCAAGTGGAG	GCCAAGCTATCAGAGTCAACCAC
ISD11	CTGTCAGGAGGATAAGAGATGCC	TCGACGAATTACTCCAAGGTCTC
NFS1	GGAAAGTCTGCTGATGGCACTG	GCGCTAAATCCTCATCAGTGCC
NFU1	GAGGAAACACCTTCAGGAGAAGC	CCTCCATCTTCCTGCACAGTTG

Table S3. UPLC-MS/MS conditions

**UPLC**	
Column	BEH C18 1.7 μM analytical column (2.1 ×100 mm)
Column temperature	40 ℃
Sample manager temperature	10 ℃
Mobile phase	A = 5mM DIPEA; B = ACN: IPA = 7:3
Elution conditions	0-1 min (1% B), 1-9.5 min (1-15% B), 9.5-13 min (15-62% B), 13-14 min (62-100% B), 14-16 min (100% B), 16-16.2 min (100-1% B), 16.2-18 min (1% B)
Flow rate	0.3 mL/min
Injection volume	5.0 μL
**MS**	
Capillary voltage	3 kV (ESI-)
Source temperature	150 ℃
Desolvation temperature	500 ℃
Desolvation gas flow	1000 L/Hr
